# Supplementary material for: Psychosocial safety climate (PSC) and working conditions, predictors of mental health and antidepressant and opioid use in Australia: a study protocol for longitudinal data linkage
Source: BMJ Open. 2023 Dec 13;13(12):e074235. doi: 10.1136/bmjopen-2023-074235 (PMC10729104; doi:10.1136/bmjopen-2023-074235)
Supplement: Supplementary data [file bmjopen-2023-074235supp001.pdf]

UniSA | Justice & Society

University of South Australia
Australia's University of Enterprise

Australian Workplace Barometer Survey (2021)

INFORMATION SHEET

Welcome to the Australian Workplace Barometer (AWB) survey.

Professor Maureen Dollard, Director of the PSC Observatory, for the University of South Australia is conducting the study with funding from the Australian Research Council.

This Australian Research Council national study aims to improve the health of Australian workers. Questions will be asked about working conditions, job security, psychological health, physical health, heart health, work engagement, satisfaction, fatigue, and workers' compensation.

We previously contacted you in 2014 about the Australian Workplace Barometer survey and you kindly gave us permission to contact you again regarding future surveys. Please complete the double-sided survey attached and return in the enclosed reply-paid envelope. Alternatively, if you wish to complete the survey online, please type the following link into your web browser http://bit.ly/AWB-Survey, ensuring to insert your Participant ID below into the survey link and complete online. Once you have completed the survey you will go into the draw to win $100.

For additional information about the Australian Workplace Barometer, please visit website https://bit.ly/AWB-InfoSheet

We greatly appreciate your support and contribution to the AWB project. If you experience any issues, please contact the AWB Team on 0422 643 737.

Kind regards
Dr Ali Afsharian
Research Associate
PSC Observatory / Centre for Workplace Excellence (CWeX)
University of South Australia
Email: ali.afsharian@unisa.edu.au

Participant's ID:

Australian Workplace Barometer (AWB) Survey

1. Are you still in paid employment or self-employed or a sub-contractor?
   - o Employed (skip to Q.3)
   - o Sub-contractor (skip to Q.5)
   - o Self-employed
   - o No longer in paid employment or self-employed or a subcontractor (go to Q.2)

   If answered 'Self-employed', you will not be able to complete the remainder of the survey as the survey is for current employed workers and their workplace conditions. Thank you for your time.

2. What is the reason that you are no longer in paid employment or self-employed or a sub-contractor?
   - o Retired
   - o Made redundant
   - o Maternity leave
   - o Student
   - o Other (specify): ___________________

   You will not be able to complete the remainder of the survey as the survey is for current workers and their workplace conditions. Thank you for your time.

3. Is your employer currently receiving Job Keeper benefits for you?
   - o Yes (go to Q.4)
   - o No (skip to Q.6)
   - o Not sure

4. Are you currently participating in work-related tasks or duties?
   - o Yes (skip to Q.6)
   - o No

   If 'No', you will not be able to complete the remainder of the survey as the survey is for current workers and their workplace conditions. Thank you for your time.

5. Has your current contract involved working for the same employer for the past 4 months or longer?
   - o Yes
   - o No

   If 'Yes', would you prefer an alternative employment status?
   - o Permanent full-time
   - o Permanent part-time
   - o Casual/temporary (no annual/sick leave)
   - o Fixed-term contract
   - o Other (specify): ____________________

   (skip to Q.7)

   If 'No', you will not be able to complete the remainder of the survey as the survey is for current employed workers and their workplace conditions. Thank you for your time.

Employment

The following questions relate to your main job, that is the one in which you work most hours.

6. What is your employment status?
   - o Permanent full-time
   - o Permanent part-time
   - o Casual/temporary (no annual/sick leave)
   - o Fixed-term contract
   - o Other (specify): _____________________

7. Has your employment status changed due to the Covid-19 pandemic?
   - o No
   - o Yes, from full-time to part time
   - o Yes, from full-time to casual
   - o Other (specify): __________________

8. At this moment, which do you best identify with?
   - o The majority of my work is done remotely
   - o I normally split my time between home and or on-site, but now I'm only working remotely
   - o I normally work on-site but now I'm working remotely
   - o I am still working on-site

9. About how long have you been working for your current employer?
Enter years: ________________
Enter months: ________________

10. What is the main business, industry or service of your employer?
- o Accommodation, cafes and restaurants
- o Agriculture, forestry and fishing
- o Communications services
- o Construction
- o Cultural and recreational services
- o Education
- o Electricity, gas and water supply
- o Finance and insurance
- o Government administration and defence
- o Health and community services
- o Manufacturing
- o Mining
- o Personal and other services
- o Property and business services
- o Retail trade
- o Transport and storage
- o Wholesale trade
- o Other (specify): ____________

11. Is your employers business a…..?
- o A commonwealth or federal government department or agency
- o A state or local government agency
- o A not-for-profit, religious, or community organisation
- o A private sector business
- o Something else (specify): ____________

12. With regard to number of employees – what size organisation do you work in?
- o MICRO (less than 2)
- o SMALL (2-19)
- o MEDIUM (20-200)
- o LARGE (200+)
- o Don't know

13. Are you a member of a union or employee organisation that looks after employees' interests (e.g., Nursing Federation, Police Association)?
- o Yes
- o No
- o Don't know

14. Are you paid for any overtime you do?
- o Always
- o Sometimes
- o Never
- o Don't do any overtime

15. Which time period do you usually work?
- o Day
- o Evening
- o Night
- o Rotating shifts
- o Other (specify): ________________

16. Have you ever put in a worker's compensation claim?
- o Yes (go to Q.17)
- o No (skip to Q.21)

17. Did you lodge this claim in the last 12 months?
- o Yes (go to Q.18)
- o No (skip to Q.21)

18. Was the most recent claim for a stress or psychological injury?
- o Yes (go to Q.19)
- o No (skip to Q.20)

19. Reason for the most recent claim stress or psychological injury claim?
- o Exposure to a traumatic event
- o Exposure to workplace or occupational violence
- o Work related harassment and/or workplace bullying
- o Work Pressure
- o Other mental stress factors
- o Other harassment
- o Other (specify): ______________

(skip to Q.21)

20. Was the most recent claim for neck, back, or joint pain or muscle injury?
- o Yes
- o No

Job Demands - Psychological Demands
Below are some statements about your work. Could you please tick below if you disagree or agree and to what level ...?

	Strongly Disagree	Disagree	Agree	Strongly Agree
**21.** My job requires working very fast.	○	○	○	○
**22.** My job requires working very hard.	○	○	○	○
**23.** I am not asked to do an excessive amount of work.	○	○	○	○
**24.** I have enough time to get the job done.	○	○	○	○
**25.** Some demands I face at work are in conflict with other demands at work.	○	○	○	○

Job Demands - Physical Demands
Below are some statements about your work. Could you please tick below if you disagree or agree and to what level ...?

	Strongly Disagree	Disagree	Agree	Strongly Agree
**26.** My job requires lots of physical effort.	○	○	○	○
**27.** I am often required to move or lift very heavy loads on my job.	○	○	○	○
**28.** I am often required to work for long periods with my body in physically awkward positions.	○	○	○	○

Workplace Physical Risk Factors
I am required to:

	Almost never	Sometimes	Often	Almost always
**29.** Keep repeating the same movements, every minute or so.	○	○	○	○
**30.** Lift or carry things that are moderately (or very) heavy.	○	○	○	○
**31.** Push or pull things using some force.	○	○	○	○
**32.** Work in twisted or awkward postures.	○	○	○	○
**33.** Squat or kneel while you work.	○	○	○	○

Emotional Demands

	Strongly Disagree	Disagree	Agree	Strongly Agree
**34.** My work is emotionally demanding.	○	○	○	○
**35.** My work places me in emotionally challenging situations.	○	○	○	○
**36.** My work requires suppressing my genuine emotion.	○	○	○	○
**37.** I have to act the way people think a person in my position should act.	○	○	○	○

Crispin CN, *et al. BMJ Open* 2023; 13:e074235. doi: 10.1136/bmjopen-2023-074235

Organisational Characteristics - Demands Organisational Change
The next set of questions concerns things that you may or may not experience in your work.

	Strongly Disagree	Disagree	Agree	Strongly Agree
**38.** In your company/organisation, there have been changes such as restructuring, downsizing, and layoffs that have significantly affected your job.	○	○	○	○
**39.** In your company/organisation, new policies and procedures designed to cut costs are constantly being introduced where you work.	○	○	○	○
**40.** In your company/organisation, there is frequent management turnover.	○	○	○	○

Organisational Harassment
Sometimes in your workplace you may experience or witness harassment from your manager/supervisor or colleagues. Can you please answer with the best response to these statements?

	Very rarely/never	Rarely	Sometimes	Often	Very often/ always
**41.** I have experienced unwanted sexual advances.	○	○	○	○	○
**42.** I have experienced discomfort listening to sexual humour.	○	○	○	○	○
**43.** I have experienced unfair treatment because of my gender.	○	○	○	○	○
**44.** Negative comments have been made regarding my ethnic or racial background.	○	○	○	○	○
**45.** I have been sworn at and or yelled at.	○	○	○	○	○
**46.** I have been humiliated in front of others.	○	○	○	○	○
**47.** I have experienced being physically assaulted and or threatened by members of the organisation.	○	○	○	○	○
**48.** I have experienced being physically assaulted and or threatened, by a client or patient.	○	○	○	○	○

Organisational Bullying

Bullying is a problem at some workplaces and for some workers. To label something as bullying, the offensive behaviour has to occur repeatedly over a period of time, and the person confronted has to experience difficulties defending him or herself. The behaviour is not bullying if two parties of approximate equal "strength" are in conflict or the incident is an isolated event.

49. Have you been subjected to bullying at the workplace during the last six months?

- ○ Yes (go to Q.50)
- ○ No (skip to Q.53)

50. how <u>often</u> were you exposed to these bullying behaviours overall?

- ○ Daily
- ○ At least once per week
- ○ At least once per month
- ○ Rarely
- ○ Never

51. How <u>long</u> were you exposed to these bullying behaviours overall?

- ○ Less than one month
- ○ 1-6 months
- ○ 7-12 months
- ○ 1-2 years
- ○ More than 2 years

52. Was the bully a …?

- ○ A supervisor
- ○ A co-worker
- ○ Neither
- ○ Both

Work-Family Conflict

Below are some statements about how work can affect your home and personal life.
Could you please tick below if you disagree or agree and to what level ...?

	Strongly Disagree	Disagree	Slightly Disagree	Neither Agree nor Disagree	Slightly Agree	Agree	Strongly Agree
**53.** The demands of my work interfere with my home and family life.	○	○	○	○	○	○	○
**54.** My job produces strain that makes it difficult to fulfil family duties.	○	○	○	○	○	○	○
**55.** Due to work-related duties, I have to make changes to my plans for family activities.	○	○	○	○	○	○	○

Crispin CN, *et al. BMJ Open* 2023; 13:e074235. doi: 10.1136/bmjopen-2023-074235

Psychosocial Safety Climate
The questions and statements in this section are about the psychological health and safety at your workplace. Could you please tick below if you disagree or agree and to what level ...?

	Strongly Disagree	Disagree	Neither Disagree or Agree	Agree	Strongly Agree
**56.** In my workplace senior management acts quickly to correct problems/issues that affect employees' psychological health.	○	○	○	○	○
**57.** Senior management acts decisively when a concern of an employees' psychological status is raised.	○	○	○	○	○
**58.** Senior management show support for stress prevention through involvement and commitment.	○	○	○	○	○
**59.** Psychological well-being of staff is a priority for this organisation.	○	○	○	○	○
**60.** Senior management clearly considers the psychological health of employees to be of great importance.	○	○	○	○	○
**61.** Senior management considers psychological health to be as important as productivity.	○	○	○	○	○
**62.** There is good communication here about psychological safety issues which affect me.	○	○	○	○	○
**63.** Information about workplace psychological well-being is always brought to my attention by my manager/supervisor.	○	○	○	○	○
**64.** My contributions to resolving occupational health and safety concerns in the organisation are listened to.	○	○	○	○	○
**65.** Participation and consultation in psychological health and safety occurs with employees', unions and health and safety representatives in my workplace.	○	○	○	○	○
**66.** Employees are encouraged to become involved in psychological safety and health matters.	○	○	○	○	○
**67.** In my organisation, the prevention of stress involves all levels of the organisation.	○	○	○	○	○

Crispin CN, *et al. BMJ Open* 2023; 13:e074235. doi: 10.1136/bmjopen-2023-074235

Resources - Skill Discretion

The next set of questions concerns some characteristics of your work. Please answer with the best response that suits your workplace.

	Strongly Disagree	Disagree	Agree	Strongly Agree
**68.** My job requires that I learn new things.	○	○	○	○
**69.** My job involves a lot of repetitive work.	○	○	○	○
**70.** My job requires me to be creative.	○	○	○	○
**71.** My job requires a high level of skill.	○	○	○	○
**72.** I get to do a variety of different things in my job.	○	○	○	○
**73.** I have an opportunity to develop my own special abilities.	○	○	○	○

Decision Authority

	Strongly Disagree	Disagree	Agree	Strongly Agree
**74.** My job allows me to make a lot of decisions on my own.	○	○	○	○
**75.** In my job, I have very little freedom to decide how I do my work.	○	○	○	○
**76.** I have a lot to say about what happens in my job.	○	○	○	○

Macro-Decision Latitude

	Strongly Disagree	Disagree	Agree	Strongly Agree
**77.** In my company/organisation, I have significant influence over decisions made by my work team or department.	○	○	○	○
**78.** In my company/organisation, all parties are represented for major decisions.	○	○	○	○
**79.** In my company/organisation, we are always informed well in advance about major decisions.	○	○	○	○

Supervisor Social Support

	Strongly Disagree	Disagree	Agree	Strongly Agree
**80.** My supervisor/manager is concerned about the welfare of those under him/her.<br>I have no supervisor/manager (skip to Q.83)	○	○	○	○
**81.** My supervisor/manager is helpful in getting the job done.	○	○	○	○
**82.** I am treated with respect by my supervisor/manager.	○	○	○	○

Crispin CN, *et al. BMJ Open* 2023; 13:e074235. doi: 10.1136/bmjopen-2023-074235

**Co-worker Social Support**				
	**Strongly Disagree**	**Disagree**	**Agree**	**Strongly Agree**
**83.** People I work with are friendly.<br>I have no coworkers (skip to Q.86)	○	○	○	○
**84.** People I work with are helpful in getting the job done.	○	○	○	○
**85.** I am treated with respect by my co-workers.	○	○	○	○

**Recovery**							
	**Strongly Disagree**	**Disagree**	**Slightly Disagree**	**Neither Agree nor Disagree**	**Slightly Agree**	**Agree**	**Strongly Agree**
**86.** I rarely recover my strength fully between work periods.	○	○	○	○	○	○	○
**87.** I'm often still feeling fatigued from one work period by the time I start the next one.	○	○	○	○	○	○	○

**Organisational Characteristics - Resources Organisational Rewards**				
	**Strongly Disagree**	**Disagree**	**Agree**	**Strongly Agree**
**88.** Considering all my efforts and achievements, I receive the respect and prestige I deserve at work.	○	○	○	○
**89.** Considering all my efforts and achievements, my job prospects are adequate.	○	○	○	○
**90.** Considering all my efforts and achievements, my salary/income is adequate.	○	○	○	○
**91.** My job security is poor.	○	○	○	○

Psychosocial Safety Climate Policy

92. Does your establishment have a procedure to deal with work-related stress?
- Yes
- No
- Work-related stress is not an issue in our establishment
- Don't know

93. Is there a procedure in place to deal with bullying or harassment?
- Yes
- No
- These problems are not an issue in our establishment
- Don't know

94. And do you have a procedure to deal with work-related violence?
- Yes
- No
- Work-related violence is not an issue in our establishment
- Don't know

95. What about the role of employees: Have they been consulted regarding measures to deal with psychosocial risks?
- Yes
- No
- Don't know

96. Are employees encouraged to participate actively in the implementation and evaluation of the measures?
- Yes
- No
- Don't know

Job Redesign

In the last 3 years, has your establishment used any of the following measures to deal with psychosocial risks?

97. Changes to the way work is organised?
- Yes
- No
- Don't know

98. A redesign of the work area?
- Yes
- No
- Don't know

99. Set-up of a conflict resolution procedure?
- Yes
- No
- Don't know

Engagement & Happiness

We also need to know about your levels of engagement in your work. Please answer with the best response.

Vigour

100. At my work, I feel bursting with energy.
- Never
- 1-3 times per year or less
- Once a month
- A few times a month
- Once a week
- A few times a week
- Every day

Dedication

101. I am enthusiastic about my job.
- Never
- 1-3 times per year or less
- Once a month
- A few times a month
- Once a week
- A few times a week
- Every day

Absorption

102. I am immersed in my work.
- Never
- 1-3 times per year or less
- Once a month
- A few times a month
- Once a week
- A few times a week
- Every day

Happiness

The next question is about how you rate your general happiness. On a scale from 0 to 10 where 0 means "extremely unhappy" and 10 means "extremely happy"……

Overall, how happy are you?
- Enter no. ____________________
- Don't know

Crispin CN, *et al. BMJ Open* 2023; 13:e074235. doi: 10.1136/bmjopen-2023-074235

Mental Health
We also need to know some information about your mental and physical health. Please answer as best you can by selecting the best option.
Emotional Exhaustion

	Never	A few times a year or less	Once a month or less	A few times a month	Once a week	A few times a week	Every day
**103.** I feel emotionally drained from my work.	○	○	○	○	○	○	○
**104.** I feel used up at the end of the workday.	○	○	○	○	○	○	○
**105.** I feel tired when I get up in the morning and have to face another day on the job.	○	○	○	○	○	○	○
**106.** Working all day is a strain for me.	○	○	○	○	○	○	○
**107.** I feel burned out from my work.	○	○	○	○	○	○	○

Psychological Distress

	None of the time	A little of the time	Some of the time	Most of the time	All of the time
**108.** In the past four weeks, about how often did you feel tired out for no good reason?	○	○	○	○	○
**109.** In the past four weeks, about how often did you feel nervous?	○	○	○	○	○
**110.** In the past four weeks, about how often did you feel so nervous that nothing could calm you down?	○	○	○	○	○
**111.** In the past four weeks, about how often did you feel hopeless?	○	○	○	○	○
**112.** In the past four weeks, about how often did you feel restless or fidgety?	○	○	○	○	○
**113.** In the past four weeks, about how often did you feel so restless you could not sit still?	○	○	○	○	○
**114.** In the past four weeks, about how often did you feel depressed?	○	○	○	○	○
**115.** In the past four weeks, about how often did you feel everything was an effort?	○	○	○	○	○
**116.** In the past four weeks, about how often did you feel so sad that nothing could cheer you up?	○	○	○	○	○
**117.** In the past four weeks, about how often did you feel worthless?	○	○	○	○	○

Crispin CN, *et al. BMJ Open* 2023; 13:e074235. doi: 10.1136/bmjopen-2023-074235

**Depression**				
	**Not at all**	**Several days**	**More than half the days**	**Nearly every day**
**118.** During the last month, how often were you bothered by little interest or pleasure in doing things?	○	○	○	○
**119.** During the last month, how often were you bothered by feeling down, depressed, or hopeless?	○	○	○	○
**120.** During the last month, how often were you bothered by trouble falling or staying asleep OR sleeping too much?	○	○	○	○
**121.** During the last month, how often were you bothered by feeling tired or having little energy?	○	○	○	○
**122.** During the last month, how often were you bothered by poor appetite or overeating?	○	○	○	○
**123.** During the last month, how often were you bothered by feeling bad about yourself-or that you are a failure or have let yourself or your family down?	○	○	○	○
**124.** During the last month, how often were you bothered by trouble concentrating on things, such as reading a newspaper or watching television?	○	○	○	○
**125.** During the last month, how often were you bothered by moving or speaking so slowly that other people could have noticed OR – the opposite – being so fidgety or restless that you have been moving around a lot more than usual?	○	○	○	○
**126.** During the last month, how often were you bothered by thoughts that you would be better off dead or thoughts of hurting yourself in some way?	○	○	○	○

Stigma and Discrimination

127. In the last 12 months…
Do you think you have had emotional or mental health problems?
- ○ Yes (go to Q.128)
- ○ No (skip to Q.135)
- ○ Don't know (go to Q.135)

128. In the last 12 months…
Have you ever discussed your anxiety or depression with your supervisors and or managers?
- ○ Yes
- ○ No

129. Have you ever discussed your anxiety or depression with your co-worker?
- ○ Yes
- ○ No

	Not at all	A little	Moderately	A lot	Don't know
**130.** Have any of your supervisors and or managers avoided you because of the emotional or mental health problems?	○	○	○	○	○
**131.** Have any of your coworkers avoided you because of the emotional or mental health problems?	○	○	○	○	○
**132.** Have any of your supervisors and or managers treated you unfairly or discriminated against you because of your mental health problems?	○	○	○	○	○
**133.** Have any of your co-workers treated you unfairly or discriminated against you because of your mental health problems?	○	○	○	○	○
**134.** Because of your mental health or emotional problems have you been treated more positively by your employer?	○	○	○	○	○

Crispin CN, *et al. BMJ Open* 2023; 13:e074235. doi: 10.1136/bmjopen-2023-074235

Life Events

We now would like you to think about major events that have happened in your life over the past 12 months.

135. Did any of these events happen to you in the past 12 months? (please tick any event that applies to you)

- ◻ Death of spouse or child
- ◻ Separated from spouse or long-term partner
- ◻ Major worsening in financial situation (e.g., went bankrupt)
- ◻ Serious personal injury or illness to self
- ◻ Victim of physical violence (e.g., assault)
- ◻ Got married
- ◻ Partner or I gave birth to, or adopted, a new child
- ◻ Major improvement in financial situation (e.g., won lottery, received an inheritance)

136. Has a doctor ever diagnosed you with depression or anxiety?

- o Yes
- o No

137. Have you taken any prescription medications for your mental health in the last 12 months?

- o Yes
- o No
- o Don’t know

138. Have you taken any of the following prescription medications in the past 12 months? (please tick any that apply to you)

- ◻ Sleeping tablets or capsules
- ◻ Tablets or capsules for anxiety or nerves
- ◻ Tranquillisers
- ◻ Antidepressants
- ◻ Mood stabilisers
- ◻ Other medications for your mental health
- ◻ None of the above
- ◻ Don’t know

139. Have you taken this in the last month?

- ◻ Sleeping tablets or capsules
- ◻ Tablets or capsules for anxiety or nerves
- ◻ Tranquillisers
- ◻ Antidepressants
- ◻ Mood stabilisers
- ◻ Other medications for your mental health
- ◻ None of the above
- ◻ Don't know

Crispin CN, *et al. BMJ Open* 2023; 13:e074235. doi: 10.1136/bmjopen-2023-074235

Physical Health - General Health

140. During the past 7 days how much were you bothered by back or neck pain?
- Not at all
- A little
- Some
- A lot

141. During the past 7 days how much were you bothered by pain in your arms, legs, or joint areas like your knee or hips?
- Not at all
- A little
- Some
- A lot

142. During the past 7 days how much were you bothered by muscle soreness?
- Not at all
- A little
- Some
- A lot

143. In general, would you say your health is….?
- Excellent
- Very good
- Good
- Fair
- Poor

144. Have you had a significant physical injury from inside of work in the past 12 months?
- Yes
- No

145. Did you make a compensation claim?
- Yes
- No

Cardio-Vascular Health

146. In the past two years have you had your blood pressure taken by a health professional or used a self-administered machine?
- Yes
- No

IF yes, what was your blood pressure?
- Low
- Normal
- High
- Don't know

147. In the past two years have you consulted a health professional with regard to chest pain, or any other cardiovascular related health problem – such as myocardial infarction; angina; stroke; or hypertension?
- Yes
- No
- Don't know

IF yes, what was the diagnosis?
- Myocardial Infarction
- Angina
- Stroke
- Hypertension
- Other (specify)/Or if more than one, mention here _______________
- Don't know
- Not Stated
- Nothing

Work Outcomes - Job Satisfaction

148. Taking everything into consideration, how do you feel about your job as a whole?
- I'm extremely dissatisfied
- I'm very dissatisfied
- I'm moderately dissatisfied
- I'm not sure
- I'm moderately satisfied
- I'm very satisfied
- I'm extremely satisfied

Work Outcomes – Intention to Leave

149. Over this year I intend to leave this organisation.
- Strongly Agree
- Agree
- Neutral
- Disagree
- Strongly Disagree

Work Outcomes - Productivity

150. On a scale from 0 to 10 where 0 is the worst job performance anyone could have at your job and 10 is the performance of a top worker, how would you rate the usual performance of most workers in a job similar to yours?
Enter number here: _____________

151. Using the same 0-to-10 scale, how would you rate your overall job performance on the days you worked during the past 4 weeks (28 days)?
Enter number here: ______________

152. Using the same 0-to-10 scale, where 0 is no affect and 10 is the most affect, during the last 4 weeks (28 days) how much did your health problems affect your productivity at work?
Enter number here: _________________

Work Outcomes - Absenteeism

153. How many hours does your employer expect you to work in a typical 7-day week?
Note: If it varies, estimate the average. If more than 97, enter 97. If none - enter 0.
Enter hours: ______________________

154. In the past 4 weeks (28 days), how many days did you, miss an entire work day because of problems with your physical or mental health? (Please include only days missed for your own health not someone else's health?
Enter number of days (0-28): _______

155. About how many hours altogether did you work in the past 4 weeks?
Enter number of hours: ____________

Employment

The next questions are about your main job, that is the one in which you work most hours.

156. What is your occupation in this job – what are your main duties?
- ○ Managers or administrator (go to Q.157)
- ○ Professional work (go to Q.158)
- ○ Technician or associate professional (go to Q.159)
- ○ Tradesperson or related work (go to Q.160)
- ○ Advanced clerical, sales or service work (go to Q.161)
- ○ Intermediate clerical, sales or service work (go to Q.162)
- ○ Intermediate plant operator/transport (go to Q.163)
- ○ Elementary clerical, sales or service work (go to Q.164)
- ○ Labourer or related work (go to Q.165)
- ○ Other (please specify) ________________ (go to Q.166)

157. What best describes this work?
- ○ General Managers and Administrators
- ○ Miscellaneous Generalist Managers
- ○ Resource Managers
- ○ Engineering, Distribution and Process Managers
- ○ Sales and Marketing Managers
- ○ Miscellaneous Specialist Managers
- ○ Farmers and Farm Managers
- ○ Other (please specify) ________________ (go to Q.166)

158. What best describes this work?
- ○ Natural and Physical Science Professionals
- ○ Building and Engineering Professionals
- ○ Accountants, Auditors and Corporate Treasurers
- ○ Sales, Marketing and Advertising Professionals
- ○ Computing Professionals
- ○ Miscellaneous Business and Information Professionals
- ○ Medical Practitioners
- ○ Nursing Professionals
- ○ Miscellaneous Health Professionals
- ○ School Teachers
- ○ University and Vocational Education Teachers
- ○ Miscellaneous Education Professionals
- ○ Social Welfare Professionals
- ○ Miscellaneous Social Professionals
- ○ Artists and Related Professionals
- ○ Miscellaneous Professionals
- ○ Other (please specify) ________________ (go to Q.166)

Crispin CN, *et al. BMJ Open* 2023; 13:e074235. doi: 10.1136/bmjopen-2023-074235

159. What best describes this work?
- o Medical and Science Technical Officers
- o Building and Engineering Associate Professionals
- o Finance Associate Professionals
- o Miscellaneous Business and Administration Associate Professionals
- o Shop Managers
- o Hospitality and Accommodation Managers
- o Miscellaneous Managing Supervisors (Sales and Service)
- o Enrolled Nurses
- o Welfare Associate Professionals
- o Miscellaneous Health and Welfare Associate Professionals
- o Police Officers
- o Miscellaneous Associate Professionals
- o Other (please specify) _______________ (go to Q.166)

160. What best describes this work?
- o Mechanical Engineering Tradespersons
- o Fabrication Engineering Tradespersons
- o Automotive Tradespersons
- o Electrical and Electronics Tradespersons
- o Structural Construction Tradespersons
- o Final Finishes Construction Tradespersons
- o Plumbers
- o Food Tradespersons
- o Skilled Agricultural Workers
- o Horticultural Tradespersons
- o Printing Tradespersons
- o Wood Tradespersons
- o Hairdressers
- o Textile, Clothing and Related Tradespersons
- o Miscellaneous Tradespersons and Related Workers
- o Other (please specify) _______________ (go to Q.166)

161. What best describes this work?
- o Secretaries and Personal Assistants
- o Advanced Numerical Clerks
- o Miscellaneous Advanced Clerical and Service Workers
- o Other (please specify) _______________ (go to Q.166)

162. What best describes this work?
- o General Clerks
- o Keyboard Operators
- o Receptionists
- o Intermediate Numerical Clerks
- o Material Recording and Despatching Clerks
- o Miscellaneous Intermediate Clerical Workers
- o Intermediate Sales and Related Workers
- o Carers and Aides
- o Hospitality Workers
- o Miscellaneous Intermediate Service Workers
- o Other (please specify) _______________ (go to Q.166)

163. What best describes this work?
- o Mobile Plant Operators
- o Intermediate Stationary Plant Operators
- o Intermediate Textile, Clothing and Related Machine Operators
- o Miscellaneous Intermediate Machine Operators
- o Road and Rail Transport Drivers
- o Intermediate Mining and Construction Workers
- o Miscellaneous Intermediate Production and Transport Workers
- o Other (please specify) _______________ (go to Q.166)

164. What best describes this work?
- o Elementary Clerks
- o Sales Assistants
- o Miscellaneous Elementary Sales Workers
- o Elementary Service Workers
- o Other (please specify) _______________ (go to Q.166)

165. What best describes this work?
- o Cleaners
- o Process Workers
- o Product Packagers
- o Mining, Construction and Related Labourers
- o Agricultural and Horticultural Labourers
- o Elementary Food Preparation and Related Workers
- o Miscellaneous Labourers and Related Workers
- o Other (please specify) _______________ (go to Q.166)

Now to finish with some general questions.

166. Do you identify as ….?
- o Male
- o Female
- o Non binary
- o Transgender Male
- o Transgender Female
- o Other (please specify): ______

167. Including yourself how many people aged 18 or over, who are currently in paid employment, live in this household?
Enter number: ____________________

168. How many children under 18 live in your household?
Enter number: __________________

169. What state do you live in?
- o NSW
- o WA
- o SA
- o ACT
- o TAS
- o NT
- o QLD
- o VIC
- o Other: _______________

170. What is the postcode of the house?
____

171. What town or suburb do you live in?
Enter town/suburb: ______________

172. What state do you work in?
- o NSW
- o WA
- o SA
- o ACT
- o TAS
- o NT
- o QLD
- o VIC
- o Outside of Australia
- o Other (please specify): ___________

173. The Australian Council of Trade Unions (ACTU) in partnership with The University of South Australia (UniSA) and Employers Mutual Limited (EML) are co-hosting the Mind Your Head Campaign funded by WorkSafe Victoria's WorkWell Mental Health Improvement.
Have you heard/received any information about this campaign? (please disregard if not from VIC)
- o Yes
- o No

If 'Yes' what was the source of this information
- o Workplace communications
- o Media communications
- o External/personal communications
- o Other (please specify): _____________

174. What is your marital status?
- o Married
- o Living with partner
- o Separated
- o Divorced
- o Widowed
- o Never married

175. What best describes the highest educational qualification you have obtained?
- o Still at school
- o Left school at 16 years or less
- o Left school after age 16
- o Left school after age 16 but still studying
- o Trade / Apprenticeship
- o Certificate / Diploma
- o Bachelor degree or higher
- o Other (specify): _______________

176. We are interested to know if there is any relation to income levels and job stress risk. Before tax is taken out, which of the following best describes your income from your main job in the last 12 months?
- o Up to $12,000
- o $12,001 - $20,000
- o $20,001 - $30,000
- o $30,001 - $40,000
- o $40,001 - $50,000
- o $50,001 - $60,000
- o $60,001 - $80,000
- o $80,001 - $100,000
- o $100,001 - $150,000
- o $150,001 - $200,000
- o More than $200,000

In order for us to make really good use of your answers it would be of great benefit to ask you the same questions at a later date. Would you be willing to be re-contacted about doing this survey again in about a year's time? You are not obliged to say yes if we call. You can decide then.

177. Are you willing to be re-contacted?
- Yes (full name, please): _______________
- Best contact number: _________________
- Email: ______________________________
- No

178. Could we have your current address?
- Yes (please enter the address in below) ______________
- No

In this kind of research, it is important to group data so it better reflects the work situation rather than the individual situation. By using organisational names we can group data and link workplace data to other worker health related data bases. We can answer questions like "In general, do organisations with high work pressure have more worker injuries?"

179. Could you give the name of your organisation so we can do this? The organisation/employer will be confidential in any report.
Enter organisation or business name ______________

180. What the suburb of your employer is?
Enter town/suburb: ________________

181. Is this the same organisation that you were at when we last interviewed you?
- Yes (skip to Q.183)
- No (go to Q.182)
- Can't remember (skip to Q.183)

182. If it is not the same organisation could we have the name and suburb of the organisation you worked at previously?
Enter organisation: _________________
Enter suburb: _____________________

We would also like to investigate how work conditions relate to mental health and related medications, such as "is work stress related to anti-depressant use in Australia?"
To facilitate this important research, we ask your permission to securely send your AWB personal information (along with other participant's information) to the Australian Institute of Health and Welfare to match to the Pharmaceutical Benefits Scheme data to create an anonymous research dataset.

183. Do you consent for us to securely send your personal AWB information (the information you have provided in this survey) to the Australian Institute of Health and Welfare to match to medication data in the Pharmaceutical Benefits Scheme and create an anonymous research dataset?
- Yes
- No

That was the final question. Thank you again for your time and support for the Australian Workplace Barometer project. We really appreciate it.

During this survey you were to think of some negative and positive experiences. You have also been asked some questions about if you have experienced any levels of depression. If you feel distressed as a result of any of these questions we offer you the telephone numbers below, should you need to discuss some of these concerns with a qualified professional.

Beyond Blue Support Service Line: 1300 224 636
Lifeline: 13 11 14
