# Supplementary material for: Psychosocial safety climate (PSC) and working conditions, predictors of mental health and antidepressant and opioid use in Australia: a study protocol for longitudinal data linkage
Source: BMJ Open. 2023 Dec 13;13(12):e074235. doi: 10.1136/bmjopen-2023-074235 (PMC10729104; doi:10.1136/bmjopen-2023-074235)
Supplement: Author's manuscript [file bmjopen-2023-074235.draft_revisions.pdf]

BMJ Open is committed to open peer review. As part of this commitment we make the peer review history of every article we publish publicly available.

When an article is published we post the peer reviewers' comments and the authors' responses online. We also post the versions of the paper that were used during peer review. These are the versions that the peer review comments apply to.

The versions of the paper that follow are the versions that were submitted during the peer review process. They are not the versions of record or the final published versions. They should not be cited or distributed as the published version of this manuscript.

BMJ Open is an open access journal and the full, final, typeset and author-corrected version of record of the manuscript is available on our site with no access controls, subscription charges or pay-per-view fees (http://bmjopen.bmj.com).

If you have any questions on BMJ Open's open peer review process please email info.bmjopen@bmj.com

BMJ Open

Tranquillizing work stress: Corporate climate and antidepressant and opioid use

Journal:	*BMJ Open*
Manuscript ID	bmjopen-2023-074235
Article Type:	Protocol
Date Submitted by the Author:	03-May-2023

Complete List of Authors:	Crispin, Cherie; University of South Australia, Justice & Society<br>Afsharian, Ali; University of South Australia, Justice & Society<br>Dollard, Maureen F.; University of South Australia,<br>Dormann, Christian; Johannes Gutenberg Universitat Mainz<br>Glozier, Nick; University of Sydney Brain and Mind Research Institute,<br>Gill, Tiffany; The University of Adelaide, Medical Specialities<br>Taylor, Anne; The University of Adelaide, School of Medicine

Keywords:	OCCUPATIONAL & INDUSTRIAL MEDICINE, EPIDEMIOLOGY, Health & safety < HEALTH SERVICES ADMINISTRATION & MANAGEMENT, Health policy < HEALTH SERVICES ADMINISTRATION & MANAGEMENT, MENTAL HEALTH, PUBLIC HEALTH

SCHOLARONE™ Manuscripts

Ms Cherie Crispin
Research Assistant
University of South Australia,
Centre for Workplace Excellence
160 Currie Street, Adelaide SA 5000

Dear Editor,

I forward the attached study protocol for peer-review and consideration for publication, on behalf the research team.

The objective of the research is to investigate whether antidepressant and opioid medication use leads to underestimation of the impact of work on mental ill-health. Specifically, this research will investigate the role of corporate climate (i.e. Psychosocial Safety Climate) in linking work stressors, mental distress, mental health problems, stigma, discrimination and medication use. Additionally, the research will look to understand whether baseline vulnerability places workers at risk for future exposure to work stressors and/or mental health problems and to determine what aspects of the work environment and working conditions could keep people with mental health problems engaged at work.

We take an innovative approach to investigate prescription usage and the role of corporate climate via data linkage from the Australian Workplace Barometer (AWB) population survey (occupational health cohort) to the national Pharmaceutical Benefits Scheme (PBS) (public data).

The research is significant as this new knowledge will show public policymakers, both nationally and internationally, where to focus future attention to prevent work-related mental health problems, such as depression and suicidal thoughts. The research will also identify which workplace processes can be changed to reduce unnecessary medication costs, which could significantly reduce the burden on both national and international health systems and on disability and workers compensation systems, and provide new ideas to create safer, better-quality work. The project addresses the national Australian research and innovation agenda by developing new evidence that may be used in the future to improve worker mental health as well as to reduce health system inefficiencies and inform Australian health decision making through linking different major government public datasets. Additionally, the research aligns with the World Health Organisation's (WHO) Special Initiative for Mental Health (2019-2023) in increasing mental health awareness and reducing mental health stigma and efforts to increase the quality of mental health care through addressing factors within the workplace. Finally, we contribute to the progress of global development goals through research on improving decent work conditions that is good for mental health as illustrated in the United Nations Foundation, Sustainable Development Goals.

We believe the study protocol is appropriate for publication by BMJ Open for the following reasons;

For peer review only

- The project will bring together an interdisciplinary team of researchers from work and organisational psychology, epidemiology, public health, psychiatry and medicine and will use a range of interdisciplinary methods, such as population-based cohort research, data linkage, continuous time structural equational modelling and growth curve modelling to investigate the hidden problem of work stress.
- This research will provide an evidence-based guiding theory that will advocate the need for public policy changes to reduce antidepressant and opioid medication use globally that is linked to corporate climate. This project is timely and significant as it will deepen our understanding of the contribution of work stress to the costs of mental illness (in the form of high antidepressant and opioid use).
- This national project will generate a strong and novel science basis for public policy changes that will yield economic and social benefits to the Australian and International Community**.**
- This research will also contribute to the increasing acknowledgment of the important role mental health plays in achieving global development goals, as indicated by the inclusion of mental health in the Sustainable Developmental Goals, through addressing better working conditions with an emphasis on decent work is good for mental health**.**
- Through the development of a study protocol, we remain true in our position to promote transparency throughout the research process, which is in line with the growing interests of transparency, reproducibility, and reducing publication bias across the wider scientific community.

Previous communication with BMJ Open Journal Staff

Previous communication with staff includes;

- Query in relation to publishing a pre-registration for the current study on the Open Science Framework (OSF) prior to submitting a study protocol to BMJ Open, and whether the protocol would still be accepted by BMJ Open. There was not a definitive answer, therefore a preregistration has not yet been registered on the OSF registries. We would be most appreciative if the Editor could advise if this is appropriate, post submission of the current study protocol.
- Query in relation to data sharing and whether this a mandatory requirement to for protocol publication.
- Query in relation to whether BMJ Open is still accepting study protocols, due to BMJ Open Science ceasing to accept new submissions as of December 2022.

We report the current word count for the study protocol is 4,038 words (this excludes title page, abstract, acknowledgements and references).

We have no conflicts of interest to disclose.

Please address all questions and/or correspondence concerning this protocol to me at Cherie.Crispin@unisa.edu.au

Thank you for your consideration of the study protocol.

Sincerely,

Cherie Crispin
Research Assistant

For peer review only

Authorship:

Submitting & Corresponding Author:

Ms Cherie Crispin
University of South Australia - PSC Global Observatory
Cherie.Crispin@unisa.edu.au
https://orcid.org/0000-0001-6928-8442

Collaborators (Group Authorship)

Ms Cherie Crispin
University of South Australia - PSC Global Observatory
Cherie.Crispin@unisa.edu.au
https://orcid.org/0000-0001-6928-8442

Dr Ali Afsharian
University of South Australia - PSC Global Observatory
Ali.Afsharian@unisa.edu.au
https://orcid.org/0000-0002-8527-6262

Prof Maureen Frances Dollard
University of South Australia - PSC Global Observatory
Maureen.Dollard@unisa.edu.au
https://orcid.org/0000-0002-3128-8727

Prof Dr Christian Dormann
Johannes Gutenberg Universitat Mainz
IsDormann@uni-mainz.de
https://orcid.org/0000-0003-4539-3883

Prof Nicholas Glozier
The University of Sydney – Faculty of Medicine and Health
nick.glozier@sydney.edu.au
https://orcid.org/0000-0002-0476-9146

Dr Tiffany Gill
The University of Adelaide – Adelaide Medical School
tiffany.gill@adelaide.edu.au
https://orcid.org/0000-0002-2822-2436

Prof Anne Taylor
The University of Adelaide – Adelaide Medical School
anne.taylor@adelaide.edu.au
https://orcid.org/0000-0002-4422-7974

Title:

Tranquillizing work stress: Corporate climate and antidepressant and opioid use

Abstract:

Introduction: The mental wellbeing of Australians is a pressing national issue – with one in five Australians experiencing sufficient symptoms of a mental disorder [1] with factors related to employment (and unemployment) the most common risk factors of suicide in the working age population [2]. Recently, Australia has seen a dramatic increase in the use of prescribed antidepressant medications to treat mental health related illnesses [3]. Australia has also seen a dramatic increase in the use of prescribed opioid analgesics for noncancer pain including opioid use for psychological distress and social stressors [4-5]. It is plausible a rise in mental health problems and antidepressant and opioid medication is partly attributable to the corporate climate for worker mental health (i.e., the psychosocial safety climate, PSC). This research aims to identify how PSC and workplace conditions contribute to employee wellbeing and distress that culminate in antidepressant and opioid medication use.

Methods/Analysis: Data will be collected through creative data linkage from the Australian Workplace Barometer [6], to medication data (via the Pharmaceutical Benefits Scheme, PBS). Data will investigate the plausible link between Australia's high levels of antidepressant and opioid use and distress at work. The project advances theory by probing the role corporate climate plays in work design, distress, mental health problems and antidepressant and opioid use. It will determine if antidepressant and opioid use has led to an underestimation of work stress effects. Proposed theoretical models will be analysed through linked data, using continuous time structural equation modelling and growth curve modelling.

Ethics and dissemination:
The study has been approved by the Human Research Ethics Committee of the University of South Australia (Ethics Protocol: 203003). Further, approval from the Australian Institute of Health and Welfare Ethics Committee was also granted for linkage of AWB data and PBS data (EthOS Application EO2022/1/1190).

Results of the study will be disseminated through worldwide keynotes, key international settings, high-impact peer-reviewed journals, industry conference presentations, and media outlets to reach managers, workers and industry partners. Further, UniSA requires publications from public projects to be held in an institutional repository which fulfils the Australian Research Council's Open Access Policy.

Request permissions:

This is an Open Access article distributed in accordance with the Creative Commons Attribution Non-Commercial (CC BY-NC 4.0) license, which permits others to distribute, remix, adapt, build upon this work non-commercially, and license their derivative works on different terms, provided the original work is properly cited and the use is non-commercial. See: http://creativecommons.org/licenses/by-nc/4.0/

If you wish to reuse any or all of this article please use the link below which will take you to the Copyright Clearance Center's RightsLink service. You will be able to get a quick price and instant permission to reuse the content in many different ways.

Data Availability Statement: All data relevant to the study are included in this study protocol. No data sets are available for data sharing.

Registration details: In the interests of transparency, reproducibility, and reducing publication bias across the wider scientific community, the present study has been pre-registered on the Open Science Framework (OSF Registries) DOI: XX XX XX

Article Summary:

- This national project is the first large-scale longitudinal cohort study to investigate the role of corporate climate (i.e., Psychosocial safety Climate), linking work stressors, mental health, mental health problems, and anti-depressant and opioid use in Australia, through creative data linkage.
- This national project will generate a strong and novel science basis for public policy changes that will yield economic and social benefits to the Australian and International Community**.**
- The research will identify which workplace processes can be changed to reduce unnecessary medication costs, which could significantly reduce the burden on Australia's health system and on disability and workers compensation systems, and provide new ideas to create safer, better-quality work.
- Data linkage will overcome common method bias associated with self-report research of mental health, through validation with objective measures.
- Data linkage is a powerful process for combining information about an individual across databases, yet this process also comes with few limitations including, (1) the linkage is an extensive and timely process that includes an intensely rigorous ethics and technical assessment approval process (2) linkage success is dependent on specific individual characteristics being provided, the more information provided the more robust the linkage (i.e. date of birth, first name, middle name, third given name, surname, married name, maternal name, gender, state of residence)

Keywords: Psychosocial safety climate, organisational climate, antidepressant use, opioid use, tranquilising work stress

Introduction/Background:

Work-related stress is a social determinant of global health [7] that represents a huge cost to workers' health and reduces work performance [8]. The mental wellbeing of Australians is a pressing national issue – with one in five Australians experiencing sufficient symptoms of a mental disorder [1] with factors related to employment (and unemployment) the most common risk factors of suicide in the working age population [2]. Since 2017, Australia has seen a dramatic increase in the use of prescribed antidepressant medications to treat mental health related illnesses [3]. The Australian Institute of Health and Welfare (AIHW) reported that in 2020-21, 73.1% of mental health-related prescriptions were filled for anti-depressant medications [3]. Additionally, over the past two decades, Australia has seen a dramatic increase in the use of prescribed opioid analgesics for noncancer pain including opioid use for psychological distress and social stressors [4-5]. According to the AIHW [9], in 2019 opioid use within Australia (3.3% of the adult population) remained higher than the global average (1.2% of the global population aged 15-64 years).

It is plausible a rise in mental health problems and antidepressant and opioid medication is partly attributable to corporate climate. Indeed, only 52% of employees across Australia think their workplace positively supports their mental wellbeing [10], and only 9% of employers have an "integrated and sustained approach to mental health and wellbeing" [11]. Work pressure and

For peer review only

bullying - products of poor management - account for almost 7,500 workers compensation claims per annum for work-related mental disorders [12]. This research aims to identify how corporate climate and workplace conditions contribute to Australian employee wellbeing and distress that culminate in them using antidepressant and opioid medications.

Globally, depression is a major factor in the burden of disease and the leading cause of disability [13]. Yet the theory and evidence linking work to mental ill-health are incomplete in significant ways. Firstly, studies largely focus on work design, such as the levels of job demands (e.g., work pressure, emotional demands such as bullying) and resources (e.g., control, social support, rewards) and their combinations as precursors to worker health and wellbeing. Although there is a strong theoretical (Job Demand-Control-Support - DCS) [14]; and the Effort-Reward Imbalance - ERI – model [15]) and empirical base linking work design to mental health problems [16-18], prominent researchers are now going further and asking “Where do work design and work conditions come from?” [19]. Dollard & Colleagues [20-22] believe that the root cause emanates further upstream, that is with the corporate climate or Psychosocial Safety Climate (PSC).

PSC theory is an innovation in the field [20-22], defined as ‘policies, practices and procedures for the protection of worker psychological health and safety’ [20, p. 579]. It refers to management commitment and priority, organisational communication, and organisational participation and involvement, specifically in relation to worker psychological health and safety. PSC theory has gained prominence nationally and internationally and is a unifying construct that has promoted interdisciplinarity integrating work stress and safety science research. The construct is empirically distinct from related constructs such as team psychological climate, organisational social support, and safety climate [23]. Whereas the safety climate construct is used to predict safety behaviour and accidents and injuries [24], PSC instead is used to predict psychosocial work design and psychological health.

Work design theories are nevertheless important to explain the process and why PSC relates to mental health outcomes. A comprehensive work design framework such as the Job Demands-Resources model [25], is required that integrates the components of ERI and DCS models, encompassing various kinds of demands and resources in explanation of mental health and engagement. Tying PSC to the JD-R model, whereby PSC is the predictor of the JDR model is likely the best explanatory framework and is the backdrop for this study.

This study will investigate the plausible link between distress at work and Australia's high levels of antidepressant and opioid use through the creative linkage of data from the Australian Workplace Barometer (10-year longitudinal study) to antidepressant and opioid medication data (via the national Pharmaceutical Benefits Scheme, PBS). The project advances theory by probing corporate climate's role in work design, distress, mental health problems, stigma/discrimination and antidepressant and opioid use. It will determine if antidepressant and opioid use has led to underestimating work stress effects. Data will also be used to estimate the $AUD cost of work-related antidepressant and opioid use.

The project will yield evidence to stimulate corporate climate change to protect workers' psychological health and well-being. The Australian Workplace Barometer (AWB) has connected work factors to self-reported mental ill-health. However, it has not yet been investigated whether these contribute to the high levels and costs of antidepressant use. Moreover, it is unknown whether the large consumption of antidepressants by Australians is masking a more urgent work stress problem by reducing symptoms leading to underestimation in studies. Uncovering the 'true mental effects' of stressful work is essential.

Research Questions

Research Question 1:

The main research question is ‘Does Psychosocial Safety Climate have a primary (preventing stress from occurring), secondary (coping with stressors as they arise), tertiary (aiming to offset stress symptoms), and quaternary (aiming to offset mental health problems and the need for

For peer review only - http://bmjopen.bmj.com/site/about/guidelines.xhtml

medication) role, in process paths linking work to mental /health problems and antidepressant/opioid medication use?’

Research Question 2:

What is the cost (in $AUD) of antidepressant/opioid medication use related to work factors?

Research Question 3:

Is there a positive relationship between Australian employees’ experience of stressful work demands (e.g. high demands, low resources, bullying, harassment, stigma, discrimination) and antidepressant and opioid medication use.

Research Question 4:

Is there a positive relationship between Australian employees’ experience of distress, emotional exhaustion and depressive symptoms and antidepressant and opioid medication use?

Research Question 5:

Does antidepressant/opioid use lead to an underestimation of the impact of stressful work on employees?

This research will include two separate studies;

Study 1; will look at corporate climate, work stressors, mental health, and *antidepressant* medications

Study 2: will look at corporate climate, work stressors, mental health, and *opioid* medications

Methods & Analysis:

Study Design:

This study will use a longitudinal design that consists of four waves of self-report data using an existing data set, the Australian Workplace Barometer (AWB), a population-based longitudinal data set, matching individuals over time with linkage to objective medication data (Pharmaceutical Benefits Scheme, PBS). The four waves of data from the AWB dataset include data collected in 2009-2010 (Wave 1), 2010-2011 (Wave 2), 2014-2015 (Wave 3) and 2020-2021 (Wave 4). The AWB cohort data will be linked to PBS data collected from 01/01/2009 to the latest available data. The different time points between data collection will help track the progression of effects across different time periods. The longitudinal study will be on matched individuals over time with linkage to objective medication data (Pharmaceutical Benefits Scheme, PBS).

Explanation of existing data

As of the submission date, the data exists, and researchers have only had access to the AWB data. However, no detailed analysis has been conducted concerning the current research plan (including the calculation of summary statistics). Access to the AWB data has been limited, though a small portion of the data has been used to conduct exploratory analysis that showed encouraging initial results. PBS data has not been accessed at the point of registration.

Data collection procedures

The Australian Workplace Barometer (AWB) used a population-based random sampling approach to conduct computer-assisted telephone interviews (CATI). Participants were recruited randomly from the Australian electronic white pages inviting household members to participate (the participant had to have had the last birthday in the household to be eligible).

For peer review only - http://bmjopen.bmj.com/site/about/guidelines.xhtml

For each subsequent wave of data collection, those who had consented to be re-contacted were sent a letter advising of further data collection and re-contact for a further interview. A combination of modes was used to collect subsequent waves of AWB data to increase sample representativeness and participation uptake, including telephone interviews by landline or mobile, surveys via email or SMS link, and hard copy surveys sent via post (including a postage-paid self-addressed envelope for returning the survey). Where participants could not partake in interviews at the first point of contact, call-backs for a more suitable time were arranged and scheduled with the research team accordingly.

Telephone interviews were conducted by a team of research assistants trained to enter data immediately into the Qualtrics online survey. Participants who received an email or SMS link were provided with their participant ID (required for linkage) and entered their participant ID and self-reported data directly into the Qualtrics survey link provided. Hard copy surveys were entered manually into the Qualtrics online survey by the research team upon receipt.

Payment for Participation:

In the fourth wave of data collection, participants who completed the survey automatically went into the draw to receive a gift card valued at $100. The winner of the draw was picked using a random number generator, contacted by the research team's chief investigator by phone, and announced the winner.

Inclusion criteria:

Participants are 18-65 years of age and currently in paid employment. Sub-contractors must have worked with the same employer for a minimum of 4 months. Participants must have consented to their personal AWB information being securely sent to the Australian Institute of Health and Welfare (AIHW) to match the Pharmaceutical Benefits Scheme (PBS) dataset for research purposes, to investigate how work conditions relate to mental health and relate medication use.

Exclusion criteria:

Ages out of the age range, unemployed, and those who did not consent to data linkage are excluded from the dataset.

Pharmaceutical Benefits Scheme (PBS) Dataset:

National Medicare & PBS will provide data on prescription medicines, defined by the Australian Institute of Health and Welfare (AIHW) project as antidepressants to treat clinical depression and opioid medication use for this project. The Pharmaceutical Benefits Scheme (PBS) data comprises individual antidepressant and opioid medication use (summed over a year) from 2009 to 2021.

Data Linkage (AWB linkage to PBS):

PBS data was linked via AWB participants who consented to have their AWB data linked to PBS data in 2020/2021. Antidepressant medication will be matched with the AWB at the individual level, using individual names and addresses.

Data Linkage Procedure:

Step 1: A 'Request for linkage of national data: technical assessment' was completed by Dr Ali Afsharian (University of South Australia) as part of the AIHW data linkage protocol and AIHW ethics approval, which outlined the proposed research and study aims.

Step 2: AIHW was sent two files via secure email, including:

- AWB cohort personal identifiers with a data ID code
- AWB content data and with the data ID code

Step 3: AIHW will link the AWB cohort to PBS. This linkage process includes the following;

- Creation of a new project-specific person number

- Loading the PBS content data and the AWB content data to the SURE project-specific person number
- All data transfers will be via secure messenger service.

Important Data Linkage Notes:

- The technical assessment is a rigorous process required by AIHW to ensure that the data requested for the linkage answer the proposed research questions. The technical assessment ensures the study aims are achievable using the proposed linkage methodology and forms part of the AIHW ethics approval for the research project.
- AIHW set up a specific project ID for the linkage of the AWB and PBS data and the provision of a secure workspace, referred to as the Secure Unified Research Environment (SURE) workspace, for the analysis of linked data. All analytical software packages (SPSS, R) are loaded onto the SURE secure workspace. Deidentified AWB data set and PBS data are curated into the secure workspace.
- There are strict protocols in place during SURE access, where the original AWB dataset is held on a secure university server that restricts researcher access involved with SURE data analysis. This highly confidential workspace is constructed to hold data that blocks all access to other desktop files and software to avoid identifying deidentified participants.
- A research Assistant, Ms Cherie Crispin, will be the data custodian (not involved in the data analysis) and will be appointed during this time to ensure no local data access of identifiable/ re-identifiable data while there is access to SURE data. All data analysis takes place with the data on the secure SURE website.

Sample size

The study sample is part of a more extensive population-based study, the Australian Workplace Barometer (AWB) project, which has multiple waves of data collected from over 9,000 working Australians between 2009 and 2020/2021. For the present study, we used data collected in 2009, 2010/2011, 2014/2015 and 2020/2021. The sample included 1372 working Australians who participated in the AWB study from 2009 to 2021 and consented to the data linkage. Participants are located across all Australian states and territories; South Australia (SA), New South Wales (NSW), Western Australia (WA), Tasmania, Australian Capital Territory (ACT), and the Northern Territory (NT).

Measured variables

Demographics:

Demographic variables in the current study include age, gender, industry, education and income.

Psychosocial Safety Climate:

The psychosocial safety climate was measured using a scale consisting of 12 items (PSC-12) [26] that assess four dimensions of PSC, including management priority, management commitment, organizational communication, and organizational participation. Items were measured on a 5-point Likert scale, ranging from 1 (strongly disagree) to 5 (strongly agree). Scores for all 12 items are summed to provide an overall score for PSC. An example item is, “In my workplace, senior management acts quickly to correct problems/issues that affect employees’ psychological health”.

Psychosocial Demands:

1. Work pressure

Work pressure was measured using the 5-item psychological job demands scale from the Job Content Questionnaire (JCQ 2.0) [27], an example item “My job requires working very fast”. Items were measured on a 4-point Likert scale, ranging from 1 (strongly disagree) to 4 (strongly agree). The five items were summed to give a total score for the work pressure.

2. Workplace bullying:

Bullying was assessed using an amended version of the QPSNordic Bullying Questionnaire [28]. Participants were provided with a definition of workplace bullying and then answered questions about their exposure to bullying in the workplace.

Definition provided to participants; “Bullying is a problem at some workplaces and for some workers. To label something, as bullying, the offensive behaviour has to occur repeatedly over a period of time, and the person confronted has to experience difficulties defending him or herself. The behaviour is not bullying if two parties of approximate equal ‘strength’ are in conflict or the incident is an isolated event” [29].

Participants were then asked, “Have you been subjected to bullying at the workplace during the last six months?” Those who replied with ‘yes’, were then asked by whom (supervisor/co-worker) and the length and frequency of exposure to workplace bullying.

3. Workplace harassment

Seven items from Richman et al.’s (1996) scale [30] were used to measure workplace harassment. Items were measured on a 5-point Likert scale, ranging from 1 (very rarely/never) to 5 (very/often always) and summed together to represent a total for workplace harassment. An example item is “I have been sworn at and/or yelled at”.

Psychosocial demands (work pressure, workplace bullying & workplace harassment) are independent variables, all measured longitudinally at the individual level.

Psychosocial Resources

1. Job control

Three subscales were used from the JCQ 2.0 [27] to measure job control, including the 5-item skill discretion subscale ("My job requires that I learn new things"), the 3-item decision authority subscale ("In my job I have very little freedom to decide how I do my work") and the 3-item macro-decision latitude subscale ("In my organization, I have significant influence over decisions made by my work team and department"). All 12 items were measured on a 4-point Likert scale, ranging from 1 (strongly disagree) to 4 (strongly agree). Items were summed to provide a total for job control.

2. Social Support

To measure social support, six items from the JCQ 2.0 [27] was used, three items for supervisor social support ("My supervisor/manager is helpful in getting the job done") and three items for co-worker support ("People I work with are friendly"). Items were measured on a 4-point Likert scale, ranging from 1 (strongly disagree) to 4 (strongly agree) and summed for both supervisor and co-worker to provide a total score for each social support scale.

Job control (skill discretion, decision authority & macro-decision latitude) and social support (supervisor and co-worker) are independent variables, measured longitudinally at the individual level.

Distress Symptoms

1. Psychological distress

The Kessler 10 scale [31] was used to measure psychological distress. A 5-point Likert scale measured responses, ranging from 1 (none of the time) to 5 (all of the time). Scores on the ten items were totalled to give an overall score on psychological distress. An example item is "[In the past four weeks], about how often did you feel tired out for no good reason?".

2. Emotional exhaustion

Five items from the Maslach Burnout Inventory [32] were used to measure emotional exhaustion on a 7-point Likert scale, ranging from 1 (never) to 7 (every day). An example item is, "I feel burned out from my work."

Distress symptoms &/or mental health problems are dependent variables measured longitudinally (four-time points) using psychological distress and emotional exhaustion at the individual level.

Work Engagement

Work engagement was measured using three items from the Utrecht Work Engagement Scale – Shortened Version (UWES-9) [33], covering vigour, dedication and absorption. Items were measured on a 7-point Likert scale, ranging from 1 (never) to 7 (every day) and summed together to provide an overall score for work engagement. An example item is "At work I feel bursting with energy".

Engagement will be used as a dependent variable, measured longitudinally at the individual level.

Stigma/Discrimination

Stigma/discrimination was measured using one item from the Kessler 6 (K6) mental health symptom screening questionnaire ("In the last 12 months, do you think you have had emotional or mental health problems?"). If participants answered 'yes', a further seven questions were asked about their experiences within the workplace of discrimination ("Have any of your supervisors and or managers treated you unfairly or discriminated against you because of the mental health problems?"), avoidance ("Have any of your supervisors and or managers avoided you because of the emotional or mental health problems?") and positive treatment ("Because of your mental health or emotional problems have you been treated more positively by your employer?") [34].

Stigma/discrimination was only measured in wave 4 (2020/2021) at the individual level. Stigma/discrimination will be used as a moderator (third variable) in the present study.

Mental Health Problems

1. Depression

All nine items from the Patient Health Questionnaire (PHQ-9) [35] were used to measure depressive symptoms in the present study, with a time reference modified for the last four weeks, an example item "[During the last month, how often were you bothered by] feeling down, depressed, or hopeless?". Items were measured on a 4-point Likert scale, from 1 (not all) to 4 (nearly every day). Scores were totalled to give an overall score on depression.

2. Suicidal ideation

One item from the Patient Health Questionnaire (PHQ-9) [35] was used to measure suicidal ideation ("[During the last month, how often were you bothered by] thoughts that you would be better off dead or thoughts of hurting yourself in some way?").

Depression and suicidal ideation are dependent variables, measured longitudinally (four-time points) at the individual level.

Prescribed Medication Usage

1. Self-report prescribed medication usage data

Participants were asked the question, "Have you taken any prescription medications for your mental health in the last 12 months?". Those who answered 'yes' were then asked to indicate from a list of prescribed medications (sleeping tablets/capsules, tablets/capsules for anxiety or nerves, tranquillizers, antidepressants, mood stabilizers, and other medications for your mental health) which medications they had used in the last 12 months, and also in the last four weeks.

Antidepressant medication is a dependent variable measured only in wave 4 (2020/2021) at the individual level.

2. Registered PBS Medication Data

PBS variables include PBS item, drug type, Benefit amount, the Patient contribution amount, under co-payment prescription type, prescribed speciality, prescriber type, date of prescribing, number of scripts dispensed, quantity supplied, date of supply, and patient category.

Data Analysis Plan:

Confirmatory Analysis Plan

The proposed theoretical models will be analysed using data linked from the AWB and AIHW, continuous time structural equation modelling (SEM), hierarchical linear modelling to test relationships, and growth curve modelling. To this end, SPSS and R software will be applied. The study will look at correlations between PSC, work design factors and antidepressant and opioid medication use while controlling for a range of confounders such as gender, age, industry, education and SES (income and work post-codes will be assembled into Statistical Areas/ SEIFA index). The study will investigate differences by these demographic classifications, and confirm the association between work factors, stress reactions, and antidepressant and opioid medication use. Specifically, we will look at the relationship between psychosocial demands and antidepressant and opioid use, where psychosocial demands is expected to correlate positively with antidepressant and opioid use.

Additionally, we will investigate the association between employees' experience of distress and antidepressant and opioid medication use, where distress symptoms are expected to correlate positively with antidepressant and opioid medication usage. Finally, we will investigate the relationship between antidepressant and opioid use and an underestimation of stressful work by Australian employees.

We may control for baseline levels of antidepressant and opioid medication use to further account for stable influences on use (such as personal factors) while assessing predictive relationships. Even though antidepressant and opioid use may generally be high, we seek to examine variations in the use of antidepressants and opioids across time that can be linked to work conditions. If there is a relationship, we may be able to conclude that working conditions are contributing to the high levels of antidepressant and opioid use.

Bayesian analysis will be applied to estimate random intercepts and slopes for individuals to understand future time points for which the individual may be at risk for suicidal ideation.

Transformations

Data transformation for some scales like PSC-12 will be applied to see the antidepressant and opioid use trends with different categories of low to high-risk PSC.

Data exclusion

Unemployed subjects (i.e., retired since the previous time of data collection), self-employed and sub-contractors working with the same employer for less than 4 months, will be excluded. For adequate data analysis, outliers will be included in further data analysis.

Missing data

If a subject does not complete items for each scale, that subject will not be included in the analysis. In cases, with more than 60% of scale completion, we may use a mean replacement strategy to aggregate the data into higher team or group levels.

Exploratory Analysis Plan

We will investigate the role of stigma/discrimination (moderator) and PSC (predictor/mediator) for workers experiencing work stress/distress symptoms and their association with anti-depressant and opioid medication use to determine whether the corporate climate tranquillizes the effects of work stress and anti-depressant and opioid use and whether stigma moderates these effects. We will explore mental health vulnerability as a predictor by assessing a range of indicative states suggested by mental health self-reports. Instead of controlling for this as prior research [36], we will investigate its trajectory, subsequent stressor exposure (e.g., bullying), and future mental health status. This has implications for how mental health is managed in the workplace. We will also investigate the validity of self-report medication use data against PBS data (registered data).

Ethics & Dissemination:

The study has been reviewed and approved by the Human Research Ethics Committee of the University of South Australia (Ethics Protocol: 203003). Further, approval from the Australian Institute of Health and Welfare Ethics Committee was also granted for the linkage of AWB data and PBS data (EthOS Application EO2022/1/1190).

Results of the study will be disseminated through worldwide keynotes, key international settings such as the Joint Congress of ICOH-WOPS & APA-PFAW (2023), high-impact peer-reviewed journals, industry conference presentations, and various media outlets (such as LinkedIn, Twitter, radio, community forums) to reach managers, workers and industry partners. Further, UniSA requires publications from public projects to be held in an institutional repository which fulfils the Australian Research Council's Open Access Policy. Therefore, we will also lodge relevant outputs with the Analysis and Policy Observatory collection for greater accessibility.

Author Contributions:

Cherie Crispin : Ms Crispin was involved in collection of longitudinal data since 2020. She will assist with preparation and drafting of study papers, ethics applications, development of study protocol, development of model design and theoretical framework for Study Two (corporate climate, work stressors, mental health and *opioid* medications), provision of AWB data sets for data linkage agencies, liaison with data linkage agencies

Dr Ali Afsharian : Dr Afsharian was involved in collection of longitudinal data since 2020. He will assist with ethics applications, progress reports, drafting papers and study protocol, development of methodology, model design and theoretical framework for Study One (corporate climate, work stressors, mental health, and *antidepressant* medications), liaise with data linkage agencies and will be one of two researcher team members to perform computations and statistical analysis.

Professor Maureen Dollard : Prof Dollard conceived of the presented idea, developed and contributed to the original design and implementation of the research. Prof Dollard will lead, supervise and coordinate the research team and take overall responsibility for the strategic intellectual direction of the project and communication of results. Further, she will assist with theory development, methodology and model design for Studies One and Two and will be one of two research team members to perform computations and statistical analysis.

Professor Christian Dormann : Prof Dormann will assist the team in integrating and testing new societal cost elements of Psychosocial Safety Climate (PSC) theory using R and structural equational modelling and contribute to publication of results.

Dr Nick Glozier : Dr Glozier will be responsible for aspects of data linkage, mental health and health service evaluation, and PBS medication interpretation and will contribute to the costing and epidemiological aspects of the study design.

For peer review only

Dr Tiffany Gill : Dr Gill is an epidemiologist who was involved in the original development and implementation of the AWB surveys in 2009. She will provide advice on survey, ethical and other project related issues as required.

Professor Anne Taylor : Prof Taylor will assist with expert advice on interpretation of data analysis and data-linkage. Prof Taylor was originally involved with sampling, the set-up of Computer Assisted Telephone Interviewing (CATI) and data collection. She will assist with the review of papers where required.

All authors have reviewed and approved the final version of the study protocol.

Funding Statement:

This work was supported by five grants: (1) Australian Research Council (ARC) DP200102752 (103319) Dollard M.F. (2) DP087900 Australian Research Council Discovery Grant. Dollard MF, Winefield AH, LaMontagne AD, Taylor AW, Bakker AB, Mustard C. Working wounded or engaged? Australian work conditions and consequences through the lens of the job demands resources model. (3) LP100100449 Australian Research Council Linkage Grant. Dollard MF, Winefield AH, Taylor AW, Smith PM, Nafalski A, Bakker A, Dormann C. State, organisational, and team interventions to build a psychosocial safety climate using the Australian Workplace Barometer and the StressCafé. (4) FL200100025 Australian Research Council Laureate Fellowship. (5) Safework NSW's Centre for WHS.

Competing interests' statement.

None declared

References:

1. Australian Bureau of Statistics. National study of mental health and wellbeing [online] 2022. https://www.abs.gov.au/statistics/health/mental-health/national-study-mental-health-and-wellbeing/latest-release (accessed 28 February 2023).
2. Australian Bureau of Statistics. Causes of death, Australia [online]. 2022. https://www.abs.gov.au/statistics/health/causes-death/causes-death-australia/latest-release (accessed 28 February 2023).
3. Australian Institute of Health and Welfare. Mental Health Prescriptions [online]. 2022. https://www.aihw.gov.au/mental-health/topic-areas/mental-health-prescriptions (accessed 28 February 2023).
4. Lalic, S., Ilomaki, J., Bell, J. S., et al. (2019). Prevalence and incidence of prescription opioid analgesic use in Australia. British Journal of Clinical Pharmacology, 85, 202-215. https://doi.org/10.1111/bcp.13792
5. Wilson, J., Lam, T., Scott., D., et al. (2020). 'Extreme personal stress' and 'a lot of pain': Exploring the physical, mental and social contexts of extramedical pharmaceutical opioid use in Australia paramedic case descriptions. Drug and Alcohol Review, *39,* 870-878. https://doi.org/10.1111/dar.13118
6. Dollard, M. F. & Bailey, T. (2014). The Australian workplace barometer: Psychosocial safety climate and working conditions in Australia. Samford Valley, QLD: Australian Academic Press Group Pty Ltd.
7. World Health Organisation (WHO). Closing the gap in a generation: Health equity through action on the social determinants of health [online]. 2008. https://www.who.int/publications/i/item/WHO-IER-CSDH-08.1 (accessed on 14 March 2023).
8. International Labour Organisation (ILO). World Employment and Social Outlook: Trends 2016 [online]. 2016. https://www.ilo.org/wcmsp5/groups/public/@dgreports/@dcomm/@publ/documents/publication/wcms_443480.pdf (accessed on 14 March 2023).

9. Australian Institute of Health and Welfare. Opioid harm in Australia; and comparisons between Australia and Canada [online]. 2018. https://www.aihw.gov.au/getmedia/605a6cf8-6e53-488e-ac6e-925e9086df33/aihw-hse-210.pdf.aspx?inline=true (accessed on 28 February 2023).
10. beyondblue. The State of Workplace Mental Health in Australia [online]. 2015. https://www.headsup.org.au/docs (accessed on 28 February 2023).
11. Safe Work NSW. Mentally healthy workplaces in NSW [online]. 2017. https://www.safework.nsw.gov.au/__data/assets/pdf_file/0018/320139/Mentally-healthy-workplaces-in-NSW-benchmarking-tool-October-2017-SW08734.pdf (accessed on 14 March 2023).
12. Safe Work Australia. Compendium of Workers' Compensation Statistics Australia 2015-16 [online]. 2017b. https://www.safeworkaustralia.gov.au (accessed on 14 March 2023).
13. World Health Organisation. Depression [online]. 2021. https://www.who.int/news-room/fact-sheets/detail/depression (accessed on 28 February 2023).
14. Karasek, R. & Theorell, T. (1990). Healthy Work. New York: Basic Books
15. Siegrist, J (1996). Adverse health effects of high-efforts/low-reward conditions. Journal of Occupational Health Psychology,1, 27–41. https://doi.org/10.1037//1076-8998.1.1.27
16. Leach, LS., Poyser, C. & Butterworth. (2016). Workplace bullying and the association with suicidal ideation/thoughts and behaviour: a systematic review. Occupational and Environmental Medicine, 74(1). https://doi.org/10.1136/oemed-2016-103726
17. Loerbroks, A., Cho, S., Dollard, M.F., et al. (2016). Associations between work stress and suicidal ideation. Journal of Psychosomatic Research, 90, 62–69. https://doi.org/10.1016/j.jpsychores.2016.09.008
18. Theorell, T., Hammarstrom, A., Aronsson, G., et al. (2015). A systematic review/ meta-analysis of work environment and depressive symptoms. BMC Public Health, 15, 738. https://doi.org/10.1186/s12889-015-1954-4
19. Parker, SK, Morgeson, FP, & Johns, G. (2017). One hundred years of work design research. Journal of Applied Psychology, 102(3), 403. https://doi.org/10.1037/apl0000106
20. Dollard, MF & Bakker, AB. (2010). Psychosocial safety climate as a precursor to conducive work environments, psychological health problems, and employee engagement. Journal of Occupational and Organizational Psychology, 83, 579–599. https://doi.org/10.1348/096317909X470690
21. Dollard, MF & Karasek, R. (2010). Building psychosocial safety climate: Evaluation of a socially coordinated PAR risk management stress prevention. In J Houdmont & S Leka (Eds), Contemporary occupational health psychology (p. 208–234). Chichester: Wiley Blackwell. https://doi.org/10.1002/9780470661550.ch11
22. Law, R, Dollard, M.F., Tuckey, M.R., et al. (2011). Psychosocial safety climate as a lead indicator of workplace bullying and harassment, job resources, psychological health and employee engagement. Accident Analysis and Prevention, 43(5), 1782–1793. https://doi.org/10.1016/j.aap.2011.04.010
23. Idris, MA, Dollard, MF, Coward, J., et al. (2012). Psychosocial safety climate: conceptual distinctiveness and effect on job demands and worker psychological health. Safety Science, 50, 19–28. https://doi.org/10.1016/j.ssci.2011.06.005
24. Demerouti, E., Bakker, A., B., Nachreiner, F., et al. (2001). The job demands-resources model of burnout. Journal of Applied Psychology, 86, 499–512. PMID: 11419809
25. Griffin, M, & Curcuruto, M. (2016). Safety Climate in Organizations. Annual Review of Organizational Psychology and Organizational Behavior, 3(1). https://doi.org/10.1146/annurev-orgpsych-041015-062414
26. Hall, G. B., Dollard, M. F., Coward, J. (2010). Psychosocial Safety Climate: Development of the PSC-12. International Journal of Stress Management, 4, 353–383. https://doi.org/10.1037/a0021320
27. Karasek, R., Brisson, C., Kawakami, N., et al. (1998). The Job Content Questionnaire (JCQ): an instrument for internationally comparative assessments of psychosocial job

For peer review only

characteristics. Journal of Occupational Health Psychology, 3(4), 322–355. https://doi.org/10.1037//1076-8998.3.4.322

28. Elo A.-L., Dallner M., Gamberale F., et al. (2000). Validation of the Nordic Questionnaire for Psychological and Social Factors at Work—QPSNordic. In M. Vartiainen, F. Avallone, & N. Anderson (Eds.), *Innovative theories, tools, and practices in work and organizational psychology* (pp. 47–57). Hogrefe & Huber Publishers.
29. Ørhede, E., Hottinen, V., Skogstad, A., et al. (2000). User's Guide for QPSNordic. General Nordic questionnaire for psychological and social factors at work. Copenhagen: Nordic Council of Minister.
30. Richman, J. A., Flaherty, J. A., & Rospenda, K. M. (1996). Perceived workplace harassment experiences and problem drinking among physicians: Broadening the stress/alienation paradigm. Addiction, 91(3), 391–403. https://doi.org/10.1046/J.1360-0443.1996.9133918.X
31. Kessler, R. & Mroczek, D. (1994). Final Version of our Non-specific Psychological Distress Scale. Ann Arbort (MI): Survey Research Center of the Institute for Social Research, University of Michigan.
32. Schaufeli, W., Leiter, M., Maslach, C. et al. (1996) Maslach Burnout Inventory-General Survey. In: Maslach, C., Jackson, S.E. and Leiter, M.P., Eds., The Maslach Burnout Inventory: Test Manual, Consulting Psychologists Press, Palo Alto, CA.
33. Schaufeli, W. B., Bakker, A. B., & Salanova, M. (2006). The Measurement of Work Engagement With a Short Questionnaire: A Cross-National Study. *Educational and Psychological Measurement, 66*(4), 701–716. https://doi.org/10.1177/0013164405282471
34. Reavley, N. J., Morgan, A. J. & Jorm, A. F. (2016). Disclosure of mental health problems: findings from an Australian national survey. Epidemiology and Psychiatric Sciences, 1-11. https://doi.org/10.1017/S204579601600113X
35. Spitzer, R L., Kroenke, K. & Williams, J. B. (1999). Validation and utility of a self-report version of PRIME-MD: The PHQ primary care study. JAMA. https://doi.org.10.1001/jamma.282.18.1737
36. Virtanen, M., Honkonen, T., Kivimaki, M. et al. (2007). Work stress, mental health and antidepressant medication findings from the Health 2000 Study. Journal of Affective Disorders, 98, 189–97. https://doi.org/10.1016/j.jad.2006.05.034

For peer review only

BMJ Open

Psychosocial safety climate (PSC) and working conditions, predictors of mental health and antidepressant and opioid use: A study protocol for longitudinal data linkage.

Journal:	*BMJ Open*
Manuscript ID	bmjopen-2023-074235.R1
Article Type:	Protocol
Date Submitted by the Author:	14-Nov-2023
Complete List of Authors:	Crispin, Cherie; University of South Australia, Justice & Society<br>Afsharian, Ali; University of South Australia, Justice & Society<br>Loh, May; University of South Australia, Justice & Society<br>Dollard, Maureen F.; University of South Australia,<br>Dormann, Christian; Johannes Gutenberg Universitat Mainz<br>Glozier, Nick; University of Sydney Brain and Mind Research Institute,<br>Gill, Tiffany; The University of Adelaide, Medical Specialities<br>Taylor, Anne; The University of Adelaide, School of Medicine
**Primary Subject Heading**:	Mental health
Secondary Subject Heading:	Epidemiology, Occupational and environmental medicine, Public health, Research methods
Keywords:	OCCUPATIONAL & INDUSTRIAL MEDICINE, EPIDEMIOLOGY, Health & safety < HEALTH SERVICES ADMINISTRATION & MANAGEMENT, Health policy < HEALTH SERVICES ADMINISTRATION & MANAGEMENT, MENTAL HEALTH, PUBLIC HEALTH

SCHOLARONE™
Manuscripts

Title:

Psychosocial safety climate (PSC) and working conditions, predictors of mental health and antidepressant and opioid use: A study protocol for longitudinal data linkage.

Abstract:

Introduction: Work-related stress is a social determinant of global health [1] that represents a huge cost to workers' health and reduces work performance [2]. In Australia mental wellbeing is a pressing national issue – with one in five Australians experiencing mental disorders [4]. Anti-depressants are a first-line medication commonly used to treat mental disorders [6]. Recently, Australia has seen a dramatic increase in the use of prescribed antidepressant medications to treat mental health related illnesses [8]. Australia has also seen a dramatic increase in the use of prescribed opioid analgesics for noncancer pain including opioid use for psychological distress and social stressors [9-10]. It is plausible a rise in mental health problems and antidepressant and opioid medication is partly attributable to the corporate climate for worker mental health (i.e., the psychosocial safety climate, PSC). This research aims to identify how PSC and workplace conditions contribute to employee wellbeing and distress that culminate in antidepressant and opioid medication use.

Methods/Analysis: Data will be collected through creative data linkage from the Australian Workplace Barometer (AWB) [30], to medication data (via the Pharmaceutical Benefits Scheme, PBS). The participant sample will include 1,372 working Australians from the AWB project from 2009 to 2021. Four waves of longitudinal data from 2009 to 2021 will be used to investigate the plausible link between Australia's high levels of antidepressant and opioid use and distress at work. The project advances theory by probing the role corporate climate plays in work design, distress, mental health problems and antidepressant and opioid use. It will determine if antidepressant and opioid use has led to an underestimation of work stress effects. Proposed theoretical models will be analysed through linked data, using continuous time structural equation modelling (SEM), hierarchical linear modelling (HLM), logistic regression and cost estimation.

Ethics and dissemination:
The study has been approved by the Human Research Ethics Committee of the University of South Australia (Ethics Protocol: 203003). Further, approval from the Australian Institute of Health and Welfare Ethics Committee was also granted for linkage of AWB data and PBS data (EthOS Application EO2022/1/1190).

Results of the study will be disseminated through worldwide keynotes, key international settings, high-impact peer-reviewed journals, industry conference presentations, and media outlets to reach managers, workers and industry partners. Further, UniSA requires publications from public projects to be held in an institutional repository which fulfils the Australian Research Council's Open Access Policy.

Strengths and Limitations:

- This study is the first large-scale longitudinal data linkage to investigate the role of PSC in linking work stressors, mental health, and medication use in the Australian working population.
- The results will help to reveal the true burden of mental health associated with workplace conditions in Australia, that may have otherwise been masked by the effects of antidepressant and opioid medication use.

For peer review only

- The results will provide an evidence-based guiding theory to drive public policy change to reduce antidepressant and opioid medication use globally, that is directly linked to the workplace corporate climate (i.e. PSC).
- Data linkage will overcome common method bias associated with self-report research of mental health, through validation with objective measures.
- Data linkage success is largely dependent on specific individual participant characteristics (e.g. first name, surname, date of birth) being provided at the point of interview. As this information was limited this restricted a more robust data linkage.

Request permissions:

This is an Open Access article distributed in accordance with the Creative Commons Attribution Non-Commercial (CC BY-NC 4.0) license, which permits others to distribute, remix, adapt, build upon this work non-commercially, and license their derivative works on different terms, provided the original work is properly cited and the use is non-commercial. See: http://creativecommons.org/licenses/by-nc/4.0/
If you wish to reuse any or all of this article please use the link below which will take you to the Copyright Clearance Center's RightsLink service. You will be able to get a quick price and instant permission to reuse the content in many different ways.

Data Availability Statement: All data relevant to the study are included in this study protocol. The Australian Workplace Barometer Project Dataset was collected from 2009 to 2015 (Wave 1-3 inclusive) and is available through the Australian National University Library. Pharmaceutical Benefit Scheme data is not available for sharing.

Registration details: In the interests of transparency, reproducibility, and reducing publication bias across the wider scientific community, the present study has been pre-registered on the Open Science Framework (OSF Registries) DOI: XX XX XX

Keywords: Psychosocial safety climate, organisational climate, antidepressant use, opioid use, work stress, mental health

Background:

Work-related stress is a social determinant of global health [1] that represents a huge cost to workers' health and reduces work performance [2]. Every year an estimated 12 billion working days are lost to depression and anxiety, with approximately 15% of working-age adults worldwide reported to have a mental disorder in 2019 [3]. In Australia alone, one in five Australians are experiencing mental disorders [4] with problems related to employment the most common risk factors of suicide in the working age population [5].
A common form of treatment for mental disorders is antidepressant medication [6]. Epidemiological studies have demonstrated an increase in usage of antidepressant prescriptions in industrialised countries during the 21st century [6,7]. Since 2017, Australia has seen a dramatic increase in the use of prescribed antidepressant medications to treat mental health related illnesses [8]. The Australian Institute of Health and Welfare (AIHW) reported that in 2020-21, 73.1% of mental health-related prescriptions were filled for anti-depressant medications [8]. Additionally, over the past two decades, Australia has seen a dramatic increase in the use of prescribed opioid analgesics for noncancer pain including opioid use for psychological distress and social stressors [9-10]. According to the AIHW [11], in 2019 opioid use within Australia (3.3% of the adult population) remained higher than the global average (1.2% of the global population aged 15-64 years). It is plausible a rise in mental health problems and antidepressant and opioid medication is partly attributable to the corporate climate. Indeed, only 52% of employees across Australia think their workplace positively supports their mental wellbeing [12], and only 9% of employers have an "integrated and sustained approach to mental health and wellbeing" [13]. Work pressure and bullying - products of poor management - account for almost 7,500 workers compensation claims per annum for work-related mental disorders [14].

For peer review only

During the past few decades, a multitude of studies have been published on working conditions and mental health and prescribed medication use [15-17]. Yet the theory and evidence linking work to mental ill-health and medication use are incomplete in significant ways. Firstly, studies largely focus on work design, such as the levels of job demands (e.g., work pressure, emotional demands such as bullying) and resources (e.g., job control, social support) and their combinations as precursors to worker ill health and increased risk of depressive symptoms [16]. Although there is a strong theoretical (Job Demand-Control-Support - DCS) [18]; and the Effort-Reward Imbalance - ERI – model [19]) and empirical base linking work design to mental health problems [16, 20-21], prominent researchers are now going further and asking, “Where do work design and work conditions come from?” [22]. Dollard & colleagues [23-25] believe that the root cause emanates further upstream, that is with the corporate climate or Psychosocial Safety Climate (PSC).

PSC theory is an innovation in the field [23-25], defined as ‘policies, practices and procedures for the protection of worker psychological health and safety’ [23, p. 579]. It refers to management commitment and priority, organisational communication, and organisational participation and involvement, specifically in relation to worker psychological health and safety. PSC theory has gained prominence nationally and internationally and is a unifying construct that has promoted interdisciplinarity integrating work stress and safety science research. The construct is empirically distinct from related constructs such as team psychological safety, perceived organisational support, and safety climate [26]. Whereas the safety climate construct is used to predict safety behaviour and accidents and injuries [27], PSC instead is used to predict psychosocial work design and psychological health and is also linked to the enactment of safety behaviours and the reporting of workplace injuries [28] .

Work design theories are nevertheless important to explain the process and why PSC relates to mental health outcomes. A comprehensive work design framework such as the Job Demands-Resources model [29], is required that integrates the components of ERI and DCS models, by encompassing various kinds of demands and resources in explanation of mental health and engagement. Work design is influenced by the organisational priorities and policies. T tying PSC to work design theories (e.g., the JD-R model), whereby PSC is the predictor of the JDR model) is likely a feasible explanatory framework to understand the relationship between work conditions associated with work stress reactions and mental health problems linked to antidepressant and opioid medication use. The PSC extended JDR theory is the backdrop for this study.

This study will yield evidence to stimulate corporate climate change to protect workers' psychological health and well-being. The Australian Workplace Barometer (AWB) has connected work factors to self-reported mental ill-health [30]. However, it has not yet been investigated whether these contribute to the high levels and costs of antidepressant use. Moreover, it is unknown whether the large consumption of antidepressants by Australians is masking a more urgent work stress problem by reducing symptoms leading to underestimation in studies. Uncovering the 'true mental effects' of stressful work is essential.

Aims

This research aims to identify how the corporate climate and workplace conditions contribute to Australian employee wellbeing and distress that culminate in them using antidepressant and opioid medications. This study will investigate the plausible link between distress at work and Australia's high levels of antidepressant and opioid use through the creative linkage of data from the Australian Workplace Barometer (10-year longitudinal study) to antidepressant and opioid medication data (via the data collected by Australian national Pharmaceutical Benefits Scheme (which contains information on prescribed medicines that qualify for medical claims). The project advances theory by probing corporate climate's role in work design, distress, mental health problems, stigma/discrimination and antidepressant and opioid use. It will determine if

For peer review only - http://bmjopen.bmj.com/site/about/guidelines.xhtml

antidepressant and opioid use has led to underestimating work stress effects. Data will also be used to estimate the $AUD cost of work-related antidepressant and opioid use.

Research Questions

Research Question 1:

The main research question is 'Does Psychosocial Safety Climate have a primary role, in process pathways linking working conditions to mental health problems and antidepressant/opioid medication use?'

Research Question 2:

What is the cost (in $AUD) of antidepressant/opioid medication use related to work factors?

Research Question 3:

Is there a positive relationship between Australian employees' experience of stressful work conditions (e.g., high demands, low resources, bullying, harassment, stigma, discrimination) and antidepressant and opioid medication use.

Research Question 4:

Is there a positive relationship between Australian employees' experience of distress, emotional exhaustion and depressive symptoms and antidepressant and opioid medication use?

Research Question 5:

Does antidepressant/opioid use lead to an underestimation of the impact of stressful work on employees?

This research will include two separate studies;

Study 1; will look at corporate climate, work stressors, mental health, and *antidepressant* medications

Study 2: will look at corporate climate, work stressors, mental health, and *opioid* medications

Methods & Analysis:

Study Design:

This study will use a longitudinal design that consists of four waves of interview and self-report data using an existing data set, the Australian Workplace Barometer (AWB), a population-based longitudinal data set, matching individuals over time and linking to objective medication data (Pharmaceutical Benefits Scheme, PBS). The four waves of data from the AWB dataset include data collected in 2009-2010 (Wave 1), 2010-2011 (Wave 2), 2014-2015 (Wave 3) and 2020-2021 (Wave 4). The AWB cohort data will be linked to PBS data collected from 01/01/2009 to the latest available data. The different time points between data collection will help track the progression of effects across different time periods. The current study will commence in May 2023 and run until December 2023.

Explanation of existing data

As of the submission date, the data exists, and researchers have only had access to the AWB data. However, no detailed analysis has been conducted concerning the current research plan (including the calculation of summary statistics). Access to the AWB data has been limited, though a small portion of the data has been used to conduct exploratory analysis that showed encouraging initial results. PBS data has not been accessed at the point of registration.

Data collection procedures

For peer review only - http://bmjopen.bmj.com/site/about/guidelines.xhtml

In the first round of data collection, The Australian Workplace Barometer (AWB) used a population-based random sampling approach to conduct computer-assisted telephone interviews (CATI). Participants were recruited randomly from the Australian electronic white pages inviting household members to participate (the participant had to have had the last birthday in the household to be eligible).

For each subsequent wave of data collection, those who had consented to be re-contacted were sent a letter advising of further data collection and re-contact for a further interview. A combination of modes was used to collect subsequent waves of AWB data to increase sample representativeness and participation uptake, including telephone interviews by landline or mobile, surveys via email or SMS link, and hard copy surveys sent via post. Where participants could not partake in interviews at the first point of contact, call-backs for a more suitable time were arranged.

Telephone interviews were conducted by a team of research assistants trained to enter data immediately into the Qualtrics online survey. Participants who received an email or SMS link were provided with their participant ID (required for linkage) and entered their participant ID and self-reported data directly into the Qualtrics survey link provided. Hard copy surveys were entered manually into the Qualtrics online survey by the research team upon receipt.

Detailed information on survey items used in the study are uploaded as supplementary information (see online supplemental S1).

Payment for Participation:

In the fourth wave of data collection, participants who completed the survey automatically went into the draw to receive a gift card valued at $100. The winner of the draw was picked using a random number generator, contacted by the research team's chief investigator by phone, and announced the winner.

Inclusion criteria:

Participants are required to be a minimum of 18years of age and currently in paid employment. Sub-contractors must have worked with the same employer for a minimum of 4 months. Participants must have consented to their personal AWB information being securely sent to the Australian Institute of Health and Welfare (AIHW) to match the Pharmaceutical Benefits Scheme (PBS) dataset for research purposes.

Exclusion criteria:

Ages out of the age range, unemployed, and those who did not consent to data linkage are excluded from the dataset.

Pharmaceutical Benefits Scheme (PBS) Dataset:

National Medicare & PBS will provide data on prescription medicines, defined by the Australian Institute of Health and Welfare (AIHW) project as antidepressants to treat clinical depression and opioid medication use for this project. The Pharmaceutical Benefits Scheme (PBS) data comprises individual antidepressant and opioid medication use (summed over a year) from 2009 to 2021.

Data Linkage (AWB linkage to PBS):

PBS data was linked via AWB participants who consented to have their AWB data linked to PBS data in 2020/2021. Antidepressant medication will be matched with the AWB at the individual level, using individual names and addresses.

Data Linkage Procedure:

Step 1: A 'Request for linkage of national data: technical assessment' was completed by the second author as part of the AIHW data linkage protocol and AIHW ethics approval, which outlined the proposed research and study aims.

Step 2: AIHW was sent two files via secure email, including:

- AWB cohort personal identifiers with a data ID code
- AWB content data and with the data ID code

Step 3: AIHW linked the AWB cohort to PBS. This linkage process included the following;

- Creation of a new project-specific person number
- Loading the PBS content data and the AWB content data to the SURE project-specific person number
- All data transfers were via secure messenger service.

Important Data Linkage Notes:

- The technical assessment is a rigorous process required by AIHW to ensure that the data requested for the linkage answer the proposed research questions. The technical assessment ensures the study aims are achievable using the proposed linkage methodology and forms part of the AIHW ethics approval for the research project.
- AIHW set up a specific project ID for the linkage of the AWB and PBS data and the provision of a secure workspace, referred to as the Secure Unified Research Environment (SURE) workspace, for the analysis of linked data. All analytical software packages (SPSS, R) are loaded onto the SURE secure workspace. Deidentified AWB data set and PBS data are curated into the secure workspace.
- There are strict protocols in place during SURE access, where the original AWB dataset is held on a secure university server that restricts researcher access involved with SURE data analysis. This highly confidential workspace is constructed to hold data that blocks all access to other desktop files and software to avoid identifying deidentified participants.
- A research Assistant (the first author) will be the data custodian (not involved in the data analysis) and will be appointed during this time to ensure no local data access of identifiable/ re-identifiable data while there is access to SURE data. All data analysis takes place with the data on the secure SURE website.

Sample size

The sample included 1372 working Australians who participated in the AWB study from 2009 to 2021 and consented to the data linkage. Participants are located across all Australian states and territories; South Australia (SA), New South Wales (NSW), Western Australia (WA), Tasmania, Australian Capital Territory (ACT), and the Northern Territory (NT).

Patient and public involvement

There was no patient or public involvement in the design and development of the study.

Measured variables

All independent and dependent variables are measured at the individual level.

Demographics and workplace information:

Demographic variables in the current study include age, gender, industry, education and income. Work-related information includes work hours (i.e. shift work), length of service, working status (employed vs. unemployed), and employment status (permanent full-time, permanent part-time, casual, and fixed term contract), occupation, size of organisation, organisation business type (i.e. state government, not-for-profit) name of current organisation (optional), and lodgement of workers compensation claims.

Psychosocial Safety Climate:

The psychosocial safety climate was measured using a scale consisting of 12 items (PSC-12) [31] that assess four dimensions of PSC, including management priority, management commitment, organizational communication, and organizational participation. Items were

measured on a 5-point Likert scale, ranging from 1 (strongly disagree) to 5 (strongly agree). Scores for all 12 items are summed to provide an overall score for PSC. An example item is, “In my workplace, senior management acts quickly to correct problems/issues that affect employees’ psychological health”.

Psychosocial Demands:

1. Work pressure

Work pressure was measured using the 5-item psychological job demands scale from the Job Content Questionnaire (JCQ 2.0) [32], an example item “My job requires working very fast”. Items were measured on a 4-point Likert scale, ranging from 1 (strongly disagree) to 4 (strongly agree). The five items were summed to give a total score for the work pressure.

2. Workplace bullying:

Bullying was assessed using an amended version of the QPSNordic Bullying Questionnaire [33]. Participants were provided with a definition of workplace bullying and then answered questions about their exposure to bullying in the workplace.

Definition provided to participants; “Bullying is a problem at some workplaces and for some workers. To label something, as bullying, the offensive behaviour has to occur repeatedly over a period of time, and the person confronted has to experience difficulties defending him or herself. The behaviour is not bullying if two parties of approximate equal ‘strength’ are in conflict or the incident is an isolated event” [34].

Participants were then asked, “Have you been subjected to bullying at the workplace during the last six months?” Those who replied with ‘yes’, were then asked by whom (supervisor/co-worker) and the length and frequency of exposure to workplace bullying.

3. Workplace harassment

Seven items from Richman et al.’s (1996) scale [35] were used to measure workplace harassment. Items were measured on a 5-point Likert scale, ranging from 1 (very rarely/never) to 5 (very/often always) and summed together to represent a total for workplace harassment. An example item is “I have been sworn at and/or yelled at”.

Psychosocial demands (work pressure, workplace bullying & workplace harassment) are independent variables., all measured longitudinally.

Psychosocial Resources

1. Job control

Three subscales were used from the JCQ 2.0 [32] to measure job control, including the 5-item skill discretion subscale ("My job requires that I learn new things"), the 3-item decision authority subscale ("In my job I have very little freedom to decide how I do my work") and the 3-item macro-decision latitude subscale ("In my organization, I have significant influence over decisions made by my work team and department"). All 12 items were measured on a 4-point Likert scale, ranging from 1 (strongly disagree) to 4 (strongly agree). Items were summed to provide a total for job control.

2. Social Support

To measure social support, six items from the JCQ 2.0 [32] was used, three items for supervisor social support ("My supervisor/manager is helpful in getting the job done") and three items for co-worker support ("People I work with are friendly"). Items were measured on a 4-point Likert scale, ranging from 1 (strongly disagree) to 4 (strongly agree) and summed for both supervisor and co-worker to provide a total score for each social support scale.

Job control (skill discretion, decision authority & macro-decision latitude) and social support (supervisor and co-worker) are independent variables, measured longitudinally.

Distress Symptoms

1. Psychological distress

The Kessler 10 scale [36] was used to measure psychological distress. A 5-point Likert scale measured responses, ranging from 1 (none of the time) to 5 (all of the time). Scores on the ten items were totalled to give an overall score on psychological distress. An example item is "[In the past four weeks], about how often did you feel tired out for no good reason?".

2. Emotional exhaustion

Five items from the Maslach Burnout Inventory [37] were used to measure emotional exhaustion on a 7-point Likert scale, ranging from 1 (never) to 7 (every day). An example item is, "I feel burned out from my work."

Distress symptoms and/or mental health problems are dependent variables measured longitudinally (four-time points) using psychological distress and emotional exhaustion.

Work Engagement

Work engagement was measured using three items from the Utrecht Work Engagement Scale – Shortened Version (UWES-9) [38], covering vigour, dedication and absorption. Items were measured on a 7-point Likert scale, ranging from 1 (never) to 7 (every day) and summed together to provide an overall score for work engagement. An example item is "At work I feel bursting with energy".

Engagement will be used as a dependent variable, measured longitudinally.

Stigma/Discrimination

Stigma/discrimination was measured using one item from the Kessler 6 (K6) mental health symptom screening questionnaire ("In the last 12 months, do you think you have had emotional or mental health problems?"). If participants answered 'yes', a further seven questions were asked about their experiences within the workplace of discrimination ("Have any of your supervisors and or managers treated you unfairly or discriminated against you because of the mental health problems?"), avoidance ("Have any of your supervisors and or managers avoided you because of the emotional or mental health problems?") and positive treatment ("Because of your mental health or emotional problems have you been treated more positively by your employer?") [39].

Stigma/discrimination was only measured in wave 4 (2020/2021).

Mental Health Problems

1. Depression

All nine items from the Patient Health Questionnaire (PHQ-9) [40] were used to measure depressive symptoms in the present study, with a time reference modified for the last four weeks, an example item "[During the last month, how often were you bothered by] feeling down, depressed, or hopeless?". Items were measured on a 4-point Likert scale, from 1 (not all) to 4 (nearly every day). Scores were totalled to give an overall score on depression.

2. Suicidal ideation

One item from the Patient Health Questionnaire (PHQ-9) [40] was used to measure suicidal ideation ("[During the last month, how often were you bothered by] thoughts that you would be better off dead or thoughts of hurting yourself in some way?").

Depression and suicidal ideation are dependent variables, measured longitudinally (four-time points).

Prescribed Medication Usage

1. Self-report prescribed medication usage data

Participants were asked the question, "Have you taken any prescription medications for your mental health in the last 12 months?". Those who answered 'yes' were then asked to indicate from a list of prescribed medications (sleeping tablets/capsules, tablets/capsules for anxiety or nerves, tranquillizers, antidepressants, mood stabilizers, and other medications for your mental health) which medications they had used in the last 12 months, and also in the last four weeks. Antidepressant medication is a dependent variable measured only in wave 4 (2020/2021).

2. Registered PBS Medication Data

PBS variables include PBS item, drug type, benefit amount, the patient contribution amount, under co-payment prescription type, prescribed speciality, prescriber type, date of prescribing, number of scripts dispensed, quantity supplied, date of supply, and patient category.

Supplemental Material:

S1. Australian Workplace Barometer (AWB) Survey for Wave 4 (2020/2021)

Data Analysis Plan:

The proposed theoretical models will be analysed using data linked from the AWB and PBS, mainly using ANOVA, structural equation modelling (SEM), hierarchical linear modelling (HLM), where applicable, and logistic regression.

ANOVA

By using the depression score over time, we will categorise the sample into four categories, i.e., depressed, recovering, worsening, and healthy to capture the changes in their mental health condition over ten years. We will conduct ANOVA to identify the differences in their reported corporate climate and working conditions.

Structural Equation Modeling (SEM) will be used with time-lagged effects to estimate the direction and strength of the hypothesised relationships between work factors, stress reactions, and medication use. We will evaluate model fit using indices like chi-squared tests, RMSEA, CFI, and TLI. With time-lagged SEM, we will be able to depict how variables and their associated relationships with other variables change over time (e.g., how a change in PSC would influence the change in distress reactions in the future). To better handle the varied time intervals in the AWB data, we will use continuous time SEM (CTSEM). CTSEM, with a Bayesian approach, allows individual-varying specifications for time intervals and other parameters in the analysis, addressing potential issues due to the unequal intervals between measurements and providing inferences on continuous processes over time. We will also estimate within-person and between-person effects simultaneously by modelling both observed covariates and unobserved heterogeneity to gain a more nuanced understanding of the tranquilising effects of medication on work stress and the influence of PSC in the process pathway.

Hierarchical Linear Modeling (HLM) will be used to account for hierarchical data structures, such as individuals nested within organisations (if the data allows) or multiple occasions within individuals. Using HLM, we will investigate how work factors and stress reactions vary at both the individual and organisation levels, and their impact on medication use.

Logistic regression will be conducted for any potential binary classification problems, such as the use of medication predicting whether workers will use medication (yes/no) based on the levels of work stress or poor organisational climate they are exposed. Odds ratios will be calculated to estimate the risk level of exposure to poor work conditions and low levels of PSC.

Cost estimation

The cost associated with mental health issues and medication use attributable to work-related factors will be calculated by the total cost of medication usage and medical compensation claims through PBS data and the attributable fractions (AFs) of poor work condition, PSC, and work stress. AFs can be estimated, for example, by using an empirical established threshold, the PSC benchmark [41] to identify the proportion of exposed and unexposed workers to poor PSC and work condition.

Exploratory Analysis Plan

We will investigate the role of stigma/discrimination and PSC for workers experiencing work stress/distress symptoms and their association with anti-depressant and opioid medication use to determine whether the corporate climate tranquillizes the effects of work stress and anti-depressant and opioid use and whether stigma moderates these effects. We will explore mental health vulnerability as a predictor by assessing a range of indicative states suggested by mental health self-reports. Instead of controlling for this as prior research [42], we will investigate its trajectory, subsequent stressor exposure (e.g., bullying), and future mental health status. This has implications for how mental health is managed in the workplace. We will also investigate the validity of self-report medication use data against PBS data (registered data).

Ethics & Dissemination:
The study has been reviewed and approved by the Human Research Ethics Committee of the University of South Australia (Ethics Protocol: 203003). Further, approval from the Australian Institute of Health and Welfare Ethics Committee was also granted for the linkage of AWB data and PBS data (EthOS Application EO2022/1/1190).

Results of the study will be disseminated through worldwide keynotes, key international settings such as the Joint Congress of ICOH-WOPS, high-impact peer-reviewed journals, industry conference presentations, and various media outlets (such as LinkedIn, Twitter, radio, community forums) to reach managers, workers and industry partners. Further, UniSA requires publications from public projects to be held in an institutional repository which fulfils the Australian Research Council's Open Access Policy. Therefore, we will also lodge relevant outputs with the Analysis and Policy Observatory collection for greater accessibility.

Author Contributions:

Cherie Crispin : Ms Crispin was involved in collection of longitudinal data since 2020. She will assist with preparation and drafting of study papers, ethics applications, development of study protocol, development of model design and theoretical framework for Study Two (corporate climate, work stressors, mental health and *opioid* medications), provision of AWB data sets for data linkage agencies, liaison with data linkage agencies.

Dr Ali Afsharian : Dr Afsharian was involved in collection of longitudinal data since 2020. He will assist with ethics applications, progress reports, drafting papers and study protocol, development of methodology, model design and theoretical framework for Study One (corporate climate, work stressors, mental health, and *antidepressant* medications), liaise with data linkage agencies and will be one of three researcher team members to perform computations and statistical analysis.

Dr May Young Loh : Dr Loh will assist with theory development, methodology and model design for Studies One and Two and will be one of three research team members to perform computations and complex statistical analysis.

Professor Maureen Dollard : Prof Dollard conceived of the idea, developed and contributed to the original design and implementation of the research. Prof Dollard will lead, supervise and coordinate the research team and take overall responsibility for the strategic intellectual direction of the project and communication of results. Further, she will assist with theory development, methodology and model design for Studies One and Two and will be one of three research team members to perform computations and statistical analysis.

For peer review only

Professor Christian Dormann : Prof Dormann will assist the team in integrating and testing new societal cost elements of Psychosocial Safety Climate (PSC) theory using R, structural equational modelling, and continuous time modelling, and contribute to publication of results.

Dr Nick Glozier : Dr Glozier will be responsible for aspects of data linkage, mental health and health service evaluation, and PBS medication interpretation and will contribute to the costing and epidemiological aspects of the study design.

Dr Tiffany Gill : Dr Gill is an epidemiologist who was involved in the original development and implementation of the AWB surveys in 2009. She will provide advice on survey, ethical and other project related issues as required.

Professor Anne Taylor : Prof Taylor will assist with expert advice on interpretation of data analysis and data-linkage. Prof Taylor was originally involved with sampling, the set-up of Computer Assisted Telephone Interviewing (CATI) and data collection. She will assist with the review of papers where required.

All authors have reviewed and approved the final version of the study protocol.

Funding Statement:

This work was supported by five grants: (1) Australian Research Council (ARC) DP200102752 (103319) Dollard M.F. (2) DP087900 Australian Research Council Discovery Grant. Dollard MF, Winefield AH, LaMontagne AD, Taylor AW, Bakker AB, Mustard C. Working wounded or engaged? Australian work conditions and consequences through the lens of the job demands resources model. (3) LP100100449 Australian Research Council Linkage Grant. Dollard MF, Winefield AH, Taylor AW, Smith PM, Nafalski A, Bakker A, Dormann C. State, organisational, and team interventions to build a psychosocial safety climate using the Australian Workplace Barometer and the StressCafé. (4) FL200100025 Australian Research Council Laureate Fellowship. (5) Safework NSW's Centre for WHS.

Competing interests' statement.

None declared

References:

1. World Health Organisation (WHO). Closing the gap in a generation: Health equity through action on the social determinants of health [online]. 2008. https://www.who.int/publications/i/item/WHO-IER-CSDH-08.1 (accessed on 14 March 2023).
2. International Labour Organisation (ILO). World Employment and Social Outlook: Trends 2016 [online]. 2016. https://www.ilo.org/wcmsp5/groups/public/@dgreports/@dcomm/@publ/documents/publication/wcms_443480.pdf (accessed on 14 March 2023).
3. World Health Organisation (WHO). Mental health at work [online]. 2022. https://www.who.int/news-room/fact-sheets/detail/mental-health-at-work
4. Australian Bureau of Statistics. National study of mental health and wellbeing [online] 2022. https://www.abs.gov.au/statistics/health/mental-health/national-study-mental-health-and-wellbeing/latest-release (accessed 28 February 2023).
5. Australian Bureau of Statistics. Causes of death, Australia [online]. 2022. https://www.abs.gov.au/statistics/health/causes-death/causes-death-australia/latest-release (accessed 28 February 2023).
6. Lunghi, C., Dugas, M., Leclerc, J., et al. (2022). Global prevalence of antidepressant drug utilization in the community: protocol for a systematic review. BMJ Open. http://doi.org/10.1136/bmjopen-2022-062197

For peer review only

7. Whiteford, H.A., Degenhardt, L., Rehm, J., et al. (2013). Global burden of disease attributable to mental and substance use disorders: findings from the Global Burden of Disease Study 2010. Lancet 382, 1575–1586. https://doi.org/10.1016/S0140-6736(13)61611-6
8. Australian Institute of Health and Welfare. Mental Health Prescriptions [online]. 2022. https://www.aihw.gov.au/mental-health/topic-areas/mental-health-prescriptions (accessed 28 February 2023).
9. Lalic, S., Ilomaki, J., Bell, J. S., et al. (2019). Prevalence and incidence of prescription opioid analgesic use in Australia. British Journal of Clinical Pharmacology, 85, 202-215. https://doi.org/10.1111/bcp.13792
10. Wilson, J., Lam, T., Scott., D., et al. (2020). 'Extreme personal stress' and 'a lot of pain': Exploring the physical, mental and social contexts of extramedical pharmaceutical opioid use in Australia paramedic case descriptions. Drug and Alcohol Review, *39,* 870-878. https://doi.org/10.1111/dar.13118
11. Australian Institute of Health and Welfare. Opioid harm in Australia; and comparisons between Australia and Canada [online]. 2018. https://www.aihw.gov.au/getmedia/605a6cf8-6e53-488e-ac6e-925e9086df33/aihw-hse-210.pdf.aspx?inline=true (accessed on 28 February 2023).
12. beyondblue. The State of Workplace Mental Health in Australia [online]. 2015. https://www.headsup.org.au/docs (accessed on 28 February 2023).
13. Safe Work NSW. Mentally healthy workplaces in NSW [online]. 2017. https://www.safework.nsw.gov.au/__data/assets/pdf_file/0018/320139/Mentally-healthy-workplaces-in-NSW-benchmarking-tool-October-2017-SW08734.pdf (accessed on 14 March 2023).
14. Safe Work Australia. Compendium of Workers' Compensation Statistics Australia 2015-16 [online]. 2017b. https://www.safeworkaustralia.gov.au (accessed on 14 March 2023).
15. Lahti, J., Lallukka, T., Harkko, J., et al. (2021). Working conditions and antidepressant medication use: A prospective study among 18 to 39-year-old municipal employees. Psychiatry Research, 305, 114213, https://doi.org/10.1016/j.psychres.2021.114213
16. Theorell, T., Hammarstrom, A., Aronsson, G., et al. (2015). A systematic review/ meta-analysis of work environment and depressive symptoms. BMC Public Health, 15, 738. https://doi.org/10.1186/s12889-015-1954-4
17. Woo, J.M., Postolache, T.T., 2008. The impact of work environment on mood disorders and suicide: Evidence and implications. International Journal of Disability. Human Development. 7, 185–200. https://doi.org/10.1515/ijdhd.2008.7.2.185
18. Karasek, R. & Theorell, T. (1990). Healthy Work. New York: Basic Books
19. Siegrist, J (1996). Adverse health effects of high-efforts/low-reward conditions. Journal of Occupational Health Psychology,1, 27–41. https://doi.org/10.1037//1076-8998.1.1.27
20. Leach, LS., Poyser, C. & Butterworth. (2016). Workplace bullying and the association with suicidal ideation/thoughts and behaviour: a systematic review. Occupational and Environmental Medicine, 74(1). https://doi.org/10.1136/oemed-2016-103726
21. Loerbroks, A., Cho, S., Dollard, M.F., et al. (2016). Associations between work stress and suicidal ideation. Journal of Psychosomatic Research, 90, 62–69. https://doi.org/10.1016/j.jpsychores.2016.09.008
22. Parker, SK, Morgeson, FP, & Johns, G. (2017). One hundred years of work design research. Journal of Applied Psychology, 102(3), 403. https://doi.org/10.1037/apl0000106
23. Dollard, MF & Bakker, AB. (2010). Psychosocial safety climate as a precursor to conducive work environments, psychological health problems, and employee engagement. Journal of Occupational and Organizational Psychology, 83, 579–599. https://doi.org/10.1348/096317909X470690
24. Dollard, MF & Karasek, R. (2010). Building psychosocial safety climate: Evaluation of a socially coordinated PAR risk management stress prevention. In J Houdmont & S Leka

For peer review only

(Eds), Contemporary occupational health psychology (p. 208–234). Chichester: Wiley Blackwell. https://doi.org/10.1002/9780470661550.ch11

25. Law, R, Dollard, M.F., Tuckey, M.R., et al. (2011). Psychosocial safety climate as a lead indicator of workplace bullying and harassment, job resources, psychological health and employee engagement. Accident Analysis and Prevention, 43(5), 1782–1793. https://doi.org/10.1016/j.aap.2011.04.010
26. Idris, MA, Dollard, MF, Coward, J., et al. (2012). Psychosocial safety climate: conceptual distinctiveness and effect on job demands and worker psychological health. Safety Science, 50, 19–28. https://doi.org/10.1016/j.ssci.2011.06.005
27. Griffin, M, & Curcuruto, M. (2016). Safety Climate in Organizations. Annual Review of Organizational Psychology and Organizational Behavior, 3(1). https://doi.org/10.1146/annurev-orgpsych-041015-062414
28. McLinton, S. S., Afsharian, A., Dollard, M. F., et al. (2019). The dynamic interplay of physical and psychosocial safety climates in frontline healthcare. *Stress & Health, 35*(5), 650-664. https://doi.org/10.1002/smi.2898
29. Demerouti, E., Bakker, A., B., Nachreiner, F., et al. (2001). The job demands-resources model of burnout. Journal of Applied Psychology, 86, 499–512. PMID: 11419809
30. Dollard, M. F. & Bailey, T. (2014). The Australian workplace barometer: Psychosocial safety climate and working conditions in Australia. Samford Valley, QLD: Australian Academic Press Group Pty Ltd.
31. Hall, G. B., Dollard, M. F., Coward, J. (2010). Psychosocial Safety Climate: Development of the PSC-12. International Journal of Stress Management, 4, 353–383. https://doi.org/10.1037/a0021320
32. Karasek, R., Brisson, C., Kawakami, N., et al. (1998). The Job Content Questionnaire (JCQ): an instrument for internationally comparative assessments of psychosocial job characteristics. Journal of Occupational Health Psychology, 3(4), 322–355. https://doi.org/10.1037//1076-8998.3.4.322
33. Elo A.-L., Dallner M., Gamberale F., et al. (2000). Validation of the Nordic Questionnaire for Psychological and Social Factors at Work—QPSNordic. In M. Vartiainen, F. Avallone, & N. Anderson (Eds.), *Innovative theories, tools, and practices in work and organizational psychology* (pp. 47–57). Hogrefe & Huber Publishers.
34. Ørhede, E., Hottinen, V., Skogstad, A., et al. (2000). User's Guide for QPSNordic. General Nordic questionnaire for psychological and social factors at work. Copenhagen: Nordic Council of Minister.
35. Richman, J. A., Flaherty, J. A., & Rospenda, K. M. (1996). Perceived workplace harassment experiences and problem drinking among physicians: Broadening the stress/alienation paradigm. Addiction, 91(3), 391–403. https://doi.org/10.1046/J.1360-0443.1996.9133918.X
36. Kessler, R. & Mroczek, D. (1994). Final Version of our Non-specific Psychological Distress Scale. Ann Arbort (MI): Survey Research Center of the Institute for Social Research, University of Michigan.
37. Schaufeli, W., Leiter, M., Maslach, C. et al. (1996) Maslach Burnout Inventory-General Survey. In: Maslach, C., Jackson, S.E. and Leiter, M.P., Eds., The Maslach Burnout Inventory: Test Manual, Consulting Psychologists Press, Palo Alto, CA.
38. Schaufeli, W. B., Bakker, A. B., & Salanova, M. (2006). The Measurement of Work Engagement With a Short Questionnaire: A Cross-National Study. *Educational and Psychological Measurement, 66(*4), 701–716. https://doi.org/10.1177/0013164405282471
39. Reavley, N. J., Morgan, A. J. & Jorm, A. F. (2016). Disclosure of mental health problems: findings from an Australian national survey. Epidemiology and Psychiatric Sciences, 1-11. https://doi.org/10.1017/S204579601600113X
40. Spitzer, R L., Kroenke, K. & Williams, J. B. (1999). Validation and utility of a self-report version of PRIME-MD: The PHQ primary care study. JAMA. https://doi.org.10.1001/jamma.282.18.1737

41. Bailey, T. S., Dollard, M. F., McLinton, S. S., et al. (2015): Psychosocial safety climate, psychosocial and physical factors in the aetiology of musculoskeletal disorder symptoms and workplace injury compensation claims, Work & Stress: An International Journal of Work, Health & Organisations 29(2), 190-211. https://doi.org/10.1080/02678373.2015.1031855
42. Virtanen, M., Honkonen, T., Kivimaki, M. et al. (2007). Work stress, mental health and antidepressant medication findings from the Health 2000 Study. Journal of Affective Disorders, 98, 189–97. https://doi.org/10.1016/j.jad.2006.05.034

For peer review only

UniSA | Justice & Society

University of South Australia
Australia's University of Enterprise

Australian Workplace Barometer Survey (2021)

INFORMATION SHEET

Welcome to the Australian Workplace Barometer (AWB) survey.

Professor Maureen Dollard, Director of the PSC Observatory, for the University of South Australia is conducting the study with funding from the Australian Research Council.

This Australian Research Council national study aims to improve the health of Australian workers. Questions will be asked about working conditions, job security, psychological health, physical health, heart health, work engagement, satisfaction, fatigue, and workers' compensation.

We previously contacted you in 2014 about the Australian Workplace Barometer survey and you kindly gave us permission to contact you again regarding future surveys. Please complete the double-sided survey attached and return in the enclosed reply-paid envelope. Alternatively, if you wish to complete the survey online, please type the following link into your web browser http://bit.ly/AWB-Survey, ensuring to insert your Participant ID below into the survey link and complete online. Once you have completed the survey you will go into the draw to win $100.

For additional information about the Australian Workplace Barometer, please visit website https://bit.ly/AWB-InfoSheet

We greatly appreciate your support and contribution to the AWB project. If you experience any issues, please contact the AWB Team on 0422 643 737.

Kind regards

Dr Ali Afsharian
Research Associate
PSC Observatory / Centre for Workplace Excellence (CWeX)
University of South Australia
Email: ali.afsharian@unisa.edu.au

Participant's ID:

For peer review only - http://bmjopen.bmj.com/site/about/guidelines.xhtml

Australian Workplace Barometer (AWB) Survey

1. Are you still in paid employment or self-employed or a sub-contractor?
- ○ Employed (skip to Q.3)
- ○ Sub-contractor (skip to Q.5)
- ○ Self-employed
- ○ No longer in paid employment or self-employed or a subcontractor (go to Q.2)

If answered 'Self-employed', you will not be able to complete the remainder of the survey as the survey is for current employed workers and their workplace conditions. Thank you for your time.

2. What is the reason that you are no longer in paid employment or self-employed or a sub-contractor?
- ○ Retired
- ○ Made redundant
- ○ Maternity leave
- ○ Student
- ○ Other (specify): ___________________

You will not be able to complete the remainder of the survey as the survey is for current workers and their workplace conditions. Thank you for your time.

3. Is your employer currently receiving Job Keeper benefits for you?
- ○ Yes (go to Q.4)
- ○ No (skip to Q.6)
- ○ Not sure

4. Are you currently participating in work-related tasks or duties?
- ○ Yes (skip to Q.6)
- ○ No

If 'No', you will not be able to complete the remainder of the survey as the survey is for current workers and their workplace conditions. Thank you for your time.

5. Has your current contract involved working for the same employer for the past 4 months or longer?
- ○ Yes
- ○ No

If 'Yes', would you prefer an alternative employment status?
- ○ Permanent full-time
- ○ Permanent part-time
- ○ Casual/temporary (no annual/sick leave)
- ○ Fixed-term contract
- ○ Other (specify): ____________________

(skip to Q.7)

If 'No', you will not be able to complete the remainder of the survey as the survey is for current employed workers and their workplace conditions. Thank you for your time.

Employment

The following questions relate to your main job, that is the one in which you work most hours.

6. What is your employment status?
- ○ Permanent full-time
- ○ Permanent part-time
- ○ Casual/temporary (no annual/sick leave)
- ○ Fixed-term contract
- ○ Other (specify): ______________________

7. Has your employment status changed due to the Covid-19 pandemic?
- ○ No
- ○ Yes, from full-time to part time
- ○ Yes, from full-time to casual
- ○ Other (specify): __________________

8. At this moment, which do you best identify with?
- ○ The majority of my work is done remotely
- ○ I normally split my time between home and or on-site, but now I'm only working remotely
- ○ I normally work on-site but now I'm working remotely
- ○ I am still working on-site

For peer review only - http://bmjopen.bmj.com/site/about/guidelines.xhtml

9. About how long have you been working for your current employer?
Enter years: ________________
Enter months: ________________

10. What is the main business, industry or service of your employer?
- Accommodation, cafes and restaurants
- Agriculture, forestry and fishing
- Communications services
- Construction
- Cultural and recreational services
- Education
- Electricity, gas and water supply
- Finance and insurance
- Government administration and defence
- Health and community services
- Manufacturing
- Mining
- Personal and other services
- Property and business services
- Retail trade
- Transport and storage
- Wholesale trade
- Other (specify): _____________

11. Is your employers business a…..?
- A commonwealth or federal government department or agency
- A state or local government agency
- A not-for-profit, religious, or community organisation
- A private sector business
- Something else (specify): _____________

12. With regard to number of employees – what size organisation do you work in?
- MICRO (less than 2)
- SMALL (2-19)
- MEDIUM (20-200)
- LARGE (200+)
- Don't know

13. Are you a member of a union or employee organisation that looks after employees' interests (e.g., Nursing Federation, Police Association)?
- Yes
- No
- Don't know

14. Are you paid for any overtime you do?
- Always
- Sometimes
- Never
- Don't do any overtime

15. Which time period do you usually work?
- Day
- Evening
- Night
- Rotating shifts
- Other (specify): ________________

16. Have you ever put in a worker's compensation claim?
- Yes (go to Q.17)
- No (skip to Q.21)

17. Did you lodge this claim in the last 12 months?
- Yes (go to Q.18)
- No (skip to Q.21)

18. Was the most recent claim for a stress or psychological injury?
- Yes (go to Q.19)
- No (skip to Q.20)

19. Reason for the most recent claim stress or psychological injury claim?
- Exposure to a traumatic event
- Exposure to workplace or occupational violence
- Work related harassment and/or workplace bullying
- Work Pressure
- Other mental stress factors
- Other harassment
- Other (specify): ______________

(skip to Q.21)

20. Was the most recent claim for neck, back, or joint pain or muscle injury?
- Yes
- No

For peer review only - http://bmjopen.bmj.com/site/about/guidelines.xhtml

Job Demands - Psychological Demands
Below are some statements about your work. Could you please tick below if you disagree or agree and to what level ...?

	Strongly Disagree	Disagree	Agree	Strongly Agree
**21.** My job requires working very fast.	○	○	○	○
**22.** My job requires working very hard.	○	○	○	○
**23.** I am not asked to do an excessive amount of work.	○	○	○	○
**24.** I have enough time to get the job done.	○	○	○	○
**25.** Some demands I face at work are in conflict with other demands at work.	○	○	○	○

Job Demands - Physical Demands
Below are some statements about your work. Could you please tick below if you disagree or agree and to what level ...?

	Strongly Disagree	Disagree	Agree	Strongly Agree
**26.** My job requires lots of physical effort.	○	○	○	○
**27.** I am often required to move or lift very heavy loads on my job.	○	○	○	○
**28.** I am often required to work for long periods with my body in physically awkward positions.	○	○	○	○

Workplace Physical Risk Factors
I am required to:

	Almost never	Sometimes	Often	Almost always
**29.** Keep repeating the same movements, every minute or so.	○	○	○	○
**30.** Lift or carry things that are moderately (or very) heavy.	○	○	○	○
**31.** Push or pull things using some force.	○	○	○	○
**32.** Work in twisted or awkward postures.	○	○	○	○
**33.** Squat or kneel while you work.	○	○	○	○

Emotional Demands

	Strongly Disagree	Disagree	Agree	Strongly Agree
**34.** My work is emotionally demanding.	○	○	○	○
**35.** My work places me in emotionally challenging situations.	○	○	○	○
**36.** My work requires suppressing my genuine emotion.	○	○	○	○
**37.** I have to act the way people think a person in my position should act.	○	○	○	○

For peer review only - http://bmjopen.bmj.com/site/about/guidelines.xhtml

**Organisational Characteristics - Demands Organisational Change**<br>The next set of questions concerns things that you may or may not experience in your work.				
	**Strongly Disagree**	**Disagree**	**Agree**	**Strongly Agree**
**38.** In your company/organisation, there have been changes such as restructuring, downsizing, and layoffs that have significantly affected your job.	○	○	○	○
**39.** In your company/organisation, new policies and procedures designed to cut costs are constantly being introduced where you work.	○	○	○	○
**40.** In your company/organisation, there is frequent management turnover.	○	○	○	○

**Organisational Harassment**<br>Sometimes in your workplace you may experience or witness harassment from your manager/supervisor or colleagues. Can you please answer with the best response to these statements?					
	**Very rarely/never**	**Rarely**	**Sometimes**	**Often**	**Very often/ always**
**41.** I have experienced unwanted sexual advances.	○	○	○	○	○
**42.** I have experienced discomfort listening to sexual humour.	○	○	○	○	○
**43.** I have experienced unfair treatment because of my gender.	○	○	○	○	○
**44.** Negative comments have been made regarding my ethnic or racial background.	○	○	○	○	○
**45.** I have been sworn at and or yelled at.	○	○	○	○	○
**46.** I have been humiliated in front of others.	○	○	○	○	○
**47.** I have experienced being physically assaulted and or threatened by members of the organisation.	○	○	○	○	○
**48.** I have experienced being physically assaulted and or threatened, by a client or patient.	○	○	○	○	○

For peer review only

For peer review only - http://bmjopen.bmj.com/site/about/guidelines.xhtml

Organisational Bullying
Bullying is a problem at some workplaces and for some workers. To label something as bullying, the offensive behaviour has to occur repeatedly over a period of time, and the person confronted has to experience difficulties defending him or herself. The behaviour is not bullying if two parties of approximate equal "strength" are in conflict or the incident is an isolated event.

49. Have you been subjected to bullying at the workplace during the last six months?
- Yes (go to Q.50)
- No (skip to Q.53)

50. how often were you exposed to these bullying behaviours overall?
- Daily
- At least once per week
- At least once per month
- Rarely
- Never

51. How long were you exposed to these bullying behaviours overall?
- Less than one month
- 1-6 months
- 7-12 months
- 1-2 years
- More than 2 years

52. Was the bully a …?
- A supervisor
- A co-worker
- Neither
- Both

Work-Family Conflict
Below are some statements about how work can affect your home and personal life.
Could you please tick below if you disagree or agree and to what level ...?

	Strongly Disagree	Disagree	Slightly Disagree	Neither Agree nor Disagree	Slightly Agree	Agree	Strongly Agree
**53.** The demands of my work interfere with my home and family life.	○	○	○	○	○	○	○
**54.** My job produces strain that makes it difficult to fulfil family duties.	○	○	○	○	○	○	○
**55.** Due to work-related duties, I have to make changes to my plans for family activities.	○	○	○	○	○	○	○

For peer review only - http://bmjopen.bmj.com/site/about/guidelines.xhtml

Psychosocial Safety Climate
The questions and statements in this section are about the psychological health and safety at your workplace. Could you please tick below if you disagree or agree and to what level ...?

	Strongly Disagree	Disagree	Neither Disagree or Agree	Agree	Strongly Agree
**56.** In my workplace senior management acts quickly to correct problems/issues that affect employees' psychological health.	○	○	○	○	○
**57.** Senior management acts decisively when a concern of an employees' psychological status is raised.	○	○	○	○	○
**58.** Senior management show support for stress prevention through involvement and commitment.	○	○	○	○	○
**59.** Psychological well-being of staff is a priority for this organisation.	○	○	○	○	○
**60.** Senior management clearly considers the psychological health of employees to be of great importance.	○	○	○	○	○
**61.** Senior management considers psychological health to be as important as productivity.	○	○	○	○	○
**62.** There is good communication here about psychological safety issues which affect me.	○	○	○	○	○
**63.** Information about workplace psychological well-being is always brought to my attention by my manager/supervisor.	○	○	○	○	○
**64.** My contributions to resolving occupational health and safety concerns in the organisation are listened to.	○	○	○	○	○
**65.** Participation and consultation in psychological health and safety occurs with employees', unions and health and safety representatives in my workplace.	○	○	○	○	○
**66.** Employees are encouraged to become involved in psychological safety and health matters.	○	○	○	○	○
**67.** In my organisation, the prevention of stress involves all levels of the organisation.	○	○	○	○	○

For peer review only

Resources - Skill Discretion
The next set of questions concerns some characteristics of your work. Please answer with the best response that suits your workplace.

	Strongly Disagree	Disagree	Agree	Strongly Agree
**68.** My job requires that I learn new things.	○	○	○	○
**69.** My job involves a lot of repetitive work.	○	○	○	○
**70.** My job requires me to be creative.	○	○	○	○
**71.** My job requires a high level of skill.	○	○	○	○
**72.** I get to do a variety of different things in my job.	○	○	○	○
**73.** I have an opportunity to develop my own special abilities.	○	○	○	○

Decision Authority

	Strongly Disagree	Disagree	Agree	Strongly Agree
**74.** My job allows me to make a lot of decisions on my own.	○	○	○	○
**75.** In my job, I have very little freedom to decide how I do my work.	○	○	○	○
**76.** I have a lot to say about what happens in my job.	○	○	○	○

Macro-Decision Latitude

	Strongly Disagree	Disagree	Agree	Strongly Agree
**77.** In my company/organisation, I have significant influence over decisions made by my work team or department.	○	○	○	○
**78.** In my company/organisation, all parties are represented for major decisions.	○	○	○	○
**79.** In my company/organisation, we are always informed well in advance about major decisions.	○	○	○	○

Supervisor Social Support

	Strongly Disagree	Disagree	Agree	Strongly Agree
**80.** My supervisor/manager is concerned about the welfare of those under him/her.<br>I have no supervisor/manager (skip to Q.83)	○	○	○	○
**81.** My supervisor/manager is helpful in getting the job done.	○	○	○	○
**82.** I am treated with respect by my supervisor/manager.	○	○	○	○

For peer review only - http://bmjopen.bmj.com/site/about/guidelines.xhtml

Co-worker Social Support

	Strongly Disagree	Disagree	Agree	Strongly Agree
**83.** People I work with are friendly.<br>I have no coworkers (skip to Q.86)	○	○	○	○
**84.** People I work with are helpful in getting the job done.	○	○	○	○
**85.** I am treated with respect by my co-workers.	○	○	○	○

Recovery

	Strongly Disagree	Disagree	Slightly Disagree	Neither Agree nor Disagree	Slightly Agree	Agree	Strongly Agree
**86.** I rarely recover my strength fully between work periods.	○	○	○	○	○	○	○
**87.** I'm often still feeling fatigued from one work period by the time I start the next one.	○	○	○	○	○	○	○

Organisational Characteristics - Resources Organisational Rewards

	Strongly Disagree	Disagree	Agree	Strongly Agree
**88.** Considering all my efforts and achievements, I receive the respect and prestige I deserve at work.	○	○	○	○
**89.** Considering all my efforts and achievements, my job prospects are adequate.	○	○	○	○
**90.** Considering all my efforts and achievements, my salary/income is adequate.	○	○	○	○
**91.** My job security is poor.	○	○	○	○

For peer review only - http://bmjopen.bmj.com/site/about/guidelines.xhtml

Psychosocial Safety Climate Policy

92. Does your establishment have a procedure to deal with work-related stress?
- Yes
- No
- Work-related stress is not an issue in our establishment
- Don't know

93. Is there a procedure in place to deal with bullying or harassment?
- Yes
- No
- These problems are not an issue in our establishment
- Don't know

94. And do you have a procedure to deal with work-related violence?
- Yes
- No
- Work-related violence is not an issue in our establishment
- Don't know

95. What about the role of employees: Have they been consulted regarding measures to deal with psychosocial risks?
- Yes
- No
- Don't know

96. Are employees encouraged to participate actively in the implementation and evaluation of the measures?
- Yes
- No
- Don't know

Job Redesign

In the last 3 years, has your establishment used any of the following measures to deal with psychosocial risks?

97. Changes to the way work is organised?
- Yes
- No
- Don't know

98. A redesign of the work area?
- Yes
- No
- Don't know

99. Set-up of a conflict resolution procedure?
- Yes
- No
- Don't know

Engagement & Happiness

We also need to know about your levels of engagement in your work. Please answer with the best response.

Vigour

100. At my work, I feel bursting with energy.
- Never
- 1-3 times per year or less
- Once a month
- A few times a month
- Once a week
- A few times a week
- Every day

Dedication

101. I am enthusiastic about my job.
- Never
- 1-3 times per year or less
- Once a month
- A few times a month
- Once a week
- A few times a week
- Every day

Absorption

102. I am immersed in my work.
- Never
- 1-3 times per year or less
- Once a month
- A few times a month
- Once a week
- A few times a week
- Every day

Happiness

The next question is about how you rate your general happiness. On a scale from 0 to 10 where 0 means "extremely unhappy" and 10 means "extremely happy"……

Overall, how happy are you?
- Enter no. ____________________
- Don't know

For peer review only - http://bmjopen.bmj.com/site/about/guidelines.xhtml

Mental Health
We also need to know some information about your mental and physical health. Please answer as best you can by selecting the best option.
Emotional Exhaustion

	Never	A few times a year or less	Once a month or less	A few times a month	Once a week	A few times a week	Every day
**103.** I feel emotionally drained from my work.	○	○	○	○	○	○	○
**104.** I feel used up at the end of the workday.	○	○	○	○	○	○	○
**105.** I feel tired when I get up in the morning and have to face another day on the job.	○	○	○	○	○	○	○
**106.** Working all day is a strain for me.	○	○	○	○	○	○	○
**107.** I feel burned out from my work.	○	○	○	○	○	○	○

Psychological Distress

	None of the time	A little of the time	Some of the time	Most of the time	All of the time
**108.** In the past four weeks, about how often did you feel tired out for no good reason?	○	○	○	○	○
**109.** In the past four weeks, about how often did you feel nervous?	○	○	○	○	○
**110.** In the past four weeks, about how often did you feel so nervous that nothing could calm you down?	○	○	○	○	○
**111.** In the past four weeks, about how often did you feel hopeless?	○	○	○	○	○
**112.** In the past four weeks, about how often did you feel restless or fidgety?	○	○	○	○	○
**113.** In the past four weeks, about how often did you feel so restless you could not sit still?	○	○	○	○	○
**114.** In the past four weeks, about how often did you feel depressed?	○	○	○	○	○
**115.** In the past four weeks, about how often did you feel everything was an effort?	○	○	○	○	○
**116.** In the past four weeks, about how often did you feel so sad that nothing could cheer you up?	○	○	○	○	○
**117.** In the past four weeks, about how often did you feel worthless?	○	○	○	○	○

For peer review only - http://bmjopen.bmj.com/site/about/guidelines.xhtml

**Depression**				
	**Not at all**	**Several days**	**More than half the days**	**Nearly every day**
**118.** During the last month, how often were you bothered by little interest or pleasure in doing things?	○	○	○	○
**119.** During the last month, how often were you bothered by feeling down, depressed, or hopeless?	○	○	○	○
**120.** During the last month, how often were you bothered by trouble falling or staying asleep OR sleeping too much?	○	○	○	○
**121.** During the last month, how often were you bothered by feeling tired or having little energy?	○	○	○	○
**122.** During the last month, how often were you bothered by poor appetite or overeating?	○	○	○	○
**123.** During the last month, how often were you bothered by feeling bad about yourself-or that you are a failure or have let yourself or your family down?	○	○	○	○
**124.** During the last month, how often were you bothered by trouble concentrating on things, such as reading a newspaper or watching television?	○	○	○	○
**125.** During the last month, how often were you bothered by moving or speaking so slowly that other people could have noticed OR – the opposite – being so fidgety or restless that you have been moving around a lot more than usual?	○	○	○	○
**126.** During the last month, how often were you bothered by thoughts that you would be better off dead or thoughts of hurting yourself in some way?	○	○	○	○

For peer review only

Stigma and Discrimination

127. In the last 12 months…
Do you think you have had emotional or mental health problems?
- ○ Yes (go to Q.128)
- ○ No (skip to Q.135)
- ○ Don't know (go to Q.135)

128. In the last 12 months…
Have you ever discussed your anxiety or depression with your supervisors and or managers?
- ○ Yes
- ○ No

129. Have you ever discussed your anxiety or depression with your co-worker?
- ○ Yes
- ○ No

	Not at all	A little	Moderately	A lot	Don't know
**130.** Have any of your supervisors and or managers avoided you because of the emotional or mental health problems?	○	○	○	○	○
**131.** Have any of your coworkers avoided you because of the emotional or mental health problems?	○	○	○	○	○
**132.** Have any of your supervisors and or managers treated you unfairly or discriminated against you because of your mental health problems?	○	○	○	○	○
**133.** Have any of your co-workers treated you unfairly or discriminated against you because of your mental health problems?	○	○	○	○	○
**134.** Because of your mental health or emotional problems have you been treated more positively by your employer?	○	○	○	○	○

For peer review only

For peer review only - http://bmjopen.bmj.com/site/about/guidelines.xhtml

Life Events

We now would like you to think about major events that have happened in your life over the past 12 months.

135. Did any of these events happen to you in the past 12 months? (please tick any event that applies to you)

- ☐ Death of spouse or child
- ☐ Separated from spouse or long-term partner
- ☐ Major worsening in financial situation (e.g., went bankrupt)
- ☐ Serious personal injury or illness to self
- ☐ Victim of physical violence (e.g., assault)
- ☐ Got married
- ☐ Partner or I gave birth to, or adopted, a new child
- ☐ Major improvement in financial situation (e.g., won lottery, received an inheritance)

136. Has a doctor ever diagnosed you with depression or anxiety?

- ○ Yes
- ○ No

137. Have you taken any prescription medications for your mental health in the last 12 months?

- ○ Yes
- ○ No
- ○ Don't know

138. Have you taken any of the following prescription medications in the past 12 months? (please tick any that apply to you)

- ☐ Sleeping tablets or capsules
- ☐ Tablets or capsules for anxiety or nerves
- ☐ Tranquillisers
- ☐ Antidepressants
- ☐ Mood stabilisers
- ☐ Other medications for your mental health
- ☐ None of the above
- ☐ Don't know

139. Have you taken this in the last month?

- ☐ Sleeping tablets or capsules
- ☐ Tablets or capsules for anxiety or nerves
- ☐ Tranquillisers
- ☐ Antidepressants
- ☐ Mood stabilisers
- ☐ Other medications for your mental health
- ☐ None of the above
- ☐ Don't know

For peer review only

For peer review only - http://bmjopen.bmj.com/site/about/guidelines.xhtml

Physical Health - General Health

140. During the past 7 days how much were you bothered by back or neck pain?
- Not at all
- A little
- Some
- A lot

141. During the past 7 days how much were you bothered by pain in your arms, legs, or joint areas like your knee or hips?
- Not at all
- A little
- Some
- A lot

142. During the past 7 days how much were you bothered by muscle soreness?
- Not at all
- A little
- Some
- A lot

143. In general, would you say your health is….?
- Excellent
- Very good
- Good
- Fair
- Poor

144. Have you had a significant physical injury from inside of work in the past 12 months?
- Yes
- No

145. Did you make a compensation claim?
- Yes
- No

Cardio-Vascular Health

146. In the past two years have you had your blood pressure taken by a health professional or used a self-administered machine?
- Yes
- No

IF yes, what was your blood pressure?
- Low
- Normal
- High
- Don't know

147. In the past two years have you consulted a health professional with regard to chest pain, or any other cardiovascular related health problem – such as myocardial infarction; angina; stroke; or hypertension?
- Yes
- No
- Don't know

IF yes, what was the diagnosis?
- Myocardial Infarction
- Angina
- Stroke
- Hypertension
- Other (specify)/Or if more than one, mention here _______________
- Don't know
- Not Stated
- Nothing

Work Outcomes - Job Satisfaction

148. Taking everything into consideration, how do you feel about your job as a whole?
- I'm extremely dissatisfied
- I'm very dissatisfied
- I'm moderately dissatisfied
- I'm not sure
- I'm moderately satisfied
- I'm very satisfied
- I'm extremely satisfied

Work Outcomes – Intention to Leave

149. Over this year I intend to leave this organisation.
- Strongly Agree
- Agree
- Neutral
- Disagree
- Strongly Disagree

For peer review only - http://bmjopen.bmj.com/site/about/guidelines.xhtml

Work Outcomes - Productivity

150. On a scale from 0 to 10 where 0 is the worst job performance anyone could have at your job and 10 is the performance of a top worker, how would you rate the usual performance of most workers in a job similar to yours?
Enter number here: ______________

151. Using the same 0-to-10 scale, how would you rate your overall job performance on the days you worked during the past 4 weeks (28 days)?
Enter number here: _______________

152. Using the same 0-to-10 scale, where 0 is no affect and 10 is the most affect, during the last 4 weeks (28 days) how much did your health problems affect your productivity at work?
Enter number here: __________________

Work Outcomes - Absenteeism

153. How many hours does your employer expect you to work in a typical 7-day week?
Note: If it varies, estimate the average. If more than 97, enter 97. If none - enter 0.
Enter hours: _______________________

154. In the past 4 weeks (28 days), how many days did you, miss an entire work day because of problems with your physical or mental health? (Please include only days missed for your own health not someone else's health?
Enter number of days (0-28): ________

155. About how many hours altogether did you work in the past 4 weeks?
Enter number of hours: _____________

Employment

The next questions are about your main job, that is the one in which you work most hours.

156. What is your occupation in this job – what are your main duties?
- ○ Managers or administrator (go to Q.157)
- ○ Professional work (go to Q.158)
- ○ Technician or associate professional (go to Q.159)
- ○ Tradesperson or related work (go to Q.160)
- ○ Advanced clerical, sales or service work (go to Q.161)
- ○ Intermediate clerical, sales or service work (go to Q.162)
- ○ Intermediate plant operator/transport (go to Q.163)
- ○ Elementary clerical, sales or service work (go to Q.164)
- ○ Labourer or related work (go to Q.165)
- ○ Other (please specify) _____________ (go to Q.166)

157. What best describes this work?
- ○ General Managers and Administrators
- ○ Miscellaneous Generalist Managers
- ○ Resource Managers
- ○ Engineering, Distribution and Process Managers
- ○ Sales and Marketing Managers
- ○ Miscellaneous Specialist Managers
- ○ Farmers and Farm Managers
- ○ Other (please specify) _____________ (go to Q.166)

158. What best describes this work?
- ○ Natural and Physical Science Professionals
- ○ Building and Engineering Professionals
- ○ Accountants, Auditors and Corporate Treasurers
- ○ Sales, Marketing and Advertising Professionals
- ○ Computing Professionals
- ○ Miscellaneous Business and Information Professionals
- ○ Medical Practitioners
- ○ Nursing Professionals
- ○ Miscellaneous Health Professionals
- ○ School Teachers
- ○ University and Vocational Education Teachers
- ○ Miscellaneous Education Professionals
- ○ Social Welfare Professionals
- ○ Miscellaneous Social Professionals
- ○ Artists and Related Professionals
- ○ Miscellaneous Professionals
- ○ Other (please specify) _____________ (go to Q.166)

For peer review only - http://bmjopen.bmj.com/site/about/guidelines.xhtml

159. What best describes this work?
- Medical and Science Technical Officers
- Building and Engineering Associate Professionals
- Finance Associate Professionals
- Miscellaneous Business and Administration Associate Professionals
- Shop Managers
- Hospitality and Accommodation Managers
- Miscellaneous Managing Supervisors (Sales and Service)
- Enrolled Nurses
- Welfare Associate Professionals
- Miscellaneous Health and Welfare Associate Professionals
- Police Officers
- Miscellaneous Associate Professionals
- Other (please specify) _______________ (go to Q.166)

160. What best describes this work?
- Mechanical Engineering Tradespersons
- Fabrication Engineering Tradespersons
- Automotive Tradespersons
- Electrical and Electronics Tradespersons
- Structural Construction Tradespersons
- Final Finishes Construction Tradespersons
- Plumbers
- Food Tradespersons
- Skilled Agricultural Workers
- Horticultural Tradespersons
- Printing Tradespersons
- Wood Tradespersons
- Hairdressers
- Textile, Clothing and Related Tradespersons
- Miscellaneous Tradespersons and Related Workers
- Other (please specify) _______________ (go to Q.166)

161. What best describes this work?
- Secretaries and Personal Assistants
- Advanced Numerical Clerks
- Miscellaneous Advanced Clerical and Service Workers
- Other (please specify) _______________ (go to Q.166)

162. What best describes this work?
- General Clerks
- Keyboard Operators
- Receptionists
- Intermediate Numerical Clerks
- Material Recording and Despatching Clerks
- Miscellaneous Intermediate Clerical Workers
- Intermediate Sales and Related Workers
- Carers and Aides
- Hospitality Workers
- Miscellaneous Intermediate Service Workers
- Other (please specify) _______________ (go to Q.166)

163. What best describes this work?
- Mobile Plant Operators
- Intermediate Stationary Plant Operators
- Intermediate Textile, Clothing and Related Machine Operators
- Miscellaneous Intermediate Machine Operators
- Road and Rail Transport Drivers
- Intermediate Mining and Construction Workers
- Miscellaneous Intermediate Production and Transport Workers
- Other (please specify) _______________ (go to Q.166)

164. What best describes this work?
- Elementary Clerks
- Sales Assistants
- Miscellaneous Elementary Sales Workers
- Elementary Service Workers
- Other (please specify) _______________ (go to Q.166)

165. What best describes this work?
- Cleaners
- Process Workers
- Product Packagers
- Mining, Construction and Related Labourers
- Agricultural and Horticultural Labourers
- Elementary Food Preparation and Related Workers
- Miscellaneous Labourers and Related Workers
- Other (please specify) _______________ (go to Q.166)

For peer review only

For peer review only - http://bmjopen.bmj.com/site/about/guidelines.xhtml

Now to finish with some general questions.

166. Do you identify as ….?
- ○ Male
- ○ Female
- ○ Non binary
- ○ Transgender Male
- ○ Transgender Female
- ○ Other (please specify): _______

167. Including yourself how many people aged 18 or over, who are currently in paid employment, live in this household?
Enter number: ______________________

168. How many children under 18 live in your household?
Enter number: ____________________

169. What state do you live in?
- ○ NSW
- ○ WA
- ○ SA
- ○ ACT
- ○ TAS
- ○ NT
- ○ QLD
- ○ VIC
- ○ Other: ________________

170. What is the postcode of the house?
____

171. What town or suburb do you live in?
Enter town/suburb: _______________

172. What state do you work in?
- ○ NSW
- ○ WA
- ○ SA
- ○ ACT
- ○ TAS
- ○ NT
- ○ QLD
- ○ VIC
- ○ Outside of Australia
- ○ Other (please specify): ____________

173. The Australian Council of Trade Unions (ACTU) in partnership with The University of South Australia (UniSA) and Employers Mutual Limited (EML) are co-hosting the Mind Your Head Campaign funded by WorkSafe Victoria's WorkWell Mental Health Improvement.
Have you heard/received any information about this campaign? (please disregard if not from VIC)
- ○ Yes
- ○ No

If 'Yes' what was the source of this information
- ○ Workplace communications
- ○ Media communications
- ○ External/personal communications
- ○ Other (please specify): ______________

174. What is your marital status?
- ○ Married
- ○ Living with partner
- ○ Separated
- ○ Divorced
- ○ Widowed
- ○ Never married

175. What best describes the highest educational qualification you have obtained?
- ○ Still at school
- ○ Left school at 16 years or less
- ○ Left school after age 16
- ○ Left school after age 16 but still studying
- ○ Trade / Apprenticeship
- ○ Certificate / Diploma
- ○ Bachelor degree or higher
- ○ Other (specify): ________________

176. We are interested to know if there is any relation to income levels and job stress risk. Before tax is taken out, which of the following best describes your income from your main job in the last 12 months?
- ○ Up to $12,000
- ○ $12,001 - $20,000
- ○ $20,001 - $30,000
- ○ $30,001 - $40,000
- ○ $40,001 - $50,000
- ○ $50,001 - $60,000
- ○ $60,001 - $80,000
- ○ $80,001 - $100,000
- ○ $100,001 - $150,000
- ○ $150,001 - $200,000
- ○ More than $200,000

For peer review only - http://bmjopen.bmj.com/site/about/guidelines.xhtml

In order for us to make really good use of your answers it would be of great benefit to ask you the same questions at a later date. Would you be willing to be re-contacted about doing this survey again in about a year's time? You are not obliged to say yes if we call. You can decide then.

177. Are you willing to be re-contacted?
- Yes (full name, please): _______________
- Best contact number: _______________
- Email: ______________________________
- No

178. Could we have your current address?
- Yes (please enter the address in below) ______________
- No

In this kind of research, it is important to group data so it better reflects the work situation rather than the individual situation. By using organisational names we can group data and link workplace data to other worker health related data bases. We can answer questions like "In general, do organisations with high work pressure have more worker injuries?"

179. Could you give the name of your organisation so we can do this? The organisation/employer will be confidential in any report.
Enter organisation or business name
____

180. What the suburb of your employer is?
Enter town/suburb: ________________

181. Is this the same organisation that you were at when we last interviewed you?
- Yes (skip to Q.183)
- No (go to Q.182)
- Can't remember (skip to Q.183)

182. If it is not the same organisation could we have the name and suburb of the organisation you worked at previously?
Enter organisation: _________________
Enter suburb: ______________________

We would also like to investigate how work conditions relate to mental health and related medications, such as "is work stress related to anti-depressant use in Australia?"
To facilitate this important research, we ask your permission to securely send your AWB personal information (along with other participant's information) to the Australian Institute of Health and Welfare to match to the Pharmaceutical Benefits Scheme data to create an anonymous research dataset.

183. Do you consent for us to securely send your personal AWB information (the information you have provided in this survey) to the Australian Institute of Health and Welfare to match to medication data in the Pharmaceutical Benefits Scheme and create an anonymous research dataset?
- Yes
- No

That was the final question. Thank you again for your time and support for the Australian Workplace Barometer project. We really appreciate it.

During this survey you were to think of some negative and positive experiences. You have also been asked some questions about if you have experienced any levels of depression. If you feel distressed as a result of any of these questions we offer you the telephone numbers below, should you need to discuss some of these concerns with a qualified professional.

Beyond Blue Support Service Line: 1300 224 636
Lifeline: 13 11 14

For peer review only

BMJ Open

Psychosocial safety climate (PSC) and working conditions, predictors of mental health and antidepressant and opioid use in Australia: A study protocol for longitudinal data linkage.

Journal:	*BMJ Open*
Manuscript ID	bmjopen-2023-074235.R2
Article Type:	Protocol
Date Submitted by the Author:	23-Nov-2023
Complete List of Authors:	Crispin, Cherie; University of South Australia, Justice & Society<br>Afsharian, Ali; University of South Australia, Justice & Society<br>Loh, May; University of South Australia, Justice & Society<br>Dollard, Maureen F.; University of South Australia,<br>Dormann, Christian; Johannes Gutenberg Universitat Mainz<br>Glozier, Nick; University of Sydney Brain and Mind Research Institute,<br>Gill, Tiffany; The University of Adelaide, Medical Specialities<br>Taylor, Anne; The University of Adelaide, School of Medicine
**Primary Subject Heading**:	Mental health
Secondary Subject Heading:	Epidemiology, Occupational and environmental medicine, Public health, Research methods
Keywords:	OCCUPATIONAL & INDUSTRIAL MEDICINE, EPIDEMIOLOGY, Health & safety < HEALTH SERVICES ADMINISTRATION & MANAGEMENT, Health policy < HEALTH SERVICES ADMINISTRATION & MANAGEMENT, MENTAL HEALTH, PUBLIC HEALTH

SCHOLARONE™
Manuscripts

Title:

Psychosocial safety climate (PSC) and working conditions, predictors of mental health and antidepressant and opioid use in Australia: A study protocol for longitudinal data linkage.

Abstract:

Introduction: Work-related stress is a social determinant of global health [1] that represents a huge cost to workers' health and reduces work performance [2]. In Australia mental wellbeing is a pressing national issue – with one in five Australians experiencing mental disorders [4]. Anti-depressants are a first-line medication commonly used to treat mental disorders [6]. Recently, Australia has seen a dramatic increase in the use of prescribed antidepressant medications to treat mental health related illnesses [8]. Australia has also seen a dramatic increase in the use of prescribed opioid analgesics for noncancer pain including opioid use for psychological distress and social stressors [9-10]. It is plausible a rise in mental health problems and antidepressant and opioid medication is partly attributable to the corporate climate for worker mental health (i.e., the psychosocial safety climate, PSC). This research aims to identify how PSC and workplace conditions contribute to employee wellbeing and distress that culminate in antidepressant and opioid medication use.

Methods/Analysis: Data will be collected through creative data linkage from the Australian Workplace Barometer (AWB) [30], to medication data (via the Pharmaceutical Benefits Scheme, PBS). The participant sample will include 1,372 working Australians from the AWB project from 2009 to 2021. Four waves of longitudinal data from 2009 to 2021 will be used to investigate the plausible link between Australia's high levels of antidepressant and opioid use and distress at work. The project advances theory by probing the role corporate climate plays in work design, distress, mental health problems and antidepressant and opioid use. It will determine if antidepressant and opioid use has led to an underestimation of work stress effects. Proposed theoretical models will be analysed through linked data, using continuous time structural equation modelling (SEM), hierarchical linear modelling (HLM), logistic regression and cost estimation.

Ethics and dissemination:
The study has been approved by the Human Research Ethics Committee of the University of South Australia (Ethics Protocol: 203003). Further, approval from the Australian Institute of Health and Welfare Ethics Committee was also granted for linkage of AWB data and PBS data (EthOS Application EO2022/1/1190).

Results of the study will be disseminated through worldwide keynotes, key international settings, high-impact peer-reviewed journals, industry conference presentations, and media outlets to reach managers, workers and industry partners. Further, UniSA requires publications from public projects to be held in an institutional repository which fulfils the Australian Research Council's Open Access Policy.

Strengths and Limitations:

- The longitudinal linkage of both data sources enables information to be chronologically ordered to provide valuable insight otherwise missing from single source cross-sectional data.
- Data linkage will overcome common method bias associated with self-report research of mental health, through validation with objective measures.
- Data linkage success is largely dependent on specific individual participant characteristics (e.g. first name, surname, date of birth) being provided at the point of interview. As this information was limited this restricted a more robust data linkage.

For peer review only

- Due to the nature of data privacy, the data linkage process can be prolonged due to the development of technical documentation pertaining to specific use of the data, the provision of a secure workspace with encrypted data and strict personnel access, as well as the rigorous process of ensuring all identifiable data is removed from both data sources.

Request permissions:

This is an Open Access article distributed in accordance with the Creative Commons Attribution Non-Commercial (CC BY-NC 4.0) license, which permits others to distribute, remix, adapt, build upon this work non-commercially, and license their derivative works on different terms, provided the original work is properly cited and the use is non-commercial. See: http://creativecommons.org/licenses/by-nc/4.0/
If you wish to reuse any or all of this article please use the link below which will take you to the Copyright Clearance Center's RightsLink service. You will be able to get a quick price and instant permission to reuse the content in many different ways.

Data Availability Statement: All data relevant to the study are included in this study protocol. The Australian Workplace Barometer Project Dataset was collected from 2009 to 2015 (Wave 1-3 inclusive) and is available through the Australian National University Library. Pharmaceutical Benefit Scheme data is not available for sharing.

Registration details: In the interests of transparency, reproducibility, and reducing publication bias across the wider scientific community, the present study has been pre-registered on the Open Science Framework (OSF Registries) DOI: XX XX XX

Keywords: Psychosocial safety climate, organisational climate, antidepressant use, opioid use, work stress, mental health

Background:

Work-related stress is a social determinant of global health [1] that represents a huge cost to workers' health and reduces work performance [2]. Every year an estimated 12 billion working days are lost to depression and anxiety, with approximately 15% of working-age adults worldwide reported to have a mental disorder in 2019 [3]. In Australia alone, one in five Australians are experiencing mental disorders [4] with problems related to employment the most common risk factors of suicide in the working age population [5].
A common form of treatment for mental disorders is antidepressant medication [6]. Epidemiological studies have demonstrated an increase in usage of antidepressant prescriptions in industrialised countries during the 21st century [6,7]. Since 2017, Australia has seen a dramatic increase in the use of prescribed antidepressant medications to treat mental health related illnesses [8]. The Australian Institute of Health and Welfare (AIHW) reported that in 2020-21, 73.1% of mental health-related prescriptions were filled for anti-depressant medications [8]. Additionally, over the past two decades, Australia has seen a dramatic increase in the use of prescribed opioid analgesics for noncancer pain including opioid use for psychological distress and social stressors [9-10]. According to the AIHW [11], in 2019 opioid use within Australia (3.3% of the adult population) remained higher than the global average (1.2% of the global population aged 15-64 years). It is plausible a rise in mental health problems and antidepressant and opioid medication is partly attributable to the corporate climate. Indeed, only 52% of employees across Australia think their workplace positively supports their mental wellbeing [12], and only 9% of employers have an "integrated and sustained approach to mental health and wellbeing" [13]. Work pressure and bullying - products of poor management - account for almost 7,500 workers compensation claims per annum for work-related mental disorders [14].

During the past few decades, a multitude of studies have been published on working conditions and mental health and prescribed medication use [15-17]. Yet the theory and evidence linking

For peer review only - http://bmjopen.bmj.com/site/about/guidelines.xhtml

work to mental ill-health and medication use are incomplete in significant ways. Firstly, studies largely focus on work design, such as the levels of job demands (e.g., work pressure, emotional demands such as bullying) and resources (e.g., job control, social support) and their combinations as precursors to worker ill health and increased risk of depressive symptoms [16]. Although there is a strong theoretical (Job Demand-Control-Support - DCS) [18]; and the Effort-Reward Imbalance - ERI – model [19]) and empirical base linking work design to mental health problems [16, 20-21], prominent researchers are now going further and asking, “Where do work design and work conditions come from?” [22]. Dollard & colleagues [23-25] believe that the root cause emanates further upstream, that is with the corporate climate or Psychosocial Safety Climate (PSC).

PSC theory is an innovation in the field [23-25], defined as ‘policies, practices and procedures for the protection of worker psychological health and safety’ [23, p. 579]. It refers to management commitment and priority, organisational communication, and organisational participation and involvement, specifically in relation to worker psychological health and safety. PSC theory has gained prominence nationally and internationally and is a unifying construct that has promoted interdisciplinarity integrating work stress and safety science research. The construct is empirically distinct from related constructs such as team psychological safety, perceived organisational support, and safety climate [26]. Whereas the safety climate construct is used to predict safety behaviour and accidents and injuries [27], PSC instead is used to predict psychosocial work design and psychological health and is also linked to the enactment of safety behaviours and the reporting of workplace injuries [28] .

Work design theories are nevertheless important to explain the process and why PSC relates to mental health outcomes. A comprehensive work design framework such as the Job Demands-Resources model [29], is required that integrates the components of ERI and DCS models, by encompassing various kinds of demands and resources in explanation of mental health and engagement. Work design is influenced by the organisational priorities and policies. T tying PSC to work design theories (e.g., the JD-R model), whereby PSC is the predictor of the JDR model) is likely a feasible explanatory framework to understand the relationship between work conditions associated with work stress reactions and mental health problems linked to antidepressant and opioid medication use. The PSC extended JDR theory is the backdrop for this study.

This study will yield evidence to stimulate corporate climate change to protect workers' psychological health and well-being. The Australian Workplace Barometer (AWB) has connected work factors to self-reported mental ill-health [30]. However, it has not yet been investigated whether these contribute to the high levels and costs of antidepressant use. Moreover, it is unknown whether the large consumption of antidepressants by Australians is masking a more urgent work stress problem by reducing symptoms leading to underestimation in studies. Uncovering the 'true mental effects' of stressful work is essential.

Aims

This research aims to identify how the corporate climate and workplace conditions contribute to Australian employee wellbeing and distress that culminate in them using antidepressant and opioid medications. This study will investigate the plausible link between distress at work and Australia's high levels of antidepressant and opioid use through the creative linkage of data from the Australian Workplace Barometer (10-year longitudinal study) to antidepressant and opioid medication data (via the data collected by Australian national Pharmaceutical Benefits Scheme (which contains information on prescribed medicines that qualify for medical claims). The project advances theory by probing corporate climate's role in work design, distress, mental health problems, stigma/discrimination and antidepressant and opioid use. It will determine if antidepressant and opioid use has led to underestimating work stress effects. Data will also be used to estimate the $AUD cost of work-related antidepressant and opioid use.

Research Questions

For peer review only

Research Question 1:

The main research question is 'Does Psychosocial Safety Climate have a primary role, in process pathways linking working conditions to mental health problems and antidepressant/opioid medication use?'

Research Question 2:

What is the cost (in $AUD) of antidepressant/opioid medication use related to work factors?

Research Question 3:

Is there a positive relationship between Australian employees' experience of stressful work conditions (e.g., high demands, low resources, bullying, harassment, stigma, discrimination) and antidepressant and opioid medication use.

Research Question 4:

Is there a positive relationship between Australian employees' experience of distress, emotional exhaustion and depressive symptoms and antidepressant and opioid medication use?

Research Question 5:

Does antidepressant/opioid use lead to an underestimation of the impact of stressful work on employees?

This research will include two separate studies;

Study 1; will look at corporate climate, work stressors, mental health, and *antidepressant* medications

Study 2: will look at corporate climate, work stressors, mental health, and *opioid* medications

Methods & Analysis:

Study Design:

This study will use a longitudinal design that consists of four waves of interview and self-report data using an existing data set, the Australian Workplace Barometer (AWB), a population-based longitudinal data set, matching individuals over time and linking to objective medication data (Pharmaceutical Benefits Scheme, PBS). The four waves of data from the AWB dataset include data collected in 2009-2010 (Wave 1), 2010-2011 (Wave 2), 2014-2015 (Wave 3) and 2020-2021 (Wave 4). The AWB cohort data will be linked to PBS data collected from 01/01/2009 to the latest available data. The different time points between data collection will help track the progression of effects across different time periods. The current study will commence in May 2023 and run until December 2023.

Explanation of existing data

As of the submission date, the data exists, and researchers have only had access to the AWB data. However, no detailed analysis has been conducted concerning the current research plan (including the calculation of summary statistics). Access to the AWB data has been limited, though a small portion of the data has been used to conduct exploratory analysis that showed encouraging initial results. PBS data has not been accessed at the point of registration.

Data collection procedures

In the first round of data collection, The Australian Workplace Barometer (AWB) used a population-based random sampling approach to conduct computer-assisted telephone interviews (CATI). Participants were recruited randomly from the Australian electronic white pages inviting

household members to participate (the participant had to have had the last birthday in the household to be eligible).

For each subsequent wave of data collection, those who had consented to be re-contacted were sent a letter advising of further data collection and re-contact for a further interview. A combination of modes was used to collect subsequent waves of AWB data to increase sample representativeness and participation uptake, including telephone interviews by landline or mobile, surveys via email or SMS link, and hard copy surveys sent via post. Where participants could not partake in interviews at the first point of contact, call-backs for a more suitable time were arranged.

Telephone interviews were conducted by a team of research assistants trained to enter data immediately into the Qualtrics online survey. Participants who received an email or SMS link were provided with their participant ID (required for linkage) and entered their participant ID and self-reported data directly into the Qualtrics survey link provided. Hard copy surveys were entered manually into the Qualtrics online survey by the research team upon receipt.

Detailed information on survey items used in the study are uploaded as supplementary information (see online supplemental S1).

Payment for Participation:

In the fourth wave of data collection, participants who completed the survey automatically went into the draw to receive a gift card valued at $100. The winner of the draw was picked using a random number generator, contacted by the research team's chief investigator by phone, and announced the winner.

Inclusion criteria:

Participants are required to be a minimum of 18years of age and currently in paid employment. Sub-contractors must have worked with the same employer for a minimum of 4 months. Participants must have consented to their personal AWB information being securely sent to the Australian Institute of Health and Welfare (AIHW) to match the Pharmaceutical Benefits Scheme (PBS) dataset for research purposes.

Exclusion criteria:

Ages out of the age range, unemployed, and those who did not consent to data linkage are excluded from the dataset.

Pharmaceutical Benefits Scheme (PBS) Dataset:

National Medicare & PBS will provide data on prescription medicines, defined by the Australian Institute of Health and Welfare (AIHW) project as antidepressants to treat clinical depression and opioid medication use for this project. The Pharmaceutical Benefits Scheme (PBS) data comprises individual antidepressant and opioid medication use (summed over a year) from 2009 to 2021.

Data Linkage (AWB linkage to PBS):

PBS data was linked via AWB participants who consented to have their AWB data linked to PBS data in 2020/2021. Antidepressant medication will be matched with the AWB at the individual level, using individual names and addresses.

Data Linkage Procedure:

Step 1: A 'Request for linkage of national data: technical assessment' was completed by the second author as part of the AIHW data linkage protocol and AIHW ethics approval, which outlined the proposed research and study aims.

Step 2: AIHW was sent two files via secure email, including:

- AWB cohort personal identifiers with a data ID code
- AWB content data and with the data ID code

Step 3: AIHW linked the AWB cohort to PBS. This linkage process included the following;

- Creation of a new project-specific person number
- Loading the PBS content data and the AWB content data to the SURE project-specific person number
- All data transfers were via secure messenger service.

Important Data Linkage Notes:

- The technical assessment is a rigorous process required by AIHW to ensure that the data requested for the linkage answer the proposed research questions. The technical assessment ensures the study aims are achievable using the proposed linkage methodology and forms part of the AIHW ethics approval for the research project.
- AIHW set up a specific project ID for the linkage of the AWB and PBS data and the provision of a secure workspace, referred to as the Secure Unified Research Environment (SURE) workspace, for the analysis of linked data. All analytical software packages (SPSS, R) are loaded onto the SURE secure workspace. Deidentified AWB data set and PBS data are curated into the secure workspace.
- There are strict protocols in place during SURE access, where the original AWB dataset is held on a secure university server that restricts researcher access involved with SURE data analysis. This highly confidential workspace is constructed to hold data that blocks all access to other desktop files and software to avoid identifying deidentified participants.
- A research Assistant (the first author) will be the data custodian (not involved in the data analysis) and will be appointed during this time to ensure no local data access of identifiable/ re-identifiable data while there is access to SURE data. All data analysis takes place with the data on the secure SURE website.

Sample size

The sample included 1372 working Australians who participated in the AWB study from 2009 to 2021 and consented to the data linkage. Participants are located across all Australian states and territories; South Australia (SA), New South Wales (NSW), Western Australia (WA), Tasmania, Australian Capital Territory (ACT), and the Northern Territory (NT).

Patient and public involvement

There was no patient or public involvement in the design and development of the study.

Measured variables

All independent and dependent variables are measured at the individual level.

Demographics and workplace information:

Demographic variables in the current study include age, gender, industry, education and income. Work-related information includes work hours (i.e. shift work), length of service, working status (employed vs. unemployed), and employment status (permanent full-time, permanent part-time, casual, and fixed term contract), occupation, size of organisation, organisation business type (i.e. state government, not-for-profit) name of current organisation (optional), and lodgement of workers compensation claims.

Psychosocial Safety Climate:

The psychosocial safety climate was measured using a scale consisting of 12 items (PSC-12) [31] that assess four dimensions of PSC, including management priority, management commitment, organizational communication, and organizational participation. Items were measured on a 5-point Likert scale, ranging from 1 (strongly disagree) to 5 (strongly agree). Scores for all 12 items are summed to provide an overall score for PSC. An example item is, “In my workplace, senior management acts quickly to correct problems/issues that affect employees’ psychological health”.

Psychosocial Demands:

1. Work pressure

Work pressure was measured using the 5-item psychological job demands scale from the Job Content Questionnaire (JCQ 2.0) [32], an example item “My job requires working very fast”. Items were measured on a 4-point Likert scale, ranging from 1 (strongly disagree) to 4 (strongly agree). The five items were summed to give a total score for the work pressure.

2. Workplace bullying:

Bullying was assessed using an amended version of the QPSNordic Bullying Questionnaire [33]. Participants were provided with a definition of workplace bullying and then answered questions about their exposure to bullying in the workplace.

Definition provided to participants; “Bullying is a problem at some workplaces and for some workers. To label something, as bullying, the offensive behaviour has to occur repeatedly over a period of time, and the person confronted has to experience difficulties defending him or herself. The behaviour is not bullying if two parties of approximate equal ‘strength’ are in conflict or the incident is an isolated event” [34].

Participants were then asked, “Have you been subjected to bullying at the workplace during the last six months?” Those who replied with ‘yes’, were then asked by whom (supervisor/co-worker) and the length and frequency of exposure to workplace bullying.

3. Workplace harassment

Seven items from Richman et al.’s (1996) scale [35] were used to measure workplace harassment. Items were measured on a 5-point Likert scale, ranging from 1 (very rarely/never) to 5 (very/often always) and summed together to represent a total for workplace harassment. An example item is “I have been sworn at and/or yelled at”.

Psychosocial demands (work pressure, workplace bullying & workplace harassment) are independent variables., all measured longitudinally.

Psychosocial Resources

1. Job control

Three subscales were used from the JCQ 2.0 [32] to measure job control, including the 5-item skill discretion subscale ("My job requires that I learn new things"), the 3-item decision authority subscale ("In my job I have very little freedom to decide how I do my work") and the 3-item macro-decision latitude subscale ("In my organization, I have significant influence over decisions made by my work team and department"). All 12 items were measured on a 4-point Likert scale, ranging from 1 (strongly disagree) to 4 (strongly agree). Items were summed to provide a total for job control.

2. Social Support

To measure social support, six items from the JCQ 2.0 [32] was used, three items for supervisor social support ("My supervisor/manager is helpful in getting the job done") and three items for co-worker support ("People I work with are friendly"). Items were measured on a 4-point Likert scale, ranging from 1 (strongly disagree) to 4 (strongly agree) and summed for both supervisor and co-worker to provide a total score for each social support scale.

Job control (skill discretion, decision authority & macro-decision latitude) and social support (supervisor and co-worker) are independent variables, measured longitudinally.

Distress Symptoms

1. Psychological distress

The Kessler 10 scale [36] was used to measure psychological distress. A 5-point Likert scale measured responses, ranging from 1 (none of the time) to 5 (all of the time). Scores on the ten

items were totalled to give an overall score on psychological distress. An example item is "[In the past four weeks], about how often did you feel tired out for no good reason?".

2. Emotional exhaustion

Five items from the Maslach Burnout Inventory [37] were used to measure emotional exhaustion on a 7-point Likert scale, ranging from 1 (never) to 7 (every day). An example item is, "I feel burned out from my work."

Distress symptoms and/or mental health problems are dependent variables measured longitudinally (four-time points) using psychological distress and emotional exhaustion.

Work Engagement

Work engagement was measured using three items from the Utrecht Work Engagement Scale – Shortened Version (UWES-9) [38], covering vigour, dedication and absorption. Items were measured on a 7-point Likert scale, ranging from 1 (never) to 7 (every day) and summed together to provide an overall score for work engagement. An example item is "At work I feel bursting with energy".

Engagement will be used as a dependent variable, measured longitudinally.

Stigma/Discrimination

Stigma/discrimination was measured using one item from the Kessler 6 (K6) mental health symptom screening questionnaire ("In the last 12 months, do you think you have had emotional or mental health problems?"). If participants answered 'yes', a further seven questions were asked about their experiences within the workplace of discrimination ("Have any of your supervisors and or managers treated you unfairly or discriminated against you because of the mental health problems?"), avoidance ("Have any of your supervisors and or managers avoided you because of the emotional or mental health problems?") and positive treatment ("Because of your mental health or emotional problems have you been treated more positively by your employer?") [39].

Stigma/discrimination was only measured in wave 4 (2020/2021).

Mental Health Problems

1. Depression

All nine items from the Patient Health Questionnaire (PHQ-9) [40] were used to measure depressive symptoms in the present study, with a time reference modified for the last four weeks, an example item "[During the last month, how often were you bothered by] feeling down, depressed, or hopeless?". Items were measured on a 4-point Likert scale, from 1 (not all) to 4 (nearly every day). Scores were totalled to give an overall score on depression.

2. Suicidal ideation

One item from the Patient Health Questionnaire (PHQ-9) [40] was used to measure suicidal ideation ("[During the last month, how often were you bothered by] thoughts that you would be better off dead or thoughts of hurting yourself in some way?").

Depression and suicidal ideation are dependent variables, measured longitudinally (four-time points).

Prescribed Medication Usage

1. Self-report prescribed medication usage data

Participants were asked the question, "Have you taken any prescription medications for your mental health in the last 12 months?". Those who answered 'yes' were then asked to indicate from a list of prescribed medications (sleeping tablets/capsules, tablets/capsules for anxiety or nerves, tranquillizers, antidepressants, mood stabilizers, and other medications for your mental

For peer review only - http://bmjopen.bmj.com/site/about/guidelines.xhtml

health) which medications they had used in the last 12 months, and also in the last four weeks. Antidepressant medication is a dependent variable measured only in wave 4 (2020/2021).

2. Registered PBS Medication Data

PBS variables include PBS item, drug type, benefit amount, the patient contribution amount, under co-payment prescription type, prescribed speciality, prescriber type, date of prescribing, number of scripts dispensed, quantity supplied, date of supply, and patient category.

Supplemental Material:

S1. Australian Workplace Barometer (AWB) Survey for Wave 4 (2020/2021)

Data Analysis Plan:

The proposed theoretical models will be analysed using data linked from the AWB and PBS, mainly using ANOVA, structural equation modelling (SEM), hierarchical linear modelling (HLM), where applicable, and logistic regression.

ANOVA

By using the depression score over time, we will categorise the sample into four categories, i.e., depressed, recovering, worsening, and healthy to capture the changes in their mental health condition over ten years. We will conduct ANOVA to identify the differences in their reported corporate climate and working conditions.

Structural Equation Modeling (SEM) will be used with time-lagged effects to estimate the direction and strength of the hypothesised relationships between work factors, stress reactions, and medication use. We will evaluate model fit using indices like chi-squared tests, RMSEA, CFI, and TLI. With time-lagged SEM, we will be able to depict how variables and their associated relationships with other variables change over time (e.g., how a change in PSC would influence the change in distress reactions in the future). To better handle the varied time intervals in the AWB data, we will use continuous time SEM (CTSEM). CTSEM, with a Bayesian approach, allows individual-varying specifications for time intervals and other parameters in the analysis, addressing potential issues due to the unequal intervals between measurements and providing inferences on continuous processes over time. We will also estimate within-person and between-person effects simultaneously by modelling both observed covariates and unobserved heterogeneity to gain a more nuanced understanding of the tranquilising effects of medication on work stress and the influence of PSC in the process pathway.

Hierarchical Linear Modeling (HLM) will be used to account for hierarchical data structures, such as individuals nested within organisations (if the data allows) or multiple occasions within individuals. Using HLM, we will investigate how work factors and stress reactions vary at both the individual and organisation levels, and their impact on medication use.

Logistic regression will be conducted for any potential binary classification problems, such as the use of medication predicting whether workers will use medication (yes/no) based on the levels of work stress or poor organisational climate they are exposed. Odds ratios will be calculated to estimate the risk level of exposure to poor work conditions and low levels of PSC.

Cost estimation

The cost associated with mental health issues and medication use attributable to work-related factors will be calculated by the total cost of medication usage and medical compensation claims through PBS data and the attributable fractions (AFs) of poor work condition, PSC, and work

stress. AFs can be estimated, for example, by using an empirical established threshold, the PSC benchmark [41] to identify the proportion of exposed and unexposed workers to poor PSC and work condition.

Exploratory Analysis Plan

We will investigate the role of stigma/discrimination and PSC for workers experiencing work stress/distress symptoms and their association with anti-depressant and opioid medication use to determine whether the corporate climate tranquillizes the effects of work stress and anti-depressant and opioid use and whether stigma moderates these effects. We will explore mental health vulnerability as a predictor by assessing a range of indicative states suggested by mental health self-reports. Instead of controlling for this as prior research [42], we will investigate its trajectory, subsequent stressor exposure (e.g., bullying), and future mental health status. This has implications for how mental health is managed in the workplace. We will also investigate the validity of self-report medication use data against PBS data (registered data).

Ethics & Dissemination:

The study has been reviewed and approved by the Human Research Ethics Committee of the University of South Australia (Ethics Protocol: 203003). Further, approval from the Australian Institute of Health and Welfare Ethics Committee was also granted for the linkage of AWB data and PBS data (EthOS Application EO2022/1/1190).

Results of the study will be disseminated through worldwide keynotes, key international settings such as the Joint Congress of ICOH-WOPS, high-impact peer-reviewed journals, industry conference presentations, and various media outlets (such as LinkedIn, Twitter, radio, community forums) to reach managers, workers and industry partners. Further, UniSA requires publications from public projects to be held in an institutional repository which fulfils the Australian Research Council's Open Access Policy. Therefore, we will also lodge relevant outputs with the Analysis and Policy Observatory collection for greater accessibility.

Author Contributions:

Cherie Crispin : Ms Crispin was involved in collection of longitudinal data since 2020. She will assist with preparation and drafting of study papers, ethics applications, development of study protocol, development of model design and theoretical framework for Study Two (corporate climate, work stressors, mental health and *opioid* medications), provision of AWB data sets for data linkage agencies, liaison with data linkage agencies.

Dr Ali Afsharian : Dr Afsharian was involved in collection of longitudinal data since 2020. He will assist with ethics applications, progress reports, drafting papers and study protocol, development of methodology, model design and theoretical framework for Study One (corporate climate, work stressors, mental health, and *antidepressant* medications), liaise with data linkage agencies and will be one of three researcher team members to perform computations and statistical analysis.

Dr May Young Loh : Dr Loh will assist with theory development, methodology and model design for Studies One and Two and will be one of three research team members to perform computations and complex statistical analysis.

Professor Maureen Dollard : Prof Dollard conceived of the idea, developed and contributed to the original design and implementation of the research. Prof Dollard will lead, supervise and coordinate the research team and take overall responsibility for the strategic intellectual direction of the project and communication of results. Further, she will assist with theory development, methodology and model design for Studies One and Two and will be one of three research team members to perform computations and statistical analysis.

For peer review only

Professor Christian Dormann : Prof Dormann will assist the team in integrating and testing new societal cost elements of Psychosocial Safety Climate (PSC) theory using R, structural equational modelling, and continuous time modelling, and contribute to publication of results.

Dr Nick Glozier : Dr Glozier will be responsible for aspects of data linkage, mental health and health service evaluation, and PBS medication interpretation and will contribute to the costing and epidemiological aspects of the study design.

Dr Tiffany Gill : Dr Gill is an epidemiologist who was involved in the original development and implementation of the AWB surveys in 2009. She will provide advice on survey, ethical and other project related issues as required.

Professor Anne Taylor : Prof Taylor will assist with expert advice on interpretation of data analysis and data-linkage. Prof Taylor was originally involved with sampling, the set-up of Computer Assisted Telephone Interviewing (CATI) and data collection. She will assist with the review of papers where required.

All authors have reviewed and approved the final version of the study protocol.

Funding Statement:

This work was supported by five grants: (1) Australian Research Council (ARC) DP200102752 (103319) Dollard M.F. (2) DP087900 Australian Research Council Discovery Grant. Dollard MF, Winefield AH, LaMontagne AD, Taylor AW, Bakker AB, Mustard C. Working wounded or engaged? Australian work conditions and consequences through the lens of the job demands resources model. (3) LP100100449 Australian Research Council Linkage Grant. Dollard MF, Winefield AH, Taylor AW, Smith PM, Nafalski A, Bakker A, Dormann C. State, organisational, and team interventions to build a psychosocial safety climate using the Australian Workplace Barometer and the StressCafé. (4) FL200100025 Australian Research Council Laureate Fellowship. (5) Safework NSW's Centre for WHS.

Competing interests' statement.

None declared

References:

1. World Health Organisation (WHO). Closing the gap in a generation: Health equity through action on the social determinants of health [online]. 2008. https://www.who.int/publications/i/item/WHO-IER-CSDH-08.1 (accessed on 14 March 2023).
2. International Labour Organisation (ILO). World Employment and Social Outlook: Trends 2016 [online]. 2016. https://www.ilo.org/wcmsp5/groups/public/@dgreports/@dcomm/@publ/documents/publication/wcms_443480.pdf (accessed on 14 March 2023).
3. World Health Organisation (WHO). Mental health at work [online]. 2022. https://www.who.int/news-room/fact-sheets/detail/mental-health-at-work
4. Australian Bureau of Statistics. National study of mental health and wellbeing [online] 2022. https://www.abs.gov.au/statistics/health/mental-health/national-study-mental-health-and-wellbeing/latest-release (accessed 28 February 2023).
5. Australian Bureau of Statistics. Causes of death, Australia [online]. 2022. https://www.abs.gov.au/statistics/health/causes-death/causes-death-australia/latest-release (accessed 28 February 2023).
6. Lunghi, C., Dugas, M., Leclerc, J., et al. (2022). Global prevalence of antidepressant drug utilization in the community: protocol for a systematic review. BMJ Open. http://doi.org/10.1136/bmjopen-2022-062197

For peer review only

7. Whiteford, H.A., Degenhardt, L., Rehm, J., et al. (2013). Global burden of disease attributable to mental and substance use disorders: findings from the Global Burden of Disease Study 2010. Lancet 382, 1575–1586. https://doi.org/10.1016/S0140-6736(13)61611-6
8. Australian Institute of Health and Welfare. Mental Health Prescriptions [online]. 2022. https://www.aihw.gov.au/mental-health/topic-areas/mental-health-prescriptions (accessed 28 February 2023).
9. Lalic, S., Ilomaki, J., Bell, J. S., et al. (2019). Prevalence and incidence of prescription opioid analgesic use in Australia. British Journal of Clinical Pharmacology, 85, 202-215. https://doi.org/10.1111/bcp.13792
10. Wilson, J., Lam, T., Scott., D., et al. (2020). 'Extreme personal stress' and 'a lot of pain': Exploring the physical, mental and social contexts of extramedical pharmaceutical opioid use in Australia paramedic case descriptions. Drug and Alcohol Review, *39,* 870-878. https://doi.org/10.1111/dar.13118
11. Australian Institute of Health and Welfare. Opioid harm in Australia; and comparisons between Australia and Canada [online]. 2018. https://www.aihw.gov.au/getmedia/605a6cf8-6e53-488e-ac6e-925e9086df33/aihw-hse-210.pdf.aspx?inline=true (accessed on 28 February 2023).
12. beyondblue. The State of Workplace Mental Health in Australia [online]. 2015. https://www.headsup.org.au/docs (accessed on 28 February 2023).
13. Safe Work NSW. Mentally healthy workplaces in NSW [online]. 2017. https://www.safework.nsw.gov.au/__data/assets/pdf_file/0018/320139/Mentally-healthy-workplaces-in-NSW-benchmarking-tool-October-2017-SW08734.pdf (accessed on 14 March 2023).
14. Safe Work Australia. Compendium of Workers' Compensation Statistics Australia 2015-16 [online]. 2017b. https://www.safeworkaustralia.gov.au (accessed on 14 March 2023).
15. Lahti, J., Lallukka, T., Harkko, J., et al. (2021). Working conditions and antidepressant medication use: A prospective study among 18 to 39-year-old municipal employees. Psychiatry Research, 305, 114213, https://doi.org/10.1016/j.psychres.2021.114213
16. Theorell, T., Hammarstrom, A., Aronsson, G., et al. (2015). A systematic review/ meta-analysis of work environment and depressive symptoms. BMC Public Health, 15, 738. https://doi.org/10.1186/s12889-015-1954-4
17. Woo, J.M., Postolache, T.T., 2008. The impact of work environment on mood disorders and suicide: Evidence and implications. International Journal of Disability. Human Development. 7, 185–200. https://doi.org/10.1515/ijdhd.2008.7.2.185
18. Karasek, R. & Theorell, T. (1990). Healthy Work. New York: Basic Books
19. Siegrist, J (1996). Adverse health effects of high-efforts/low-reward conditions. Journal of Occupational Health Psychology,1, 27–41. https://doi.org/10.1037//1076-8998.1.1.27
20. Leach, LS., Poyser, C. & Butterworth. (2016). Workplace bullying and the association with suicidal ideation/thoughts and behaviour: a systematic review. Occupational and Environmental Medicine, 74(1). https://doi.org/10.1136/oemed-2016-103726
21. Loerbroks, A., Cho, S., Dollard, M.F., et al. (2016). Associations between work stress and suicidal ideation. Journal of Psychosomatic Research, 90, 62–69. https://doi.org/10.1016/j.jpsychores.2016.09.008
22. Parker, SK, Morgeson, FP, & Johns, G. (2017). One hundred years of work design research. Journal of Applied Psychology, 102(3), 403. https://doi.org/10.1037/apl0000106
23. Dollard, MF & Bakker, AB. (2010). Psychosocial safety climate as a precursor to conducive work environments, psychological health problems, and employee engagement. Journal of Occupational and Organizational Psychology, 83, 579–599. https://doi.org/10.1348/096317909X470690
24. Dollard, MF & Karasek, R. (2010). Building psychosocial safety climate: Evaluation of a socially coordinated PAR risk management stress prevention. In J Houdmont & S Leka

For peer review only

(Eds), Contemporary occupational health psychology (p. 208–234). Chichester: Wiley Blackwell. https://doi.org/10.1002/9780470661550.ch11

25. Law, R, Dollard, M.F., Tuckey, M.R., et al. (2011). Psychosocial safety climate as a lead indicator of workplace bullying and harassment, job resources, psychological health and employee engagement. Accident Analysis and Prevention, 43(5), 1782–1793. https://doi.org/10.1016/j.aap.2011.04.010
26. Idris, MA, Dollard, MF, Coward, J., et al. (2012). Psychosocial safety climate: conceptual distinctiveness and effect on job demands and worker psychological health. Safety Science, 50, 19–28. https://doi.org/10.1016/j.ssci.2011.06.005
27. Griffin, M, & Curcuruto, M. (2016). Safety Climate in Organizations. Annual Review of Organizational Psychology and Organizational Behavior, 3(1). https://doi.org/10.1146/annurev-orgpsych-041015-062414
28. McLinton, S. S., Afsharian, A., Dollard, M. F., et al. (2019). The dynamic interplay of physical and psychosocial safety climates in frontline healthcare. *Stress & Health, 35*(5), 650-664. https://doi.org/10.1002/smi.2898
29. Demerouti, E., Bakker, A., B., Nachreiner, F., et al. (2001). The job demands-resources model of burnout. Journal of Applied Psychology, 86, 499–512. PMID: 11419809
30. Dollard, M. F. & Bailey, T. (2014). The Australian workplace barometer: Psychosocial safety climate and working conditions in Australia. Samford Valley, QLD: Australian Academic Press Group Pty Ltd.
31. Hall, G. B., Dollard, M. F., Coward, J. (2010). Psychosocial Safety Climate: Development of the PSC-12. International Journal of Stress Management, 4, 353–383. https://doi.org/10.1037/a0021320
32. Karasek, R., Brisson, C., Kawakami, N., et al. (1998). The Job Content Questionnaire (JCQ): an instrument for internationally comparative assessments of psychosocial job characteristics. Journal of Occupational Health Psychology, 3(4), 322–355. https://doi.org/10.1037//1076-8998.3.4.322
33. Elo A.-L., Dallner M., Gamberale F., et al. (2000). Validation of the Nordic Questionnaire for Psychological and Social Factors at Work—QPSNordic. In M. Vartiainen, F. Avallone, & N. Anderson (Eds.), *Innovative theories, tools, and practices in work and organizational psychology* (pp. 47–57). Hogrefe & Huber Publishers.
34. Ørhede, E., Hottinen, V., Skogstad, A., et al. (2000). User's Guide for QPSNordic. General Nordic questionnaire for psychological and social factors at work. Copenhagen: Nordic Council of Minister.
35. Richman, J. A., Flaherty, J. A., & Rospenda, K. M. (1996). Perceived workplace harassment experiences and problem drinking among physicians: Broadening the stress/alienation paradigm. Addiction, 91(3), 391–403. https://doi.org/10.1046/J.1360-0443.1996.9133918.X
36. Kessler, R. & Mroczek, D. (1994). Final Version of our Non-specific Psychological Distress Scale. Ann Arbort (MI): Survey Research Center of the Institute for Social Research, University of Michigan.
37. Schaufeli, W., Leiter, M., Maslach, C. et al. (1996) Maslach Burnout Inventory-General Survey. In: Maslach, C., Jackson, S.E. and Leiter, M.P., Eds., The Maslach Burnout Inventory: Test Manual, Consulting Psychologists Press, Palo Alto, CA.
38. Schaufeli, W. B., Bakker, A. B., & Salanova, M. (2006). The Measurement of Work Engagement With a Short Questionnaire: A Cross-National Study. *Educational and Psychological Measurement, 66(*4), 701–716. https://doi.org/10.1177/0013164405282471
39. Reavley, N. J., Morgan, A. J. & Jorm, A. F. (2016). Disclosure of mental health problems: findings from an Australian national survey. Epidemiology and Psychiatric Sciences, 1-11. https://doi.org/10.1017/S204579601600113X
40. Spitzer, R L., Kroenke, K. & Williams, J. B. (1999). Validation and utility of a self-report version of PRIME-MD: The PHQ primary care study. JAMA. https://doi.org.10.1001/jamma.282.18.1737

For peer review only

41. Bailey, T. S., Dollard, M. F., McLinton, S. S., et al. (2015): Psychosocial safety climate, psychosocial and physical factors in the aetiology of musculoskeletal disorder symptoms and workplace injury compensation claims, Work & Stress: An International Journal of Work, Health & Organisations 29(2), 190-211. https://doi.org/10.1080/02678373.2015.1031855
42. Virtanen, M., Honkonen, T., Kivimaki, M. et al. (2007). Work stress, mental health and antidepressant medication findings from the Health 2000 Study. Journal of Affective Disorders, 98, 189–97. https://doi.org/10.1016/j.jad.2006.05.034

For peer review only

UniSA | Justice & Society

University of South Australia
Australia's University of Enterprise

Australian Workplace Barometer Survey (2021)

INFORMATION SHEET

Welcome to the Australian Workplace Barometer (AWB) survey.

Professor Maureen Dollard, Director of the PSC Observatory, for the University of South Australia is conducting the study with funding from the Australian Research Council.

This Australian Research Council national study aims to improve the health of Australian workers. Questions will be asked about working conditions, job security, psychological health, physical health, heart health, work engagement, satisfaction, fatigue, and workers' compensation.

We previously contacted you in 2014 about the Australian Workplace Barometer survey and you kindly gave us permission to contact you again regarding future surveys. Please complete the double-sided survey attached and return in the enclosed reply-paid envelope. Alternatively, if you wish to complete the survey online, please type the following link into your web browser http://bit.ly/AWB-Survey, ensuring to insert your Participant ID below into the survey link and complete online. Once you have completed the survey you will go into the draw to win $100.

For additional information about the Australian Workplace Barometer, please visit website https://bit.ly/AWB-InfoSheet

We greatly appreciate your support and contribution to the AWB project. If you experience any issues, please contact the AWB Team on 0422 643 737.

Kind regards
Dr Ali Afsharian
Research Associate
PSC Observatory / Centre for Workplace Excellence (CWeX)
University of South Australia
Email: ali.afsharian@unisa.edu.au

Participant's ID:

For peer review only - http://bmjopen.bmj.com/site/about/guidelines.xhtml

Australian Workplace Barometer (AWB) Survey

1. Are you still in paid employment or self-employed or a sub-contractor?
- ○ Employed (skip to Q.3)
- ○ Sub-contractor (skip to Q.5)
- ○ Self-employed
- ○ No longer in paid employment or self-employed or a subcontractor (go to Q.2)

If answered 'Self-employed', you will not be able to complete the remainder of the survey as the survey is for current employed workers and their workplace conditions. Thank you for your time.

2. What is the reason that you are no longer in paid employment or self-employed or a sub-contractor?
- ○ Retired
- ○ Made redundant
- ○ Maternity leave
- ○ Student
- ○ Other (specify): ___________________

You will not be able to complete the remainder of the survey as the survey is for current workers and their workplace conditions. Thank you for your time.

3. Is your employer currently receiving Job Keeper benefits for you?
- ○ Yes (go to Q.4)
- ○ No (skip to Q.6)
- ○ Not sure

4. Are you currently participating in work-related tasks or duties?
- ○ Yes (skip to Q.6)
- ○ No

If 'No', you will not be able to complete the remainder of the survey as the survey is for current workers and their workplace conditions. Thank you for your time.

5. Has your current contract involved working for the same employer for the past 4 months or longer?
- ○ Yes
- ○ No

If 'Yes', would you prefer an alternative employment status?
- ○ Permanent full-time
- ○ Permanent part-time
- ○ Casual/temporary (no annual/sick leave)
- ○ Fixed-term contract
- ○ Other (specify): ___________________

(skip to Q.7)

If 'No', you will not be able to complete the remainder of the survey as the survey is for current employed workers and their workplace conditions. Thank you for your time.

Employment

The following questions relate to your main job, that is the one in which you work most hours.

6. What is your employment status?
- ○ Permanent full-time
- ○ Permanent part-time
- ○ Casual/temporary (no annual/sick leave)
- ○ Fixed-term contract
- ○ Other (specify): ______________________

7. Has your employment status changed due to the Covid-19 pandemic?
- ○ No
- ○ Yes, from full-time to part time
- ○ Yes, from full-time to casual
- ○ Other (specify): ___________________

8. At this moment, which do you best identify with?
- ○ The majority of my work is done remotely
- ○ I normally split my time between home and or on-site, but now I'm only working remotely
- ○ I normally work on-site but now I'm working remotely
- ○ I am still working on-site

For peer review only - http://bmjopen.bmj.com/site/about/guidelines.xhtml

9. About how long have you been working for your current employer?
Enter years: ________________
Enter months: ________________

10. What is the main business, industry or service of your employer?
- ○ Accommodation, cafes and restaurants
- ○ Agriculture, forestry and fishing
- ○ Communications services
- ○ Construction
- ○ Cultural and recreational services
- ○ Education
- ○ Electricity, gas and water supply
- ○ Finance and insurance
- ○ Government administration and defence
- ○ Health and community services
- ○ Manufacturing
- ○ Mining
- ○ Personal and other services
- ○ Property and business services
- ○ Retail trade
- ○ Transport and storage
- ○ Wholesale trade
- ○ Other (specify): _____________

11. Is your employers business a…..?
- ○ A commonwealth or federal government department or agency
- ○ A state or local government agency
- ○ A not-for-profit, religious, or community organisation
- ○ A private sector business
- ○ Something else (specify): _____________

12. With regard to number of employees – what size organisation do you work in?
- ○ MICRO (less than 2)
- ○ SMALL (2-19)
- ○ MEDIUM (20-200)
- ○ LARGE (200+)
- ○ Don't know

13. Are you a member of a union or employee organisation that looks after employees' interests (e.g., Nursing Federation, Police Association)?
- ○ Yes
- ○ No
- ○ Don't know

14. Are you paid for any overtime you do?
- ○ Always
- ○ Sometimes
- ○ Never
- ○ Don't do any overtime

15. Which time period do you usually work?
- ○ Day
- ○ Evening
- ○ Night
- ○ Rotating shifts
- ○ Other (specify): ________________

16. Have you ever put in a worker's compensation claim?
- ○ Yes (go to Q.17)
- ○ No (skip to Q.21)

17. Did you lodge this claim in the last 12 months?
- ○ Yes (go to Q.18)
- ○ No (skip to Q.21)

18. Was the most recent claim for a stress or psychological injury?
- ○ Yes (go to Q.19)
- ○ No (skip to Q.20)

19. Reason for the most recent claim stress or psychological injury claim?
- ○ Exposure to a traumatic event
- ○ Exposure to workplace or occupational violence
- ○ Work related harassment and/or workplace bullying
- ○ Work Pressure
- ○ Other mental stress factors
- ○ Other harassment
- ○ Other (specify): ______________

(skip to Q.21)

20. Was the most recent claim for neck, back, or joint pain or muscle injury?
- ○ Yes
- ○ No

For peer review only - http://bmjopen.bmj.com/site/about/guidelines.xhtml

**Job Demands - Psychological Demands**<br>Below are some statements about your work. Could you please tick below if you disagree or agree and to what level ...?				
	**Strongly Disagree**	**Disagree**	**Agree**	**Strongly Agree**
**21.** My job requires working very fast.	○	○	○	○
**22.** My job requires working very hard.	○	○	○	○
**23.** I am not asked to do an excessive amount of work.	○	○	○	○
**24.** I have enough time to get the job done.	○	○	○	○
**25.** Some demands I face at work are in conflict with other demands at work.	○	○	○	○

**Job Demands - Physical Demands**<br>Below are some statements about your work. Could you please tick below if you disagree or agree and to what level ...?				
	**Strongly Disagree**	**Disagree**	**Agree**	**Strongly Agree**
**26.** My job requires lots of physical effort.	○	○	○	○
**27.** I am often required to move or lift very heavy loads on my job.	○	○	○	○
**28.** I am often required to work for long periods with my body in physically awkward positions.	○	○	○	○

**Workplace Physical Risk Factors**<br>I am required to:				
	**Almost never**	**Sometimes**	**Often**	**Almost always**
**29.** Keep repeating the same movements, every minute or so.	○	○	○	○
**30.** Lift or carry things that are moderately (or very) heavy.	○	○	○	○
**31.** Push or pull things using some force.	○	○	○	○
**32.** Work in twisted or awkward postures.	○	○	○	○
**33.** Squat or kneel while you work.	○	○	○	○

**Emotional Demands**				
	**Strongly Disagree**	**Disagree**	**Agree**	**Strongly Agree**
**34.** My work is emotionally demanding.	○	○	○	○
**35.** My work places me in emotionally challenging situations.	○	○	○	○
**36.** My work requires suppressing my genuine emotion.	○	○	○	○
**37.** I have to act the way people think a person in my position should act.	○	○	○	○

For peer review only - http://bmjopen.bmj.com/site/about/guidelines.xhtml

**Organisational Characteristics - Demands Organisational Change**<br>The next set of questions concerns things that you may or may not experience in your work.				
	**Strongly Disagree**	**Disagree**	**Agree**	**Strongly Agree**
**38.** In your company/organisation, there have been changes such as restructuring, downsizing, and layoffs that have significantly affected your job.	○	○	○	○
**39.** In your company/organisation, new policies and procedures designed to cut costs are constantly being introduced where you work.	○	○	○	○
**40.** In your company/organisation, there is frequent management turnover.	○	○	○	○

**Organisational Harassment**<br>Sometimes in your workplace you may experience or witness harassment from your manager/supervisor or colleagues. Can you please answer with the best response to these statements?					
	**Very rarely/never**	**Rarely**	**Sometimes**	**Often**	**Very often/ always**
**41.** I have experienced unwanted sexual advances.	○	○	○	○	○
**42.** I have experienced discomfort listening to sexual humour.	○	○	○	○	○
**43.** I have experienced unfair treatment because of my gender.	○	○	○	○	○
**44.** Negative comments have been made regarding my ethnic or racial background.	○	○	○	○	○
**45.** I have been sworn at and or yelled at.	○	○	○	○	○
**46.** I have been humiliated in front of others.	○	○	○	○	○
**47.** I have experienced being physically assaulted and or threatened by members of the organisation.	○	○	○	○	○
**48.** I have experienced being physically assaulted and or threatened, by a client or patient.	○	○	○	○	○

For peer review only

For peer review only - http://bmjopen.bmj.com/site/about/guidelines.xhtml

Organisational Bullying
Bullying is a problem at some workplaces and for some workers. To label something as bullying, the offensive behaviour has to occur repeatedly over a period of time, and the person confronted has to experience difficulties defending him or herself. The behaviour is not bullying if two parties of approximate equal "strength" are in conflict or the incident is an isolated event.

49. Have you been subjected to bullying at the workplace during the last six months?
- Yes (go to Q.50)
- No (skip to Q.53)

50. how often were you exposed to these bullying behaviours overall?
- Daily
- At least once per week
- At least once per month
- Rarely
- Never

51. How long were you exposed to these bullying behaviours overall?
- Less than one month
- 1-6 months
- 7-12 months
- 1-2 years
- More than 2 years

52. Was the bully a …?
- A supervisor
- A co-worker
- Neither
- Both

Work-Family Conflict
Below are some statements about how work can affect your home and personal life.
Could you please tick below if you disagree or agree and to what level ...?

	Strongly Disagree	Disagree	Slightly Disagree	Neither Agree nor Disagree	Slightly Agree	Agree	Strongly Agree
**53.** The demands of my work interfere with my home and family life.	○	○	○	○	○	○	○
**54.** My job produces strain that makes it difficult to fulfil family duties.	○	○	○	○	○	○	○
**55.** Due to work-related duties, I have to make changes to my plans for family activities.	○	○	○	○	○	○	○

For peer review only - http://bmjopen.bmj.com/site/about/guidelines.xhtml

Psychosocial Safety Climate
The questions and statements in this section are about the psychological health and safety at your workplace. Could you please tick below if you disagree or agree and to what level ...?

	Strongly Disagree	Disagree	Neither Disagree or Agree	Agree	Strongly Agree
**56.** In my workplace senior management acts quickly to correct problems/issues that affect employees' psychological health.	○	○	○	○	○
**57.** Senior management acts decisively when a concern of an employees' psychological status is raised.	○	○	○	○	○
**58.** Senior management show support for stress prevention through involvement and commitment.	○	○	○	○	○
**59.** Psychological well-being of staff is a priority for this organisation.	○	○	○	○	○
**60.** Senior management clearly considers the psychological health of employees to be of great importance.	○	○	○	○	○
**61.** Senior management considers psychological health to be as important as productivity.	○	○	○	○	○
**62.** There is good communication here about psychological safety issues which affect me.	○	○	○	○	○
**63.** Information about workplace psychological well-being is always brought to my attention by my manager/supervisor.	○	○	○	○	○
**64.** My contributions to resolving occupational health and safety concerns in the organisation are listened to.	○	○	○	○	○
**65.** Participation and consultation in psychological health and safety occurs with employees', unions and health and safety representatives in my workplace.	○	○	○	○	○
**66.** Employees are encouraged to become involved in psychological safety and health matters.	○	○	○	○	○
**67.** In my organisation, the prevention of stress involves all levels of the organisation.	○	○	○	○	○

For peer review only

For peer review only - http://bmjopen.bmj.com/site/about/guidelines.xhtml

**Resources - Skill Discretion**<br>The next set of questions concerns some characteristics of your work. Please answer with the best response that suits your workplace.				
	**Strongly Disagree**	**Disagree**	**Agree**	**Strongly Agree**
**68.** My job requires that I learn new things.	○	○	○	○
**69.** My job involves a lot of repetitive work.	○	○	○	○
**70.** My job requires me to be creative.	○	○	○	○
**71.** My job requires a high level of skill.	○	○	○	○
**72.** I get to do a variety of different things in my job.	○	○	○	○
**73.** I have an opportunity to develop my own special abilities.	○	○	○	○
**Decision Authority**				
	**Strongly Disagree**	**Disagree**	**Agree**	**Strongly Agree**
**74.** My job allows me to make a lot of decisions on my own.	○	○	○	○
**75.** In my job, I have very little freedom to decide how I do my work.	○	○	○	○
**76.** I have a lot to say about what happens in my job.	○	○	○	○
**Macro-Decision Latitude**				
	**Strongly Disagree**	**Disagree**	**Agree**	**Strongly Agree**
**77.** In my company/organisation, I have significant influence over decisions made by my work team or department.	○	○	○	○
**78.** In my company/organisation, all parties are represented for major decisions.	○	○	○	○
**79.** In my company/organisation, we are always informed well in advance about major decisions.	○	○	○	○

**Supervisor Social Support**				
	**Strongly Disagree**	**Disagree**	**Agree**	**Strongly Agree**
**80.** My supervisor/manager is concerned about the welfare of those under him/her.<br>I have no supervisor/manager (skip to Q.83)	○	○	○	○
**81.** My supervisor/manager is helpful in getting the job done.	○	○	○	○
**82.** I am treated with respect by my supervisor/manager.	○	○	○	○

For peer review only - http://bmjopen.bmj.com/site/about/guidelines.xhtml

**Co-worker Social Support**				
	**Strongly Disagree**	**Disagree**	**Agree**	**Strongly Agree**
**83.** People I work with are friendly.<br>I have no coworkers (skip to Q.86)	○	○	○	○
**84.** People I work with are helpful in getting the job done.	○	○	○	○
**85.** I am treated with respect by my co-workers.	○	○	○	○

**Recovery**							
	**Strongly Disagree**	**Disagree**	**Slightly Disagree**	**Neither Agree nor Disagree**	**Slightly Agree**	**Agree**	**Strongly Agree**
**86.** I rarely recover my strength fully between work periods.	○	○	○	○	○	○	○
**87.** I'm often still feeling fatigued from one work period by the time I start the next one.	○	○	○	○	○	○	○

**Organisational Characteristics - Resources Organisational Rewards**				
	**Strongly Disagree**	**Disagree**	**Agree**	**Strongly Agree**
**88.** Considering all my efforts and achievements, I receive the respect and prestige I deserve at work.	○	○	○	○
**89.** Considering all my efforts and achievements, my job prospects are adequate.	○	○	○	○
**90.** Considering all my efforts and achievements, my salary/income is adequate.	○	○	○	○
**91.** My job security is poor.	○	○	○	○

For peer review only

Psychosocial Safety Climate Policy

92. Does your establishment have a procedure to deal with work-related stress?
- o Yes
- o No
- o Work-related stress is not an issue in our establishment
- o Don't know

93. Is there a procedure in place to deal with bullying or harassment?
- o Yes
- o No
- o These problems are not an issue in our establishment
- o Don't know

94. And do you have a procedure to deal with work-related violence?
- o Yes
- o No
- o Work-related violence is not an issue in our establishment
- o Don't know

95. What about the role of employees: Have they been consulted regarding measures to deal with psychosocial risks?
- o Yes
- o No
- o Don't know

96. Are employees encouraged to participate actively in the implementation and evaluation of the measures?
- o Yes
- o No
- o Don't know

Job Redesign

In the last 3 years, has your establishment used any of the following measures to deal with psychosocial risks?

97. Changes to the way work is organised?
- o Yes
- o No
- o Don't know

98. A redesign of the work area?
- o Yes
- o No
- o Don't know

99. Set-up of a conflict resolution procedure?
- o Yes
- o No
- o Don't know

Engagement & Happiness

We also need to know about your levels of engagement in your work. Please answer with the best response.

Vigour

100. At my work, I feel bursting with energy.
- o Never
- o 1-3 times per year or less
- o Once a month
- o A few times a month
- o Once a week
- o A few times a week
- o Every day

Dedication

101. I am enthusiastic about my job.
- o Never
- o 1-3 times per year or less
- o Once a month
- o A few times a month
- o Once a week
- o A few times a week
- o Every day

Absorption

102. I am immersed in my work.
- o Never
- o 1-3 times per year or less
- o Once a month
- o A few times a month
- o Once a week
- o A few times a week
- o Every day

Happiness

The next question is about how you rate your general happiness. On a scale from 0 to 10 where 0 means "extremely unhappy" and 10 means "extremely happy"……

Overall, how happy are you?
- o Enter no. ____________________
- o Don't know

For peer review only - http://bmjopen.bmj.com/site/about/guidelines.xhtml

Mental Health

We also need to know some information about your mental and physical health. Please answer as best you can by selecting the best option.

Emotional Exhaustion

	Never	A few times a year or less	Once a month or less	A few times a month	Once a week	A few times a week	Every day
**103.** I feel emotionally drained from my work.	○	○	○	○	○	○	○
**104.** I feel used up at the end of the workday.	○	○	○	○	○	○	○
**105.** I feel tired when I get up in the morning and have to face another day on the job.	○	○	○	○	○	○	○
**106.** Working all day is a strain for me.	○	○	○	○	○	○	○
**107.** I feel burned out from my work.	○	○	○	○	○	○	○

Psychological Distress

	None of the time	A little of the time	Some of the time	Most of the time	All of the time
**108.** In the past four weeks, about how often did you feel tired out for no good reason?	○	○	○	○	○
**109.** In the past four weeks, about how often did you feel nervous?	○	○	○	○	○
**110.** In the past four weeks, about how often did you feel so nervous that nothing could calm you down?	○	○	○	○	○
**111.** In the past four weeks, about how often did you feel hopeless?	○	○	○	○	○
**112.** In the past four weeks, about how often did you feel restless or fidgety?	○	○	○	○	○
**113.** In the past four weeks, about how often did you feel so restless you could not sit still?	○	○	○	○	○
**114.** In the past four weeks, about how often did you feel depressed?	○	○	○	○	○
**115.** In the past four weeks, about how often did you feel everything was an effort?	○	○	○	○	○
**116.** In the past four weeks, about how often did you feel so sad that nothing could cheer you up?	○	○	○	○	○
**117.** In the past four weeks, about how often did you feel worthless?	○	○	○	○	○

For peer review only - http://bmjopen.bmj.com/site/about/guidelines.xhtml

Depression				
	Not at all	Several days	More than half the days	Nearly every day
**118.** During the last month, how often were you bothered by little interest or pleasure in doing things?	○	○	○	○
**119.** During the last month, how often were you bothered by feeling down, depressed, or hopeless?	○	○	○	○
**120.** During the last month, how often were you bothered by trouble falling or staying asleep OR sleeping too much?	○	○	○	○
**121.** During the last month, how often were you bothered by feeling tired or having little energy?	○	○	○	○
**122.** During the last month, how often were you bothered by poor appetite or overeating?	○	○	○	○
**123.** During the last month, how often were you bothered by feeling bad about yourself-or that you are a failure or have let yourself or your family down?	○	○	○	○
**124.** During the last month, how often were you bothered by trouble concentrating on things, such as reading a newspaper or watching television?	○	○	○	○
**125.** During the last month, how often were you bothered by moving or speaking so slowly that other people could have noticed OR – the opposite – being so fidgety or restless that you have been moving around a lot more than usual?	○	○	○	○
**126.** During the last month, how often were you bothered by thoughts that you would be better off dead or thoughts of hurting yourself in some way?	○	○	○	○

For peer review only

Stigma and Discrimination

127. In the last 12 months…
Do you think you have had emotional or mental health problems?
- ○ Yes (go to Q.128)
- ○ No (skip to Q.135)
- ○ Don't know (go to Q.135)

128. In the last 12 months…
Have you ever discussed your anxiety or depression with your supervisors and or managers?
- ○ Yes
- ○ No

129. Have you ever discussed your anxiety or depression with your co-worker?
- ○ Yes
- ○ No

	Not at all	A little	Moderately	A lot	Don't know
**130.** Have any of your supervisors and or managers avoided you because of the emotional or mental health problems?	○	○	○	○	○
**131.** Have any of your coworkers avoided you because of the emotional or mental health problems?	○	○	○	○	○
**132.** Have any of your supervisors and or managers treated you unfairly or discriminated against you because of your mental health problems?	○	○	○	○	○
**133.** Have any of your co-workers treated you unfairly or discriminated against you because of your mental health problems?	○	○	○	○	○
**134.** Because of your mental health or emotional problems have you been treated more positively by your employer?	○	○	○	○	○

For peer review only - http://bmjopen.bmj.com/site/about/guidelines.xhtml

Life Events

We now would like you to think about major events that have happened in your life over the past 12 months.

135. Did any of these events happen to you in the past 12 months? (please tick any event that applies to you)

- ◻ Death of spouse or child
- ◻ Separated from spouse or long-term partner
- ◻ Major worsening in financial situation (e.g., went bankrupt)
- ◻ Serious personal injury or illness to self
- ◻ Victim of physical violence (e.g., assault)
- ◻ Got married
- ◻ Partner or I gave birth to, or adopted, a new child
- ◻ Major improvement in financial situation (e.g., won lottery, received an inheritance)

136. Has a doctor ever diagnosed you with depression or anxiety?

- ○ Yes
- ○ No

137. Have you taken any prescription medications for your mental health in the last 12 months?

- ○ Yes
- ○ No
- ○ Don't know

138. Have you taken any of the following prescription medications in the past 12 months? (please tick any that apply to you)

- ◻ Sleeping tablets or capsules
- ◻ Tablets or capsules for anxiety or nerves
- ◻ Tranquillisers
- ◻ Antidepressants
- ◻ Mood stabilisers
- ◻ Other medications for your mental health
- ◻ None of the above
- ◻ Don't know

139. Have you taken this in the last month?

- ◻ Sleeping tablets or capsules
- ◻ Tablets or capsules for anxiety or nerves
- ◻ Tranquillisers
- ◻ Antidepressants
- ◻ Mood stabilisers
- ◻ Other medications for your mental health
- ◻ None of the above
- ◻ Don't know

For peer review only - http://bmjopen.bmj.com/site/about/guidelines.xhtml

Physical Health - General Health

140. During the past 7 days how much were you bothered by back or neck pain?
- Not at all
- A little
- Some
- A lot

141. During the past 7 days how much were you bothered by pain in your arms, legs, or joint areas like your knee or hips?
- Not at all
- A little
- Some
- A lot

142. During the past 7 days how much were you bothered by muscle soreness?
- Not at all
- A little
- Some
- A lot

143. In general, would you say your health is….?
- Excellent
- Very good
- Good
- Fair
- Poor

144. Have you had a significant physical injury from inside of work in the past 12 months?
- Yes
- No

145. Did you make a compensation claim?
- Yes
- No

Cardio-Vascular Health

146. In the past two years have you had your blood pressure taken by a health professional or used a self-administered machine?
- Yes
- No

IF yes, what was your blood pressure?
- Low
- Normal
- High
- Don't know

147. In the past two years have you consulted a health professional with regard to chest pain, or any other cardiovascular related health problem – such as myocardial infarction; angina; stroke; or hypertension?
- Yes
- No
- Don't know

IF yes, what was the diagnosis?
- Myocardial Infarction
- Angina
- Stroke
- Hypertension
- Other (specify)/Or if more than one, mention here ________________
- Don't know
- Not Stated
- Nothing

Work Outcomes - Job Satisfaction

148. Taking everything into consideration, how do you feel about your job as a whole?
- I'm extremely dissatisfied
- I'm very dissatisfied
- I'm moderately dissatisfied
- I'm not sure
- I'm moderately satisfied
- I'm very satisfied
- I'm extremely satisfied

Work Outcomes – Intention to Leave

149. Over this year I intend to leave this organisation.
- Strongly Agree
- Agree
- Neutral
- Disagree
- Strongly Disagree

For peer review only - http://bmjopen.bmj.com/site/about/guidelines.xhtml

Work Outcomes - Productivity

150. On a scale from 0 to 10 where 0 is the worst job performance anyone could have at your job and 10 is the performance of a top worker, how would you rate the usual performance of most workers in a job similar to yours?
Enter number here: ______________

151. Using the same 0-to-10 scale, how would you rate your overall job performance on the days you worked during the past 4 weeks (28 days)?
Enter number here: _______________

152. Using the same 0-to-10 scale, where 0 is no affect and 10 is the most affect, during the last 4 weeks (28 days) how much did your health problems affect your productivity at work?
Enter number here: __________________

Work Outcomes - Absenteeism

153. How many hours does your employer expect you to work in a typical 7-day week?
Note: If it varies, estimate the average. If more than 97, enter 97. If none - enter 0.
Enter hours: ______________________

154. In the past 4 weeks (28 days), how many days did you, miss an entire work day because of problems with your physical or mental health? (Please include only days missed for your own health not someone else's health?
Enter number of days (0-28): ________

155. About how many hours altogether did you work in the past 4 weeks?
Enter number of hours: _____________

Employment

The next questions are about your main job, that is the one in which you work most hours.

156. What is your occupation in this job – what are your main duties?

- o Managers or administrator (go to Q.157)
- o Professional work (go to Q.158)
- o Technician or associate professional (go to Q.159)
- o Tradesperson or related work (go to Q.160)
- o Advanced clerical, sales or service work (go to Q.161)
- o Intermediate clerical, sales or service work (go to Q.162)
- o Intermediate plant operator/transport (go to Q.163)
- o Elementary clerical, sales or service work (go to Q.164)
- o Labourer or related work (go to Q.165)
- o Other (please specify) _____________ (go to Q.166)

157. What best describes this work?

- o General Managers and Administrators
- o Miscellaneous Generalist Managers
- o Resource Managers
- o Engineering, Distribution and Process Managers
- o Sales and Marketing Managers
- o Miscellaneous Specialist Managers
- o Farmers and Farm Managers
- o Other (please specify) _____________ (go to Q.166)

158. What best describes this work?

- o Natural and Physical Science Professionals
- o Building and Engineering Professionals
- o Accountants, Auditors and Corporate Treasurers
- o Sales, Marketing and Advertising Professionals
- o Computing Professionals
- o Miscellaneous Business and Information Professionals
- o Medical Practitioners
- o Nursing Professionals
- o Miscellaneous Health Professionals
- o School Teachers
- o University and Vocational Education Teachers
- o Miscellaneous Education Professionals
- o Social Welfare Professionals
- o Miscellaneous Social Professionals
- o Artists and Related Professionals
- o Miscellaneous Professionals
- o Other (please specify) _____________ (go to Q.166)

For peer review only - http://bmjopen.bmj.com/site/about/guidelines.xhtml

For peer review only

159. What best describes this work?
- ○ Medical and Science Technical Officers
- ○ Building and Engineering Associate Professionals
- ○ Finance Associate Professionals
- ○ Miscellaneous Business and Administration Associate Professionals
- ○ Shop Managers
- ○ Hospitality and Accommodation Managers
- ○ Miscellaneous Managing Supervisors (Sales and Service)
- ○ Enrolled Nurses
- ○ Welfare Associate Professionals
- ○ Miscellaneous Health and Welfare Associate Professionals
- ○ Police Officers
- ○ Miscellaneous Associate Professionals
- ○ Other (please specify) _______________ (go to Q.166)

160. What best describes this work?
- ○ Mechanical Engineering Tradespersons
- ○ Fabrication Engineering Tradespersons
- ○ Automotive Tradespersons
- ○ Electrical and Electronics Tradespersons
- ○ Structural Construction Tradespersons
- ○ Final Finishes Construction Tradespersons
- ○ Plumbers
- ○ Food Tradespersons
- ○ Skilled Agricultural Workers
- ○ Horticultural Tradespersons
- ○ Printing Tradespersons
- ○ Wood Tradespersons
- ○ Hairdressers
- ○ Textile, Clothing and Related Tradespersons
- ○ Miscellaneous Tradespersons and Related Workers
- ○ Other (please specify) _______________ (go to Q.166)

161. What best describes this work?
- ○ Secretaries and Personal Assistants
- ○ Advanced Numerical Clerks
- ○ Miscellaneous Advanced Clerical and Service Workers
- ○ Other (please specify) _______________ (go to Q.166)

162. What best describes this work?
- ○ General Clerks
- ○ Keyboard Operators
- ○ Receptionists
- ○ Intermediate Numerical Clerks
- ○ Material Recording and Despatching Clerks
- ○ Miscellaneous Intermediate Clerical Workers
- ○ Intermediate Sales and Related Workers
- ○ Carers and Aides
- ○ Hospitality Workers
- ○ Miscellaneous Intermediate Service Workers
- ○ Other (please specify) _______________ (go to Q.166)

163. What best describes this work?
- ○ Mobile Plant Operators
- ○ Intermediate Stationary Plant Operators
- ○ Intermediate Textile, Clothing and Related Machine Operators
- ○ Miscellaneous Intermediate Machine Operators
- ○ Road and Rail Transport Drivers
- ○ Intermediate Mining and Construction Workers
- ○ Miscellaneous Intermediate Production and Transport Workers
- ○ Other (please specify) _______________ (go to Q.166)

164. What best describes this work?
- ○ Elementary Clerks
- ○ Sales Assistants
- ○ Miscellaneous Elementary Sales Workers
- ○ Elementary Service Workers
- ○ Other (please specify) _______________ (go to Q.166)

165. What best describes this work?
- ○ Cleaners
- ○ Process Workers
- ○ Product Packagers
- ○ Mining, Construction and Related Labourers
- ○ Agricultural and Horticultural Labourers
- ○ Elementary Food Preparation and Related Workers
- ○ Miscellaneous Labourers and Related Workers
- ○ Other (please specify) _______________ (go to Q.166)

For peer review only - http://bmjopen.bmj.com/site/about/guidelines.xhtml

Now to finish with some general questions.

166. Do you identify as ….?

- Male
- Female
- Non binary
- Transgender Male
- Transgender Female
- Other (please specify): ______

167. Including yourself how many people aged 18 or over, who are currently in paid employment, live in this household?
Enter number: ____________________

168. How many children under 18 live in your household?
Enter number: __________________

169. What state do you live in?

- NSW
- WA
- SA
- ACT
- TAS
- NT
- QLD
- VIC
- Other: _______________

170. What is the postcode of the house?
____

171. What town or suburb do you live in?
Enter town/suburb: ______________

172. What state do you work in?

- NSW
- WA
- SA
- ACT
- TAS
- NT
- QLD
- VIC
- Outside of Australia
- Other (please specify): ___________

173. The Australian Council of Trade Unions (ACTU) in partnership with The University of South Australia (UniSA) and Employers Mutual Limited (EML) are co-hosting the Mind Your Head Campaign funded by WorkSafe Victoria's WorkWell Mental Health Improvement.
Have you heard/received any information about this campaign? (please disregard if not from VIC)

- Yes
- No

If 'Yes' what was the source of this information

- Workplace communications
- Media communications
- External/personal communications
- Other (please specify): _____________

174. What is your marital status?

- Married
- Living with partner
- Separated
- Divorced
- Widowed
- Never married

175. What best describes the highest educational qualification you have obtained?

- Still at school
- Left school at 16 years or less
- Left school after age 16
- Left school after age 16 but still studying
- Trade / Apprenticeship
- Certificate / Diploma
- Bachelor degree or higher
- Other (specify): _______________

176. We are interested to know if there is any relation to income levels and job stress risk. Before tax is taken out, which of the following best describes your income from your main job in the last 12 months?

- Up to $12,000
- $12,001 - $20,000
- $20,001 - $30,000
- $30,001 - $40,000
- $40,001 - $50,000
- $50,001 - $60,000
- $60,001 - $80,000
- $80,001 - $100,000
- $100,001 - $150,000
- $150,001 - $200,000
- More than $200,000

For peer review only - http://bmjopen.bmj.com/site/about/guidelines.xhtml

In order for us to make really good use of your answers it would be of great benefit to ask you the same questions at a later date. Would you be willing to be re-contacted about doing this survey again in about a year's time? You are not obliged to say yes if we call. You can decide then.

177. Are you willing to be re-contacted?
- Yes (full name, please): ______________
- Best contact number: ______________
- Email: ______________________________
- No

178. Could we have your current address?
- Yes (please enter the address in below) ______________
- No

In this kind of research, it is important to group data so it better reflects the work situation rather than the individual situation. By using organisational names we can group data and link workplace data to other worker health related data bases. We can answer questions like "In general, do organisations with high work pressure have more worker injuries?"

179. Could you give the name of your organisation so we can do this? The organisation/employer will be confidential in any report.
Enter organisation or business name ______________

180. What the suburb of your employer is?
Enter town/suburb: ________________

181. Is this the same organisation that you were at when we last interviewed you?
- Yes (skip to Q.183)
- No (go to Q.182)
- Can't remember (skip to Q.183)

182. If it is not the same organisation could we have the name and suburb of the organisation you worked at previously?
Enter organisation: ________________
Enter suburb: _____________________

We would also like to investigate how work conditions relate to mental health and related medications, such as "is work stress related to anti-depressant use in Australia?"
To facilitate this important research, we ask your permission to securely send your AWB personal information (along with other participant's information) to the Australian Institute of Health and Welfare to match to the Pharmaceutical Benefits Scheme data to create an anonymous research dataset.

183. Do you consent for us to securely send your personal AWB information (the information you have provided in this survey) to the Australian Institute of Health and Welfare to match to medication data in the Pharmaceutical Benefits Scheme and create an anonymous research dataset?
- Yes
- No

That was the final question. Thank you again for your time and support for the Australian Workplace Barometer project. We really appreciate it.

During this survey you were to think of some negative and positive experiences. You have also been asked some questions about if you have experienced any levels of depression. If you feel distressed as a result of any of these questions we offer you the telephone numbers below, should you need to discuss some of these concerns with a qualified professional.

Beyond Blue Support Service Line: 1300 224 636
Lifeline: 13 11 14

For peer review only

For peer review only - http://bmjopen.bmj.com/site/about/guidelines.xhtml
